# IGF-I induced genes in stromal fibroblasts predict the clinical outcome of breast and lung cancer patients

**DOI:** 10.1186/1741-7015-8-1

**Published:** 2010-01-05

**Authors:** Michal Rajski, Rosanna Zanetti-Dällenbach, Brigitte Vogel, Richard Herrmann, Christoph Rochlitz, Martin Buess

**Affiliations:** 1 Department of Biomedicine, University of Basel, Hebelstrasse 20, CH-4031 Basel, Switzerland; 2 Division of Medical Oncology, Department of Internal Medicine, St Claraspital, Kleinriehenstrasse 20, CH-4016 Basel, Switzerland; 3 Division of Medical Oncology, Department of Internal Medicine, University Hospital Basel, Petersgraben 4, CH-4031 Basel, Switzerland; 4 Department of Gynecology and Obstetrics, University Hospital Basel, Petersgraben 4, CH-4031 Basel, Switzerland

## Abstract

**Background:**

Insulin-like growth factor-1 (IGF-I) signalling is important for cancer initiation and progression. Given the emerging evidence for the role of the stroma in these processes, we aimed to characterize the effects of IGF-I on cancer cells and stromal cells separately.

**Methods:**

We used an *ex vivo *culture model and measured gene expression changes after IGF-I stimulation with cDNA microarrays. *In vitro *data were correlated with *in vivo *findings by comparing the results with published expression datasets on human cancer biopsies.

**Results:**

Upon stimulation with IGF-I, breast cancer cells and stromal fibroblasts show some common and other distinct response patterns. Among the up-regulated genes in the stromal fibroblasts we observed a significant enrichment in proliferation associated genes. The expression of the IGF-I induced genes was coherent and it provided a basis for the segregation of the patients into two groups. Patients with tumours with highly expressed IGF-I induced genes had a significantly lower survival rate than patients whose tumours showed lower levels of IGF-I induced gene expression (*P *= 0.029 - Norway/Stanford and *P *= 7.96e-09 - NKI dataset). Furthermore, based on an IGF-I induced gene expression signature derived from primary lung fibroblasts, a separation of prognostically different lung cancers was possible (*P *= 0.007 - Bhattacharjee and *P *= 0.008 - Garber dataset).

**Conclusion:**

Expression patterns of genes induced by IGF-I in primary breast and lung fibroblasts accurately predict outcomes in breast and lung cancer patients. Furthermore, these IGF-I induced gene signatures derived from stromal fibroblasts might be promising predictors for the response to IGF-I targeted therapies.

See the related commentary by Werner and Bruchim: http://www.biomedcentral.com/1741-7015/8/2

## Background

There is a considerable amount of evidence that the insulin-like growth factor (IGF) family is important for cancer development and progression and IGF signalling is known to involve complex regulatory networks with numerous interacting ligands, receptors and binding proteins [1,2]. IGF-I, the first ligand of the family, may act as a tissue growth factor in an autocrine or paracrine manner or as a circulating hormone [3]. An elevated IGF-I level in the plasma is linked to an increased risk of developing ductal carcinoma *in situ *of the breast, invasive breast cancer, colorectal cancer, prostate cancer and lung cancer [4-9].

IGF-I signalling is crucial for tumour progression because it is involved in cell proliferation, differentiation, migration and survival [2,3,10-13]. On the molecular level, IGF-I is one of the factors that enables cells to pass the G1-S checkpoint in the cell cycle [14]. Normal mammary epithelial cells can be maintained and will proliferate with IGF-I in serum free cell culture media, underscoring the IGF-I's importance for the growth of breast epithelial cells [15,16]. In combination with mammogenic hormones, IGF induces ductal growth in mammary gland explant cultures [17]. Furthermore, IGF-I and IGF-II can suppress apoptosis of mammary epithelial cells induced by serum withdrawal [12]. *In vivo*, the involution of mammary glands is delayed in mice over-expressing human IGF-I due to reduced alveolar apoptosis [18]. During mammary gland development, IGF-I synergizes with estrogen in terminal end bud formation [19]. Finally, both IGF-I and IGF-II provide cancer cells with radioprotection and resistance to chemotherapeutic agents [20,21].

Further highlighting the importance of the IGF-I axis, the IGF-I receptor (IGF-IR) is crucial in cancer development and progression. The IGF-IR was found to be over-expressed and highly activated in malignant breast tumours compared with normal breast tissue [22,23]. Patients bearing an oestrogen receptor negative breast tumour have a worse prognosis when their tumour is positive for IGF-IR [24]. The functional importance of IGF-IR has been shown *in vitro *by inhibiting the receptor signalling which results in cancer cell apoptosis. *In vivo*, the inhibition of IGF-IR signalling prevents tumour formation in nude mice [1,25]. Moreover, IGF-IR-deficient fibroblasts cannot be transformed by viral or cellular oncogenes [26], supporting the importance of IGF-IR signalling in tumourigenesis.

That IGFs are involved in breast cancer migration and invasion has been demonstrated using dominant-negative IGF-IR constructs in MDA-435 breast cancer cells *in vitro *and *in vivo *[27]. Another experiment revealed that IGF-I stimulates cell motility, but not proliferation, in MDA-231BO cells in which the predominant adaptor protein for IGF-IR is the insulin receptor substrate 2 (IRS2) instead of the insulin receptor substrate 1 (IRS1). Further evidence supporting the involvement of IGF-IR and IRS2 axis in motility and metastasis comes from *in vivo *data. The mating of mice expressing the PyV-MT (polyomavirus middle T) oncogene, which induces breast cancer, with IRS2 *null *animals instead of wild-type animals results in their offspring showing a decrease in the formation of metastasis [28]. Thus, IGF-I is emerging as an important factor in tumourigenesis as a cell death inhibitor and a proliferation enhancer. Its involvement in tumour progression, metastasis and resistance to anti-neoplastic therapies makes it a promising drug target which is currently being examined in numerous clinical trials [29].

So far, the attention on IGF-I has focused on mitogenic and tumourigenic signalling in cancer cells [9,29,30]. With the increasing knowledge of the role of the tumour stroma in cancer initiation and progression, the role of IGF-I signalling in the stroma is of equal interest. In tumours, most of the IGF-I mRNA is localized in the stromal cells [31], especially fibroblasts [32], whereas most of the IGF-IR mRNA is in the tumour cells [33] which indicates that IGF-I produced in the stroma influences the tumour cells. However, there is evidence that IGF-I also influences the stroma. Stromal cells respond to IGF-I stimulation with increased proliferation, as do fibroblasts [34,35] and microvascular endothelial cells [36].

In addition to the response of the tumour cells to IGF-I, we specifically focused on the response of the stromal cells to this growth factor. Bendall *et al. *recently showed that the IGF-IR axis is involved in the establishment of the stem cell niche [37]. Blocking IGF-II/IGF-IR reduces the survival and clonogenicity of human embryonic stem cells (ES). Similarly, IGF-II alone is sufficient to maintain human ES cells in culture. In this system, IGF-II was expressed by autologously human-ES-cell-derived fibroblast-like cells.

In our study, we explore the role that IGF-I stimulation plays in cancer and stroma cells. We study the molecular changes that occur in primary normal and cancer-associated fibroblasts when they are stimulated with IGF-I. Furthermore, we hypothesized that gene expression changes in this system might be of prognostic significance in human cancer.

In this report, we show that primary normal and carcinoma-associated breast fibroblasts are sensitive to IGF-I. In addition, fibroblasts of different origin show a unified response to IGF-I. We also demonstrate that genes up-regulated in primary breast and lung fibroblasts may have prognostic significance in human breast cancer and lung adenocarcinomas.

## Results

### Effects of IGF-I on gene expression in breast cancer cells and stromal fibroblasts

In order to characterize the effects of IGF-I on tumour and stromal cells, we stimulated pre-starved MCF-7 cells and CCL-171 fibroblasts with 50 ng/ml IGF-I (a concentration within the physiological range) for 24 h. We then profiled gene expression changes using human exonic evidence-based oligonucleotide (HEEBO) microarrays. After stimulation, total RNA was extracted and amplified using a modified Eberwine procedure. The amplified RNA was labelled with the fluorescent dye Cy5 and pooled with Cy3 labelled reference RNA [38] and then the pooled RNA was hybridized onto HEEBO microarrays. After hybridization and washing, the arrays were scanned on a fluorescent microscope scanner and the raw data files were stored in the Stanford Microarray Database [39].

**Figure 1:**
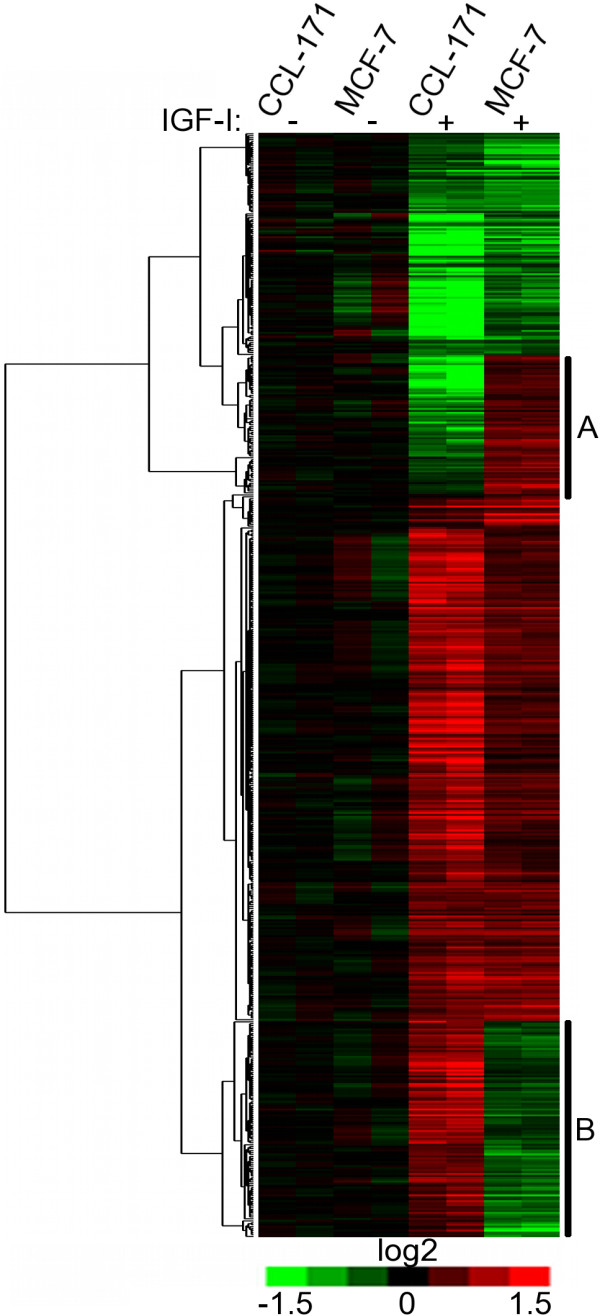
**The effects of insulin-like growth factor-1 (IGF-I) on gene expression in CCL-171 fibroblasts and MCF-7 tumour cells**. Unsupervised hierarchical clustering of genes deregulated in CCL-171 and MCF-7 cells upon IGF-I stimulation. The gene expression levels were normalized to the non-stimulated specimens as described. Genes are presented in rows and experiments in columns. The red and green colours provide information about up- or down-regulation, respectively. The intensity of the colour renders quantitative information about the change in expression level. IGF-I stimulation induces some common and some distinct effects on the gene expression profiles in different cell types. (A) Genes specifically up-regulated in MCF-7 cells involved in: epidermal growth factor and fibroblast growth factor signalling; protein metabolism and modification; nucleoside, nucleotide and nucleic acid metabolism. (B) Genes specifically up-regulated in CCL-171 cells include transcription factors and transferases, in addition to genes involved in Wnt and TGF-β signalling.

In order to establish the system, we characterized the response of both cell types to IGF-I separately. In both cell types, we observed a remarkable change in the gene expression profile following IGF-I stimulation (Figure 1). As the interaction between IGF-I and stromal cells in the tumour microenvironment has not yet been studied, we first characterized the IGF-I-induced genes in CCL-171 cells. The most prominent change after stimulation was a greater than 1.5-fold induction (mean: 2.35, standard deviation: 0.45) in the expression level of 370 genes (Additional file 1). The fibroblast derived IGF-I signature contains TTK, NEK2, PBK, SPBC24, RACGAP1, CLASP1, HECTD3, RCC2, MAD2L1, CDCA8, PTTG1, BIRC5, PKMYT1, HCAP-G, CCNB1, CENPF, CDC20, CKS2, SPAG5, PLK1, BUB1B, CCNF, KIF11, CDC25C, DLG7, BRRN1 and CDCA5, genes that are known to be involved in proliferation, cell cycle and mitotic cell division.

In order to check, in an unbiased way, what features the members of the IGF-I induced signature share and to verify the significance of enrichment of a specific gene ontology, we used the GO TermFinder tool [40]. The analysis revealed that the fibroblast derived IGF-I signature is significantly enriched for genes involved in biological processes such as M phase, mitotic cell cycle, mitosis and cell cycle, with a *P *value equal to ≈ 1.03e-10 and cell division with *P *≈ 1.03e-8 (Table 1 and Additional file 2). In addition, among the 370 unique genes up-regulated by IGF-I we found genes that are involved in angiogenesis, the p53 pathway and integrin and Wnt signalling. The mRNA expression level of six soluble factors already recognized in cancer biology (POSTN, TNC, CSPG2, LOXL1, ATRN, FBS1) increases in response to IGF-I stimulation, suggesting that these factors play some role in stimulating tumour cell proliferation and metastasis.

**Table 1:** Gene ontology terms for genes up-regulated in CCL-171 cells by insulin-like growth factor (IGF-I).

Gene ontology term	Cluster frequency	Gene frequency in background	Corrected *P*-value	FDR	False positives
M phase	42 out of 325 genes, 12.9%	67 out of 2133 genes, 3.1%	1.84E-16	0.0%	0.0

Cell cycle phase	45 out of 325 genes, 13.8%	82 out of 2133 genes, 3.8%	1.46E-14	0.0%	0.0

Cell division	38 out of 325 genes, 11.7%	69 out of 2133 genes, 3.2%	5.07E-12	0.0%	0.0

M phase of mitotic cell cycle	32 out of 325 genes, 9.8%	52 out of 2133 genes, 2.4%	1.06E-11	0.0%	0.0

Cell cycle process	50 out of 325 genes, 15.4%	113 out of 2133 genes, 5.3%	2.13E-11	0.0%	0.0

Nuclear division	31 out of 325 genes, 9.5%	51 out of 2133 genes, 2.4%	4.65E-11	0.0%	0.0

Mitosis	31 out of 325 genes, 9.5%	51 out of 2133 genes, 2.4%	4.65E-11	0.0%	0.0

Cell cycle	57 out of 325 genes, 17.5%	143 out of 2133 genes, 6.7%	7.11E-11	0.0%	0.0

Organelle fission	31 out of 325 genes, 9.5%	52 out of 2133 genes, 2.4%	9.95E-11	0.0%	0.0

Mitotic cell cycle	39 out of 325 genes, 12.0%	86 out of 2133 genes, 4.0%	7.94E-09	0.0%	0.0

Organelle organization	67 out of 325 genes, 20.6%	217 out of 2133 genes, 10.2%	4.21E-07	0.0%	0.0

Cellular component organization	91 out of 325 genes, 28.0%	358 out of 2133 genes, 16.8%	1.78E-05	0.0%	0.0

Microtubule-based process	21 out of 325 genes, 6.5%	40 out of 2133 genes, 1.9%	2.56E-05	0.0%	0.0

Regulation of mitotic cell cycle	17 out of 325 genes, 5.2%	31 out of 2133 genes, 1.5%	2.40E-04	0.0%	0.0

Spindle organization	10 out of 325 genes, 3.1%	14 out of 2133 genes, 0.7%	2.70E-03	0.4%	0.1

Microtubule-based Movement	10 out of 325 genes, 3.1%	15 out of 2133 genes, 0.7%	0.01	0.6%	0.1

Mitotic cell cycle checkpoint	9 out of 325 genes, 2.8%	13 out of 2133 genes, 0.6%	0.01	0.6%	0.1

Regulation of cell cycle	27 out of 325 genes, 8.3%	79 out of 2133 genes, 3.7%	0.01	0.6%	0.1

Biological regulation	152 out of 325 genes, 46.8%	785 out of 2133 genes, 36.8%	0.04	0.6%	0.1

Microtubule cytoskeleton organization	12 out of 325 genes, 3.7%	24 out of 2133 genes, 1.1%	0.05	0.7%	0.1

Detailed list of gene ontology terms specifically up-regulated in CCL-171 fibroblasts upon IGF-I stimulation in comparison to background file including all genes deregulated by IGF-I. Bonferroni corrected *p *values for of the output from GO::Termfinder for process ontology are listed.

FDR, false discovery rate.

We selected the MCF-7 cell line, the well-known representative of the luminal type breast cancer, to assess the global gene expression effects of IGF-I stimulation. IGF-I stimulation increased the mRNA expression level of numerous genes (such as BMP7, ID1, ID3, SRF and VEGF) that play a role in tumour biology as well as genes with ontologies assigned to protein metabolism (RPS6KA4, PSMC4, MAPK6, LMAN2L, RPL8, EIF5 and CEBPB), responses to a protein stimulus and to unfolded protein (HSP90AA1, HSPE1, HSPA1A, DNAJA1, HSPA4, HSP90AB1, HSPH1, and DNAJB1). In contrast to the upregulation of genes involved in the proliferation that we found in CCL-171 cells, the gene expression pattern in MCF-7 cells stimulated with IGF-I is not significantly associated with the cell cycle or proliferation.

After comparing these gene expression patterns, we hypothesized that the mesenchymal stromal cells and malignant epithelial cells exhibit distinct gene expression changes in response to IGF-I stimulation. In order to test this hypothesis, we compared the gene expression profiles of CCL-171 and MCF-7 cells with, and without, IGF-I stimulation. Within each cell type, we subtracted the expression profile of unstimulated cells from IGF-I stimulated cells and then filtered and merged the profiles and performed a hierarchical clustering of the genes. The results were visualized with a heat map using TreeView software [41], which showed that the IGF-I stimulus induced some common and some distinct effects on gene expression in the two cell types (Figure 1). This is easily explained when we consider the differences in the distinct default gene expression profiles of the two cell types, including the well-known markers for epithelial and mesenchymal cells (Additional file 3). The two gene clusters with discordant gene expression changes (Figure 1) were examined with the GO TermFinder tool. Genes that are up-regulated in CCL-171 and down-regulated in MCF-7 cells belong to the following ontologies: Wnt and TGF-β signalling and nucleic acid binding (transcription factors and transferases). Genes that are up-regulated in MCF-7 and down-regulated in CCL-171 cells are involved in protein metabolism and modification, as well as nucleoside, nucleotide and nucleic acid metabolism. This list also contains genes involved in epidermal growth factor and fibroblast growth factor signalling.

Thus, we concluded that, when epithelial and mesenchymal cells are exposed to IGF-I, they show some concordant and some discordant gene expression changes. Based on these observations, and given that the role of IGF-I in the stromal compartment is not yet well characterized, we further focused on the IGF-I response in stromal cells.

In order to characterize the gene expression programme induced in fibroblasts upon IGF-I stimulation, we extended our survey to primary breast fibroblasts. Rinn *et al. *[42] reported that fibroblasts from different body sites have unique default gene expression profiles, which led us to believe that distinct fibroblasts may respond differently to stimulation with IGF-I. Therefore, we felt that it would be important to analyse primary breast fibroblasts from breast cancer patients in order to examine the role that these stromal cells play in breast cancer.

**Figure 2:**
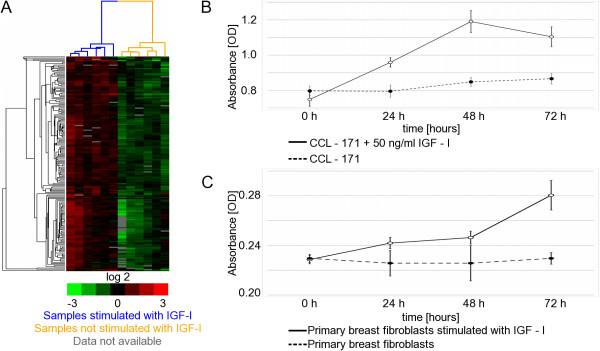
**Effects of insulin-like growth-1 (IGF-I) stimulation on primary breast fibroblasts and CCL-171 fibroblasts**. (A) Unsupervised hierarchical clustering of genes differentially expressed in fibroblasts upon IGF-stimulation. Unsupervised hierarchical clustering of genes differentially expressed between IGF-I stimulated and non-stimulated primary breast fibroblasts as discovered by SAM (genes with a false discovery rate ≤ 0.05% are represented). Grey fields indicate missing expression values. The colour of dendrogram branches renders information about sample stimulation; yellow = not stimulated and blue = stimulated with IGF-I (50 ng/mL). (B) IGF-I induced proliferation of CCL-171 cells. Cell proliferation assay based on absorbance measurement of WST-1. Formazan absorbance correlates to the cell number. Average absolute absorbance of replicates of CCL-171 cells stimulated with 50 ng/mL IGF-I in comparison to non-stimulated cells at different time points. Points represent the average of six replicates per condition and correspond to the cell number. The vertical error bars denote the standard deviation. Stimulation of CCL-171 cells with IGF-I induces significant, constant cell growth after 24, 48 and 72 h. (C) IGF-I induced proliferation of primary breast fibroblasts. Cell proliferation assay based on absorbance measurement of WST-1. Points represent the average absolute absorbance of a minimum of eight replicates of six primary fibroblasts (carcinoma associated fibroblasts and normal fibroblasts) after 24, 48 and 72 h. Error bars correspond to the magnitude of the standard deviation. Stimulation of primary breast fibroblasts with IGF-I induces significant, constant cell growth.

After obtaining informed consent from three patients with oestrogen and progesterone receptor positive and HER-2/neu negative invasive ductal adenocarcinoma of the breast, tissue specimens were obtained during breast tumour excision. An experienced breast pathologist distinguished tumour tissue from adjacent normal tissue. Carcinoma associated fibroblasts (CAF) and normal fibroblasts obtained from normal breast tissue of the same patient were cultured separately. The desired purity of the cell culture was obtained by serial passaging and separation with magnetic beads targeting fibroblast-specific antigens. Both cell types, CAF and normal fibroblasts, were stimulated with IGF-I and gene expression profiles were observed. We confirmed that the profiled cells were, indeed, mesenchymal fibroblasts because they showed an elevated expression of fibroblast markers, such as fibronectin 1 (FN1) and cadherin 2 (CDH2), and lacked E-cadherin (CDH1) expression (Additional file 4). The expression level of these specific markers did not change upon IGF-I stimulation (data not shown). All of the primary fibroblasts had a slightly higher IGF-IR mRNA expression level (mean: 1.6-fold; standard deviation: 0.24) compared to reference mRNA isolated from a pool of 11 cell lines [38], indicating that they might be responsive to IGF-I stimulation. The IGF-IR mRNA expression level decreased after IGF-I stimulation (mean: 0.6-fold; standard deviation: 0.09). In order to systematically identify significant gene expression changes upon IGF-I stimulation in primary cells, we applied a two-class significance analysis of microarray (SAM) data [43]. One class was formed by fibroblasts starved in low serum medium and the other class consisted of the same cells stimulated with IGF-I. SAM analysis revealed 208 gene IDs up-regulated and 300 gene IDs significantly repressed in stimulated cells (false discovery rate [FDR] ≤ 0.05%, Additional file 5). The 208 up-regulated genes were used to create the breast fibroblast derived IGF-I signature (Figure 2A). By comparing the gene function with the GO Termfinder, we observed that the genes up-regulated by IGF-I in primary breast fibroblasts (Additional file 5) share similar features to those up-regulated in IGF-I-stimulated CCL-171 cells (Additional file 1), suggesting that they are involved in the same processes (proliferation, cell cycle and mitosis - Additional file 6, Table 2). Contrary to our expectations, we did not find any significant differences in the response to IGF-I between CAF and normal fibroblasts.

**Table 2:** Gene ontology terms for genes up-regulated in breast fibroblasts by insulin-like growth factor-1 (IGF-I).

Gene Ontology term	Cluster frequency	Gene frequency in background	Corrected *P*-value	FDR	False positives
M phase	36 out of 186 genes, 19.4%	175 out of 8918 genes, 2.0%	2.00E-23	0%	0.00

Cell cycle phase	37 out of 186 genes, 19.9%	223 out of 8918 genes, 2.5%	1.18E-20	0%	0.00

Cell cycle process	41 out of 186 genes, 22.0%	314 out of 8918 genes, 3.5%	4.69E-19	0%	0.00

Nuclear division	28 out of 186 genes, 15.1%	128 out of 8918 genes, 1.4%	1.64E-18	0%	0.00

Mitosis	28 out of 186 genes, 15.1%	128 out of 8918 genes, 1.4%	1.64E-18	0%	0.00

Cell cycle	46 out of 186 genes, 24.7%	425 out of 8918 genes, 4.8%	2.85E-18	0%	0.00

M phase of mitotic cell cycle	28 out of 186 genes, 15.1%	131 out of 8918 genes, 1.5%	3.21E-18	0%	0.00

Organelle fission	28 out of 186 genes, 15.1%	133 out of 8918 genes, 1.5%	4.99E-18	0%	0.00

Mitotic cell cycle	33 out of 186 genes, 17.7%	229 out of 8918 genes, 2.6%	3.30E-16	0%	0.00

Cell division	28 out of 186 genes, 15.1%	164 out of 8918 genes, 1.8%	1.88E-15	0%	0.00

Microtubule-based process	24 out of 186 genes, 12.9%	130 out of 8918 genes, 1.5%	8.42E-14	0%	0.00

Microtubule-based movement	13 out of 186 genes, 7.0%	47 out of 8918 genes, 0.5%	3.61E-09	0%	0.00

Spindle organization	10 out of 186 genes, 5.4%	27 out of 8918 genes, 0.3%	3.73E-08	0%	0.00

Cytoskeleton-dependent intracellular transport	13 out of 186 genes, 7.0%	56 out of 8918 genes, 0.6%	4.11E-08	0%	0.00

Organelle organization	44 out of 186 genes, 23.7%	737 out of 8918 genes, 8.3%	6.24E-08	0%	0.00

Chromosome segregation	10 out of 186 genes, 5.4%	41 out of 8918 genes, 0.5%	3.83E-06	0%	0.00

Microtubule cytoskeleton organization	12 out of 186 genes, 6.5%	70 out of 8918 genes, 0.8%	8.84E-06	0%	0.00

Phosphoinositide-mediated signalling	8 out of 186 genes, 4.3%	27 out of 8918 genes, 0.3%	2.32E-05	0%	0.00

Mmitotic sister chromatid segregation	7 out of 186 genes, 3.8%	22 out of 8918 genes, 0.2%	9.41E-05	0%	0.00

Sister chromatid segregation	7 out of 186 genes, 3.8%	22 out of 8918 genes, 0.2%	9.41E-05	0%	0.00

Cellular component organization	50 out of 186 genes, 26.9%	1187 out of 8918 genes, 13.3%	3.40E-04	0%	0.00

Regulation of mitotic cell cycle	10 out of 186 genes, 5.4%	72 out of 8918 genes, 0.8%	1.03E-03	0%	0.00

Second-messenger-mediated signalling	8 out of 186 genes, 4.3%	44 out of 8918 genes, 0.5%	1.36E-03	0%	0.00

Regulation of cell cycle	16 out of 186 genes, 8.6%	197 out of 8918 genes, 2.2%	1.68E-03	0%	0.00

Cytoskeleton organization	16 out of 186 genes, 8.6%	198 out of 8918 genes, 2.2%	1.80E-03	0%	0.00

Protein polymerization	6 out of 186 genes, 3.2%	23 out of 8918 genes, 0.3%	2.63E-03	0%	0.00

Positive regulation of mitosis	4 out of 186 genes, 2.2%	7 out of 8918 genes, 0.1%	2.73E-03	0%	0.00

Amino acid biosynthetic process	6 out of 186 genes, 3.2%	25 out of 8918 genes, 0.3%	4.45E-03	0%	0.00

Chromosome localization	4 out of 186 genes, 2.2%	8 out of 8918 genes, 0.1%	0.01	0%	0.00

Establishment of chromosome localization	4 out of 186 genes, 2.2%	8 out of 8918 genes, 0.1%	0.01	0%	0.00

Cell cycle checkpoint	7 out of 186 genes, 3.8%	47 out of 8918 genes, 0.5%	0.02	0%	0.04

Establishment of localization in cell	24 out of 186 genes, 12.9%	472 out of 8918 genes, 5.3%	0.02	0%	0.04

Cellular localization	25 out of 186 genes, 13.4%	507 out of 8918 genes, 5.7%	0.03	0%	0.04

DNA metabolic process	17 out of 186 genes, 9.1%	275 out of 8918 genes, 3.1%	0.03	0%	0.06

Amine biosynthetic process	6 out of 186 genes, 3.2%	35 out of 8918 genes, 0.4%	0.03	0%	0.08

Serine family amino acid biosynthetic process	3 out of 186 genes, 1.6%	5 out of 8918 genes, 0.1%	0.04	0%	0.08

Detailed information and *P *values for single biological processes up-regulated by IGF-I in primary breast fibroblasts in comparison to background file including all genes used for significant analysis of mcroarray analysis as revealed by gene ontology term finder tool with a Bonferoni corrected *P *value higher than 0.05.

FDR, false discovery rate.

Taken together, primary fibroblasts coming from breast cancer and the normal breast, as well as CCL-171 fibroblasts, respond to IGF-I stimulation and display up-regulation of similar gene signatures involved in cell proliferation and mitotic cell division.

In order to verify that the gene expression profile is reflected by a phenotypic alteration upon IGF-I stimulation, we examined the proliferation rate of the fibroblasts. The cells were seeded and starved in low serum medium for 48 h in order to exclude any effects of fetal bovine serum (FBS) from regular cell growth culture conditions. The cells were then stimulated with IGF-I, and the cell proliferation was assessed with a colourimetric method using WST-1. Primary breast fibroblasts (Figure 2C), both normal and CAF, grew significantly faster when stimulated with IGF-I rather than unstimulated cells (*P *< 0.0001 for 24, 48 and 72 h, *t*-test, two-sided; *P *< 0.0001, analysis of variance [ANOVA]). A similar response to IGF-I stimulation was observed in CCL-171 fibroblasts as presented in Figure 2B (*P *< 0.0001 for 24, 48 and 72 h, *t*-test, two-sided; *P *< 0.001, ANOVA).

Taken together, these suggest that primary fibroblasts stimulated with IGF-I induce expression of genes involved in cell proliferation and mitotic cell division.

### Relevance of the fibroblast derived IGF-I induced gene signature *in vivo*

**Figure 3:**
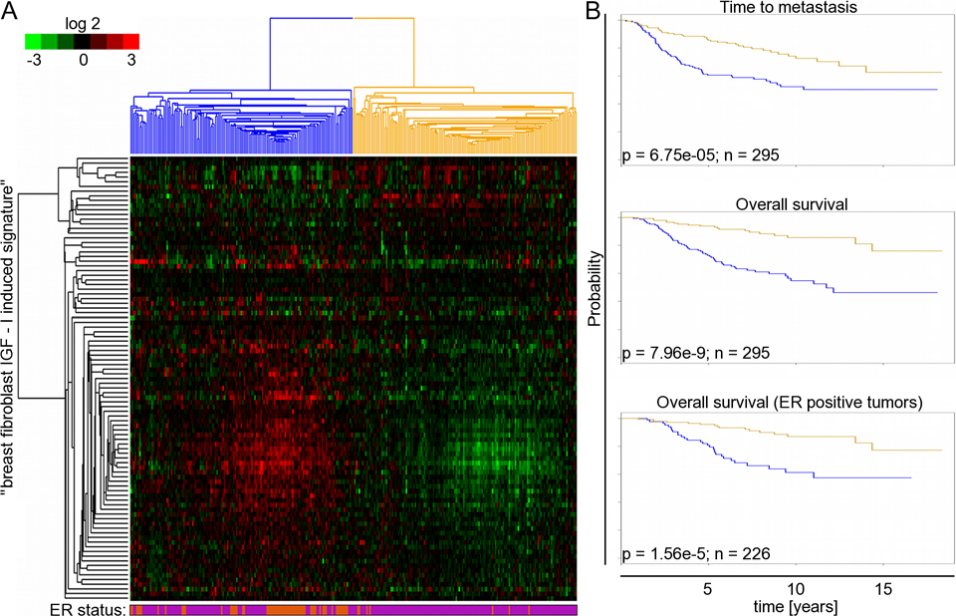
**Breast fibroblast derived IGF-I signature in early stage breast cancer**. (A) Unsupervised hierarchical clustering of breast fibroblast derived IGF-I signature in Netherlands Cancer institute dataset. The expression values of genes in the breast fibroblast derived IGF-I signature revealed by signficant analysis of microarray were extracted from a published expression study of 295 early stage breast cancers from the Netherlands Cancer Institute (NKI). Genes are presented in rows and experiments in columns. Breast fibroblast derived IGF-I signature stratifies early breast cancer patients (NKI) into two groups with high (blue) or low (yellow) expression levels of genes representing the signature. Horizontal bar below the figure represents positive (purple) or negative (orange) ER status. (B) Relationship of expression level of genes building breast fibroblast derived IGF-I signature with distant metastasis free and overall survival. Kaplan-Meier curves representing the clinical outcomes of tumors exhibiting high (blue curve) and low (yellow curve) expression levels of the IGF-I induced signature. The upper two figures represent all patients and the bottom figure shows only patients with oestrogen receptor positive breast tumours.

In order to verify the relevance of our *in vitro *experiments, we checked the expression of the breast fibroblast derived IGF-I signature in microarray data of early stage breast cancer biopsies from 295 patients from the Netherlands Cancer Institute (NKI), which are publicly available [44]. In the NKI dataset, the expression of the genes belonging to breast fibroblast derived IGF-I signature was coherent, providing a basis for segregation of the tumours into two groups. In one group the signature was up-regulated and in the other group the signature was down-regulated (left and right side of Figure 3A, respectively). As visualized with Kaplan-Meier plots (Figure 3B), the patients with early stage breast cancers with a high expression level of the breast fibroblast derived IGF-I signature had a significantly higher risk of developing metastasis than the patients with a low expression level (*P *= 6.75e-05, 52% versus 73% after 10 years, hazard ratio (HR): 2.24, 95% confidence interval [CI]: 1.5-3.4; top panel). In parallel, the overall survival rate was significantly lower for patients with up-regulation of the breast fibroblast derived IGF-I signature (*P *= 7.96e-09, 55% versus 86% after 10 years, HR: 4.03, CI: 2.4-6.8; middle panel). The same coordinated behaviour and segregation of tumours could also be observed in a set of advanced breast cancers from Norway/Stanford [45,46]. In a univariate analysis, patients with high expression levels of IGF-I induced genes had a significantly shorter disease-specific survival than patients with low expression levels (*P *= 0.0219, HR: 2.6, CI: 1.1-6.2, data not shown). In addition, as the classification of data based on hierarchical clustering was suggested to be unstable and codependent on many factors like presence of missing values [47], we validated the results using continuous scoring and stratified the patients of the NKI dataset based on a score derived from the average expression level of breast fibroblast derived IGF-I signature. In agreement with the results obtained by hierarchical clustering, the continuous scoring divided the early breast cancer patients (NKI dataset) [44] into two groups with significantly different outcomes (distant metastasis-free survival: *P *= 3.6e-07 and overall survival: *P *= 3.5e-09; Additional file 7).

On the molecular level, an interaction of IGF-I with the oestrogen receptor (ER) has been described. Therefore, we performed a multivariable analysis corrected for ER status (positive versus negative) in the early and advanced breast cancer datasets. The breast fibroblast derived IGF-I signature was able to stratify breast cancer patients into groups with significantly different outcomes even when corrected for ER status. The results of the multivariable analysis were significant (overall survival: *P *= 1.6e-09, time to metastasis: *P *= 2.2e-4 in the NKI dataset and disease specific survival in the Norway/Stanford dataset *P *= 8.6e-5, respectively). In both datasets, the combination of ER negative receptor status and up-regulation of the breast fibroblast derived IGF-I signature had the worst outcome. Additionally, in early stage breast cancer, the breast fibroblast derived IGF-I signature was able to segregate ER positive breast cancer patients into two groups with significantly different outcomes (*P *= 1.6e-5, Figure 3B, lowest panel). In summary, we found that genes induced in primary breast fibroblasts upon IGF-I stimulation predict the outcome of breast cancer patients. Furthermore, the expression signature distinguishes between patients with ER positive cancer who have significantly different prognoses.

### Correlation of the IGF-I induced gene signature with previously published prognostic gene expression signatures

**Figure 4:**
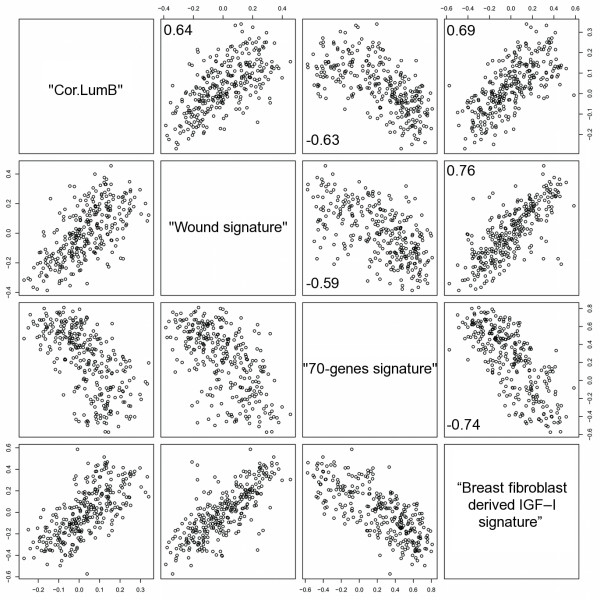
**Correlation of the breast fibroblast derived insulin-like growth factor-I (IGF-I) signature with previously reported prognosticators in breast cancer**. Correlation of the good-risk 70-genes signature centroid [49], the wound signature centroid [60], the basal type of breast cancer created by Soerlie [46] and the breast fibroblast IGF-I induced signature score in the Netherlands Cancer Institute dataset. Pairwise scatterplot-matrix of four gene signatures. Pearson correlations for the signature are shown in the corners of the plots.

As the breast fibroblast derived IGF-I signature is a prognostic marker in human breast cancer, we next sought to see if the signature might be related to other previously published gene-expression signatures, which were useful prognosticators in the NKI dataset. To this aim, we correlated the signatures based on their centroids, which represent the average expression values of all genes building the signature in a single tumour specimen, using the Pearson correlation test. First, we checked the correlation of the breast fibroblast derived IGF-I signature centroid to the wound signature centroid [48], which was created based on the response of fibroblasts to serum stimulation. The breast fibroblast derived IGF-I signature, as presented in Figure 4, was highly correlated to the wound signature (0.76). It was also moderately correlated (0.69) to basal type breast cancer [46]. Furthermore, the breast fibroblast derived IGF-I signature was highly reverse-correlated to the good-risk 70-genes signature (-0.74) [49]. The good-risk70-genes signature was created n order to predict freedom from metastasis in this same dataset. The detailed list of correlation values for all of the signatures may be found in Additional file 8.

### IGF induced genes are prognostic in lung cancer

**Figure 5:**
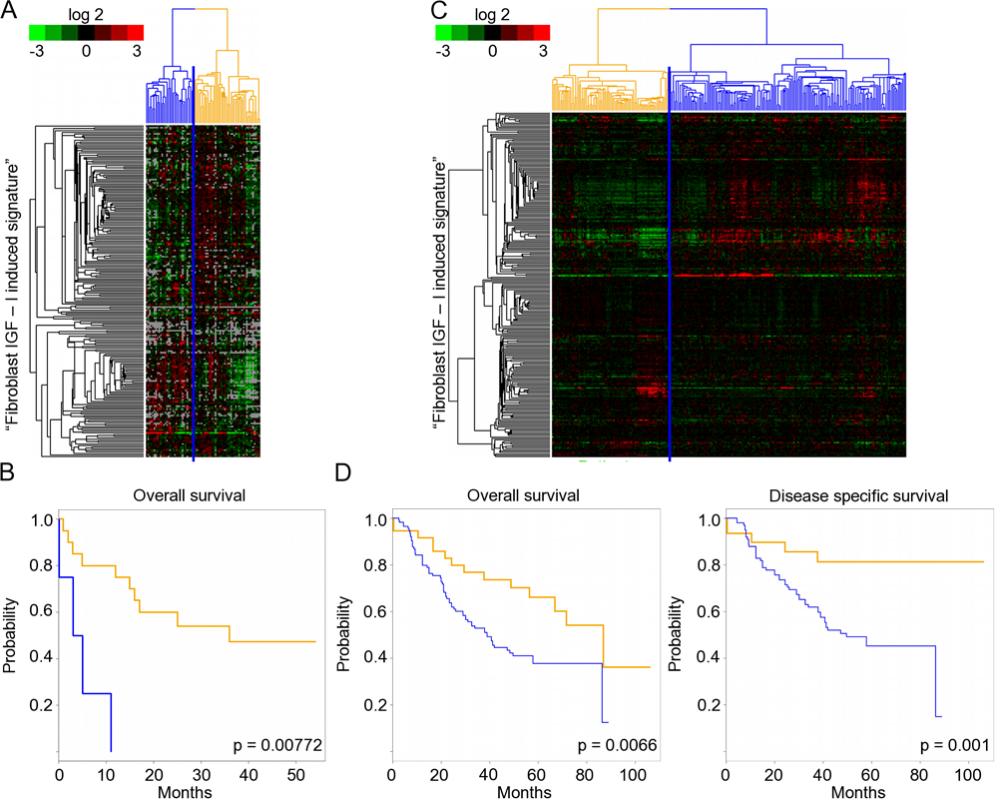
**Fibroblast derived insulin-like growth factor-I (IGF-I) signature divides lung cancer patients into two groups with significantly different outcome**. (A) Unsupervised hierarchical clustering of fibroblast derived IGF-I signature in Garber lung cancer dataset. The expression values of genes in the fibroblast derived IGF-I signature were extracted from a published expression study by Garber [50]. Genes are presented as rows and the experiments are presented as columns. Although some gene expression data are missing, the fibroblast derived IGF-I signature stratifies lung cancer patients into two groups with high (blue) or low (yellow) expression levels of genes representing the signature. (B) Relationship of expression level of genes building fibroblast derived IGF-I signature with overall survival in Garber data. Kaplan-Meier curves denoting the clinical outcomes of the indicated tumours exhibiting high (blue curve) and low (yellow curve) expression levels of the signature. (C) Unsupervised hierarchical clustering of fibroblast derived IGF-I signature in Bhattacharjee lung cancer dataset. The expression values of genes in the fibroblast derived IGF-I signature were extracted from a published expression study by Bhattacharjee [51]. Genes are presented as rows and the experiments are presented as columns. Fibroblast derived IGF-I signature stratifies adenocarcinoma patients into two groups with high (blue) or low (yellow) expression levels of genes representing the signature. (D) Relationship of expression level of genes building fibroblast derived IGF-I signature with overall survival and disease specific survival in Bhattacharjee dataset. Kaplan-Meier curves illustrating the clinical outcomes of the indicated tumours exhibiting high (blue curve) and low (yellow curve) expression levels of the signature.

Knowing that the gene expression signature derived from primary breast fibroblasts in response to IGF-I stimulation is relevant *in vivo*, and is a strong prognostic factor in human breast cancer, we investigated this finding to see if it could be generalized to other types of human cancer. We felt that this was likely because of the similarity between the IGF-I responses of primary breast fibroblasts and CCL-171 lung fibroblasts. We decided to check our hypothesis using the IGF-I derived signature from CCL-171 *in vitro *in lung cancer datasets. Global gene expression profiles of 67 human lung cancers were derived from 56 patients; 24 had survival data published by Garber *et al. *[50] (GEO: GSE3398). As shown in Figure 5A, in this dataset the expression of the lung fibroblast derived IGF-I gene signature was clear, even though the expression data for many genes was missing. This provided a basis for segregation of the tumours into two groups. The two groups were described as having the core part (proliferation associated genes) of the signature up-regulated or down-regulated (left and right side of Figure 5A, respectively). As visualized by Kaplan-Meier plots (Figure 5B), the patients with high expression levels of IGF-I induced genes had a significantly shorter overall survival (*P *= 0.008; *n *= 24, 60% versus 0% after 2 years, HR: 7.74, CI: 1.9-31.6). Thus, we concluded that the lung fibroblast derived IGF-I signature is a prognostic marker in lung cancer.

We then decided to validate our findings in a larger and better-annotated dataset published by Bhattacharjee [51], which contains microarray profiles of 203 tumours with clinical annotation for 125 of them. In line with our hypothesis, the expression of the lung fibroblast derived IGF-I signature was coherent, providing a basis for segregation of the tumours into two groups. The patients with a high expression level of the signature (right side of Figure 5C) had a significantly shorter disease-free survival (*P *= 0.001; 45% versus 82% after 5 years, HR: 3.7, CI: 1.5-9.4) and overall survival (*P *= 0.007; 38% versus 66% after 5 years, HR: 2.1, CI: 1.2-3.8) than the patients with low expression of the signature (left side of Figure 5C). Both Kaplan-Meier curves are shown in Figure 5D.

Taken together, these findings indicate that genes induced by IGF-I in human lung fibroblasts are helpful in predicting outcomes in human lung cancer. As the signature derived from breast and lung fibroblasts upon IGF-I stimulation is a prognostic marker for lung cancer, we suggest that the response of stromal fibroblasts to IGF-I might be a universal feature of cancer.

In order to further validate the general effect of IGF-I on fibroblasts, the ability of the breast and the lung fibroblast derived IGF-I signatures to be a prognostic factor in a non-site matching dataset was crosschecked. The breast fibroblast derived IGF-I signature was able to stratify patients with lung cancer (Bhattacharjee dataset) into two groups with significantly different rates of survival (overall survival with *P *= 0.043 and disease free survival with *P *= 0.022, data not shown). Also in this dataset, we validated the results obtained by hierarchical clustering using a continuous score and found a significant correlation (overall survival *P *= 0.005 and disease specific survival *P *= 0.001; Additional file 9).

The lung fibroblast derived IGF-I signature was also able to arrange breast carcinoma patients from the NKI dataset into two groups with significantly different times for metastasis free survival and overall survival (*P *= 7.9e-9 and *P *= 9.8e-6, respectively, data not shown).

N order to further cross validate the IGF-I signatures derived from fibroblasts of different origins, the correlation of the centroids for the signature obtained from lung fibroblasts were correlated to the signature derived from human primary breast fibroblasts (0.77; *P *- value < 2.2e-16, Additional file 10) in the NKI breast cancer dataset. The strong and significant correlation supports their similarity.

## Discussion

IGF-I has multiple effects on tumour initiation, development and progression and its effects on the cancer cells have been well described [13]. However, solid tumours do not consist only of malignant epithelial cells; rather, they form organ-like structures with a stroma consisting of fibroblasts, inflammatory cells and endothelial cells. Therefore, an endocrine or paracrine stimulus such as IGF-I might influence both the tumour cells and the stromal cells. The goal of this study was to characterize the effects of IGF-I on the cancer cells and the stromal fibroblasts in parallel. On the molecular level, cancer cells and fibroblasts show distinct response patterns to stimulation with IGF-I (Figure 1), including differential expression of genes involved in proliferation, protein metabolism and Wnt and TGF-β signalling. Focusing on the effect of IGF-I on MCF-7 cells, we observed alterations in protein metabolism. Similar changes in protein metabolism, including up-regulation of genes involved in transport and biosynthesis of amino acids, had already been reported previously in a global gene expression study of MCF-7 cells endogenously over-expressing IGF-I [52]. Additionally, we noted an up-regulation of VEGF in MCF-7 cells treated with IGF-I. VEGF is a known target gene for IGF signalling [52], with well-described implications in tumour progression and dissemination. Similar to our results, up-regulation of genes involved in metabolism and biosynthesis have been described in a comparable system of MCF-7 cells stimulated with exogenous IGF-I [53]. Apart from the similarities to the study by Creighton *et al. *[53], we also found discrepancies in the gene expression profile of proliferation-associated genes. The main reason might be because of the gene-wise standardization of the unstimulated samples applied in our setup which eliminated the inherent pattern of proliferation in MCF-7 cells.

With a focus on the stroma, there are studies showing that human dermal fibroblasts [35] and IMR90 fibroblasts [34] respond to IGF-I stimulation. Furthermore, it has been shown that primary breast fibroblasts over-express IGF-I and IGF-II (normal and malignant derived fibroblasts, respectively) [31,54] but none of these studies focused on the effects of IGF-I signalling on global gene expression. There was only one small study with first generation microarrays profiling the global gene expression effects of IGF-I stimulation in NIH-3T3 mouse fibroblasts, which showed an up-regulation of proliferation-associated genes [55]. To the best of our knowledge, we are the first to show microarray gene expression profiles of primary human breast fibroblasts in response to IGF-I. The gene expression changes induced by IGF-I in fibroblasts contained several soluble factors, such as POSTN, which was reported to be involved in bone metastasis formation and angiogenesis [56,57], TNC, which enhances tumour cell proliferation [58], as well as LOXL1, a member of lysyl oxidase family, similar to LOXL2, that might act on or in the vicinity of epithelial cells during tissue remodelling. LOXL2 has previously been reported to be involved in an invasiveness process [59] and specifically expressed by fibroblasts in tumour tissue [60]. The presence of these factors indicates that the IGF-I activated stroma enhances proliferation and the metastatic potential of the cancer cells.

That one single stimulus has both common and distinct effects on cells of different origins has been shown previously on the global gene expression scale for the response to oxygen deprivation under hypoxic conditions [61]. To the best of our knowledge, our experiments are the first to make a direct comparison of the effects of IGF-I on different cell types. Most interestingly, among the genes that were upregulated only in CCL-171 cells, and not in MCF-7 cells on IGF-I stimulation, did we observe many transcription factors (FUBP3, TEAD2, KLF16, SP3 and PIK3R3 involved in insulin receptor signalling pathway, MKNK2, SH3BP2 and CIT) all taking part in cell surface receptor linked signal transduction. Stromal cell specific genes among the IGF-I induced genes were of interest when we correlated this signature with *in vivo *data derived from whole tissue biopsies consisting of cancer cells and stromal cells. Signatures obtained from fibroblasts upon serum stimulation [60], as well as growth factor derived signatures, such as a TGF-β gene expression signature in mouse hepatocytes [62], are well-described prognosticators in human breast cancer. In our study, we confirmed the validity and robustness of IGF-I derived signatures from primary breast and lung fibroblasts in four different human solid cancer datasets. Genes induced in primary breast fibroblasts upon IGF-I stimulation are predictive of outcome in breast cancer patients. N addition, the signature allows for the stratification of ER positive breast cancer patients into two groups with significantly different prognoses. Prognostication in this heterogeneous patient population is important for clinical decisions about adjuvant therapies in patients with ER positive breast cancer.

The ability to derive prognostic information from cancer stroma has already been shown by Finak *et al. *[63]. The gene expression signature of stromal cells obtained by laser capture microdissection (LCM), the stroma derived prognostic predictor (SDPP), has been shown to be a prognostic marker in breast cancer. However, Finak *et al. *did not separate the different stromal components and, therefore, could not associate this signature to a specific cell type. In our study, we were able to specifically observe the effects of IGF-I on fibroblasts, which might be advantageous as targeted therapies are designed to specifically inhibit a signal at a particular cell type. Using laser capture microdissection, Roepman *et al. *managed to show that the genes expressed in the stroma are highly correlated with metastasis formation [64]. Specifically, they showed that 12% of the genes associated with lymph node metastasis in head-neck squamous cell carcinoma (HNSCC) are predominantly expressed in the stroma, 25% are tumour cell specific and the other genes are equally expressed in the tumour and the stroma. We speculate that the involvement of stroma-derived information might also be of importance in breast and in lung cancer. In our signatures, we found several of the genes that have been identified by Roepman *et al*. as being predominantly expressed in the stroma (ACTA1, TPM2, CDH2, COL5A1, COL5A2, HNRPL, TCF3). These segregated the patients into two groups with significantly different prognosis.

The IGF-I induced signatures in primary breast and in lung fibroblasts are similar to each other (Additional file 10) and to important, previously published signatures (Figure 4). The high reverse correlation of the IGF-I signature and the good-risk 70-genes signature supports the power of the IGF-I derived signature as a negative prognosticator in breast cancer. While the 'good-risk 70-genes signature' [49] was developed to predict freedom from metastasis in a top-down manner and validated in the same dataset of breast cancer patients from the NKI, the IGF-I induced signature is a marker for poor prognosis and is well connected to a defined *in vitro *biological system.

The IGF-I induced signature is also highly correlated to the wound signature [60], another strong prognostic signature in NKI dataset outperforming all known prognostic parameters so far. This is interesting, since a single growth factor, such as IGF-I, is able to induce a gene expression programme similar to the mix of undefined factors inherent in FBS. Using a fully defined stimulus in a concentration within the physiological range provides a simple and well-controlled *in vitro *model that enables specific experimental interventions to be made. Its effects can then be tested *in vivo*. Considering the notion by Sotirou [65] that proliferation is a main driver of the strong prognostic signatures such as the good-risk 70-genes signature and the wound signature facilitates speculation that IGF-I is one of the important factors responsible for the induction of proliferation. This does not exclude other, equally or more important, growth factors from inducing proliferation and up-regulation of proliferation associated genes.

We observed that both IGF-I signatures derived from lung and breast fibroblasts are exchangeable prognostic factors for the other cancer type, which allowed us to speculate that we could generalize this finding to other types of human solid cancer. The consistent response of fibroblasts (our data and [34,35,55]) to IGF-I might also help to explain the worse outcome of patients with elevated IGF-I levels in different cancer types [4-8], a finding that is not necessarily explained by the cancer cells themselves based on their IGF-receptor expression status on the cell surface. Specifically, since the correlation of the IGF-IR expression and patient outcome in human breast cancer is conflicting [66], the IGF-I induced gene expression signature showing the functional effects of IGF-I axis stimulation, which is correlated with the patients' clinical outcome, might be of interest when selecting patients who might benefit best from IGF-I blocking therapies.

IGF-I signalling is an emerging cancer drug target. *In vivo*, in mouse models, confirms that block IGF-I signalling demonstrate efficacy in inducing tumour regression and growth arrest [29] and sensitized cancer cells to conventional chemotherapeutic treatment and irradiation [67]. Exogenously added IGF binding protein I (IGFBP-1) inhibits IGF-I mediated growth of breast cancer cells [68,69]. Many other inhibitors of IGF signalling, applying different approaches [67], are currently under clinical investigation in phase I and II trials (reviewed in [29,70]). Some have already shown promising results, such as the phase II study on CP- 751, 851. This anti-insulin-like growth factor I receptor antibody, together with paclitaxel and carboplatin, was suggested to be safe and showed promising effectiveness in patients with non-small-cell lung cancer (NSCLC) showing the highest overall response rate of 78% in squamous cell carcinoma and 58% in adenocarcinomas [71]. Besides the monoclonal antibodies, there are small molecule inhibitors, such as XL228, that have blocking activity in the IGF1-R pathway and also in Src, fibroblast growth factor receptors (FGFR) and BCR-Abl pathways [72]. Although compounds that block IGF-I signalling demonstrate efficacy in inducing tumour regression and growth arrest *in vivo*, there is an emerging need to develop markers that predict a response to these therapies. We have tested the prognostic significance of our signature in patients with adenocarcinomas. In this group of patients, showing the lower response rate to IGF-I targeting therapies than squamous cell carcinomas [71], a better selection using a marker with predictive power would be especially beneficial. It might, therefore, be worthwhile to test whether or not the gene expression signatures developed and described here are useful predictive markers for IGF-I signalling blockade.

## Conclusions

The consistent and similar gene expression changes in human primary breast and lung fibroblasts suggest that the proliferative response to IGF-I is a general feature of stromal fibroblasts. Expression patterns of genes induced by IGF-I in primary breast and lung fibroblasts accurately predict outcomes in breast and in lung cancer patients. As IGF-I signalling is an emerging cancer drug target there is an emerging need to develop markers that predict a response to these therapies. Our IGF-I induced gene signatures derived from stromal fibroblasts might be promising predictors for the response to IGF-I targeted therapies.

## Methods

### Cell culture

Human primary fibroblasts CCL-171 and the human breast cancer cell line MCF-7 were obtained from American Type Culture Collection (ATTC, Atlanta, USA). Cells were propagated in Dulbecco's modified Eagle's medium (D-MEM, Invitrogen, Carlsbad, USA) supplemented with 10% heat inactivated FBS (Invitrogen), 4.5 g/lglucose, 4 mM L-glutamine and 100 U/ml penicillin and 100 μg/ml streptomycin (Gibco, Carlsbad, USA). Cells were maintained by regular passages when confluent. The study was approved by the Ethikkommission beider Basel, Switzerland (approval No. 271/05). Tumour and healthy tissue were obtained with consent from the patients who underwent surgery in University Hospital of Basel. For each patient, a sample of malignant tissue and a sample of side-matched healthy tissue were extracted by an experienced pathologist. The tissue was digested in a collagenase and RNase mix for 1 h and pressed through a 230 μm pore diameter sieve (Sigma Aldrich, St Louis, USA). The cells were cultured in a 1:1 v/v mixture of RPMI 1640 (Sigma Aldrich) and F12 Hamm (Gibco) medium supplemented with 12.5% FBS (Invitrogen), 2 mM Puryvat (Gibco), 4 mM L-glutamin (Gibco), 1 × Minimal Non-Essential Amino Acids (Gibco), 1 × RPMI 1640 Vitamins Solution (Sigma Aldrich), 100 U/ml penicillin and 100 μg/ml streptomycin (Gibco) and propagated until confluent. At this stage, cells were selected with anti-Fibroblast MicroBeads (Miltenyi Biotec, Gladbach, Germany) according to the manufacturer's instructions. All cells used in the experiments were kept in culture up to a maximum of 10 passages.

### IGF-I stimulation

For the experiment, 30,000 cells/cm^2 ^were seeded in 3 mL of 5% FBS D-MEM for CCL-171 cells and 5% FBS RPMI 1640/F12 mix for primary cells for 6 h, so that they would attach. The cells were extensively washed with phosphate buffered saline and starved for 48 h in fresh low-serum medium (0.2% FBS), D-MEM and RPMI 1640/F12 mix for CCL-171 and primary cells, respectively. The cells were starved n order to reduce the effects of any stimulation from regular cell culture medium. The medium was subsequently replaced by fresh low-serum D-MEM with or without 50 ng/ml of IGF-I (human recombinant in *Escherichia coli*; Sigma Aldrich). The cells were stimulated for 24 h and the RNA was harvested to test the effects of IGF-I on mRNA expression patterns.

### WST-1 proliferation assay

The proliferation reagent (Roche Diagnostics GmbH, Roche Applied Science, Basel, Switzerland) was used according to the manufacturer's instructions. In our setup, cells were plated in 96 well plates and starved for 48 h in low serum conditions. After, the cells were incubated in low-serum D-MEM with 50 ng/ml IGF-I over 24 h. n order to determine the cell numbers, the cells were stained with 10% WST-1 in low-serum D-MEM at 37°C, 5%CO_2 _for 2 h. The absorbance was measured with an ELISA reader at a wavelength of 450 nm. The proliferation rate of IGF-I stimulated primary breast fibroblasts and CCL-171 cells was compared to a respective reference samples not stimulated with IGF-I.

### RNA extraction and amplification

After aspirating the culture medium, the cell monolayer was washed once with phosphate buffered saline. The cells were lysed in a buffer containing guanidine isothiocyanate (RLT buffer, QIAGEN, CA, USA). The total RNA was extracted with the RNeasy kit (QIAGEN, CA, USA) according to the manufacturer's instructions. The RNA concentration was measured with a NanoDrop system spectrophotometer (ND-1000 Spectrophotometer Technologies, Wilmington, USA). The integrity of extracted RNA was checked by electrophoresis in a 1% agarose gel in MOPS buffer. For mRNA amplification, the Amino Allyl MasageAmp™ II aRNA Amplification Kit was used (Ambion, TX, USA). Amplification of mRNA out of 500 ng total RNA, the purification of cDNA, the *in vitro *transcription and the purification of aRNA were performed according to the manufacturer's instructions. Integrity and quantity of the amplified RNA was verified as described above.

### Gene expression analysis using HEEBO microarrays

For global gene expression analysis, we used HEEBO. The HEEBO microarrays consist of 44,544 70mer probes, which include: (a) constitutive exonic probes (30,718); (b) alternatively spliced/skipped exonic probes (8,441); (c) non-coding RNA probes (196); (d) BCR/TCR genic/regional probes (372); (e) other probes (843); and (f) controls. HEEBO microarrays were produced at the Stanford Functional Genomic Facility (Stanford, USA). Complete details regarding the clones on the arrays may be found at Stanford functional genomics facility website [73]. For microarray experiments, 8 μg amplified RNA (aRNA) were mixed with doping controls. Samples were vacuum dried, resolved in coupling buffer and labelled with Cy5 dye. Labelled samples were pooled with equal amounts of reverse coloured Cy3 labelled amplified reference RNA from Stratagene (Stratagene, CA, USA). The labelled aRNA was purified with AminoAllyl MasageAmp™ II aRNA Amplification Kit (Ambion) according tothe user manual and fragmented using fragmentation reagents (Ambion). The fragmented probe was added to a hybridization buffer containing Cot/PolyA/tRNA (0.05 μg/uL each), 0.3% SDS, 3.3 × SSC and supplemented with HEPES buffer. Following a denaturing step at 100°C, the probe was placed on the microarray for competitive hybridization. After 18 h, slides with hybridized probes were sequentially washed and immediately dried in an ozone free environment and scanned using an Axon Scanner 4100A (Axon Instruments, CA, USA). The gene expression profiles of primary fibroblasts, together with accompanying clinical data are available on SMD database papers' webpage [39]. In addition, the raw data have been deposited in NCBI's Gene Expression Omnibus [74] and are accessible through GEO Series accession number GSE18955 http://www.ncbi.nlm.nih.gov/geo/query/acc.cgi?acc=GSE18955.

### Data analysis and clustering

Microarray fluorescent image analysis was performed using the software Genepix Pro version 5.0 3.0.6.89 (Axon Instruments). Spots with obvious array artifacts or poor technical quality were manually removed from any further analysis. Raw data files were stored in the Stanford Microarray Database [39]. The data used for the paper are available at the accompanying website at Stanford Microarray Database [39]. Data were expressed as the log^2 ^ratio of fluorescence intensities of the sample and the reference for each element on the array. A sequential data filtering procedure was applied to include only measurements fulfilling our quality requirements (data with regression correlation bigger than 0.6 and Cy3 channel or Cy5 channel mean intensity over median background intensity bigger than 1.5). Genes that did not meet these criteria for at least 60% of the measurements across the experimental samples were excluded from further analysis. We rejected elements that did not have at least a 1.5-fold deviation from the mean in at least two samples. Data were evaluated by unsupervised hierarchical clustering [75] and displayed using Treeview software [41]. For the stimulation experiments, in order to emphasize the effect of IGF-I treatment, the results for each gene were standardized for each gene individually to the non-treated samples. In order to standardize them, we subtracted an average value of non-treated samples from each gene expression value for each cell type separately. This was performed in order to highlight those genes whose expression level changed upon treatment. Extraction of fibroblast gene signature and differentially expressed clusters was based on the correlation within the cluster nodes and, therefore, not randomly selected or based on an arbitrary cut- off.

In order to compare the gene expression profile of CCL-171 and MCF-7 cells in response to IGF-I, we merged the filtered, standardized gene expression profiles of both cell lines. We then manually excluded samples with a high standard deviation between the biological replicates and those missing gene expression data. Gene expression data for different clones representing one gene were averaged. A set of 566 unique genes was hierarchically clustered in an unsupervised manner [75] and displayed using Treeview software [41].

### SAM

For primary fibroblasts, two-class SAM was applied [43]. One class was formed by normal and carcinoma associated fibroblasts starved in low serum medium and the other by the same cells treated with IGF-I. In order to increase the sensitivity, we paired our samples.

### Human cancer datasets

A dataset containing gene expression patterns from advanced breast cancers was previously described by Sorlie *et al. *as Norway/Stanford dataset [45,46]. Expression measurements for each gene and array were mean centred. The list of 208 unique genes building breast fibroblast derived IGF-I signature was extracted from the Norway/Stanford dataset. In order to overcome possible overweighting of clones from Unigene clusters that were matched to more than one probe on the Sorlie array, expression values derived from probes matched to the same Unigene cluster were averaged. Only genes that had >80% data values present and tumour samples from patients having complete clinical data were used. The resulting dataset was subjected to average linkage hierarchical clustering [75] and displayed with Treeview [41].

Disease specific survival analysis was based on death from the disease and patients were censored at the last follow up. Patients who died from other causes were considered alive and not censored. Kaplan-Meier survival curves were compared using R package survival fitting a Cox proportional hazards regression model [76].

The dataset for early stage breast cancer contained 295 breast cancer specimens analysed on a 25,000 spot oligonucleotide array, as described previously [44]. In brief, patients were diagnosed and treated at the Netherlands Cancer Institute (NKI) for early stage breast cancer (stage I and II) between 1984 and 1995. The clinical data was updated in January 2005. The median follow-up for patients still alive is 12.3 years. Expression data from the NKI dataset were extracted as described above for the Norway/Stanford dataset. Distant metastases were analysed as a first event only (distant metastasis-free probability). Any patient who developed a local recurrence, axillary recurrence, contralateral breast cancer or a second primary cancer (except for non-melanoma skin cancer), was censored at that time and subsequent distant metastases were not analysed. This is based on the theoretical possibility that the locally recurrent or second primary cancers could be a source for distant metastases. An ipsilateral supra-clavicular recurrence was soon followed by a distant metastasis in all but one patient. Thus, an ipsilateral supra-clavicular recurrence was considered the first clinical evidence for metastatic disease for this analysis and patients were not censored at the time of ipsilateral supra-clavicular recurrence. Overall survival was analysed based on death from any cause and patients were censored at last follow up. Kaplan-Meier survival curves were fitted using a Cox proportional hazards regression model (R survival package) [76].

The dataset published by Garber and colleagues [50] contains global gene expression profiles for 67 human lung cancers derived from 56 patients with survival data for 24 patients. The dataset published by Bhattacharjee [51] contains mRNA expression levels of 12,600 transcript sequences in 186 lung tumor samples, including 139 adenocarcinomas resected from the lung. Of these, 125 samples were associated with clinical data (some patients in multiple runs). The Bhattacharjee dataset was obtained from the Broad Institute website [77] and Garber dataset from SMD publication webpage [39]. The list of 370 unique genes building fibroblast derived IGF-I signature was extracted from the Garber and Bhatacharjee datasets as described above for breast cancer datasets. Equally, the resulting dataset was subjected to average linkage hierarchical clustering [75] and displayed with Treeview [41]. Overall survival was analysed based on death from any cause and patients were censored at last follow up. Disease specific survival analysis was based on death from the disease and patients were censored at last follow up. Patients who died from other causes were considered alive and not censored. Kaplan-Meier survival curves were fitted using a Cox proportional hazards regression model (R package 'survival') [76].

### Centroid correlation

The method of calculating the centroid for each patient was previously described by Sorlie [45]. Briefly, the centroids for genes representing breast fibroblast derived IGF-I signature and fibroblast derived IGF-I signature, as well as other signatures, were calculated based on the NKI dataset. To test for similarities between the signatures, we checked the correlation between values of different centroids for one patient. The correlation was calculated using Pearson correlation coefficient with R software [76].

### Continuous scoring

The stratification of patients within the NKI and Bhattacharjee datasets was conducted according to the previously described methodology [60,61] based on a continuous score derived from the signatures. Briefly, the average expression level of each signature was calculated for each patient attributing a score. The patients were then divided into two groups separating them by the median value of the continuous scores. Kaplan-Meier survival curves for the two groups were plotted and the statistical significance was determined using a Cox proportional hazards model (R package 'survival') [76].

### GO::TermFinder analysis

GO::TermFinder takes a list of genes as input, and determines whether those genes have any gene ontology (GO) terms overrepresented in their combined set of annotations compared to what would be expected by chance from a randomly selected group of genes from the background population of all genes [39,40]. In our analysis, we used the full gene lists from parental heat maps as a file to calculate the frequency of particular annotations in a background file and the gene lists from specific clusters coming from same heat map to calculate the frequency of particular annotations in the defined cluster. For a SAM-derived signature, we used a gene list that was an input file for SAM analysis.

### General statistic methods

Normally distributed data were compared using a Student's *t*-test. When the multiple comparisons were necessary, the data were analysed with ANOVA. Differences were considered as statistically significant when *P *< 0.05. *T*-tests and ANOVA analysis were done using R software (R package 'stats') [76].

## Competing interests

The authors declare that they have no competing interests.

## Authors' contributions

MR and MB designed the research, analysed and interpreted the data. MR and BV performed the research. MB, RZ recruited the patients and analysed clinical data. All authors have been involved in drafting the manuscript and revising it critically for important intellectual content and have given final approval of the version to be published.

## Pre-publication history

The pre-publication history for this paper can be accessed here:

http://www.biomedcentral.com/1741-7015/8/1/prepub

## Supplementary Material

Additional file 1 **Table S1**. List of genes building the fibroblast derived insulin-like growth factor-1 (IGF-I) signature. Click here for file

Additional file 2 **Figure S2**. Graphical visualization of the output from GO::Termfinder for biological process ontology. GOgraph layout that includes the significant GO nodes up-regulated in CCL-171 cells, derived from 325 clones compared to a background of 2133 clones. The colour of the nodes is an indication of their Bonferroni corrected *P*-value (orange <= 1e-10; yellow 1e-10 to 1e-8; green 1e-8 to 1e-6; cyan 1e-6 to 1e-4; blue 1e-4 to 1e-2; tan > 0.01). Click here for file

Additional file 3 **Figure S1**. Distinct default gene expression profiles of human lung fibroblasts and breast tumour cells. Genes are presented in rows and experiments in columns. Both cell types demonstrate a clearly distinct default gene expression profile, typical for epithelial and mesenchymal cells. Gene markers typical for mesenchymal (FN1, CDH2, VIM) and epithelial/tumour cells (CDH1, TPD52, BMP-7) are marked. Additionally, examples of proliferation associated genes up-regulated in MCF-7 cells by default are shown. Click here for file

Additional file 4 **Figure S3**. Box-and-whisker plot illustrating the average expression level of fibronectin (FN1), N-cadherin (CDH2) and E-cadherin (CDH1) in primary fibroblasts. Insulin-like growth factor (IGF-I) does not affect the expression level of mesenchymal and epithelial markers in primary breast fibroblasts (data not shown). Click here for file

Additional file 5 **Table S2**. List of genes up-regulated (the breast fibroblast derived insulin-like growth factor-1 [IGF-I] signature) and down-regulated in primary breast fibroblasts upon IGF-I stimulation. Click here for file

Additional file 6 **Figure S4**. Graphical visualization of the output from GO::Termfinder for biological process ontology. GOgraph layout that includes the significant GO nodes up-regulated in primary breast fibroblasts, derived from 186 clones compared to a background of 8918 clones. The colour of the nodes is an indication of their Bonferroni corrected *P*-value (orange <= 1e-10; yellow 1e-10 to 1e-8; green 1e-8 to 1e-6; cyan 1e-6 to 1e-4; blue 1e-4 to 1e-2; tan > 0.01). Click here for file

Additional file 7 **Figure S6**. Relationship of expression level of breast fibroblast derived insulin-like growth factor-1 (IGF-I) signature with distant metastasis free and overall survival applying continuous scoring. A. Continuous score based on average expression level of the signature in Netherlands Cancer Institute (NKI) patients. Colours correspond to score below (yellow) or above (blue) the median (red line). Overall (B) and metastasis free survival (C) analysis using a continuous score resulting from breast fibroblast derived IGF-I signature in early stage breast cancer patients from the NKI. Click here for file

Additional file 8 **Table S3**. The detailed list of correlation values of breast fibroblast derived insulin-like growth factor-1 (IGF-I) signature to the previously published signatures and fibroblast derived IGF-I signature. Click here for file

Additional file 9 **Figure S7**. Relationship of expression level of breast fibroblast derived insulin-like growth factor-1 (IGF-I) signature with overall survival and disease specific survival applying continuous scoring. A. Continuous score based on average expression level of the signature in Bhattacharjee dataset patients. Colours correspond to score below (yellow) or above (blue) the median (red line). Overall (B) and disease specific survival (C) analysis using a continuous score resulting from breast fibroblast derived IGF-I signature in Bhattacharjee dataset patients. Click here for file

Additional file 10 **Figure S5**. Correlation of the fibroblast derived insulin-like growth factor-1 (IGF-I) signature and the breast fibroblast IGF-I induced signature centroids in the Netherlands Cancer Institute dataset. Pearson correlations for the signature and the *P *value are shown in the lower right part of the plot. Click here for file
