# Supplementary material for: IGF-I induced genes in stromal fibroblasts predict the clinical outcome of breast and lung cancer patients
Source: BMC Med. 2010 Jan 5;8:1. doi: 10.1186/1741-7015-8-1 (PMC2823652; doi:10.1186/1741-7015-8-1)
Supplement: Additional file 1 — Table S1. List of genes building the fibroblast derived insulin-like growth factor-1 (IGF-I) signature. [file 1741-7015-8-1-S1.PDF]

**Gene symbol**	**Gene name**	**GenBank Accession Numer**
MMP1	matrix metallopeptidase 1 (interstitial collagenase)	
COL15A1	collagen, type XV, alpha 1	NM_001855
COPG	coatomer protein complex, subunit gamma	
RPL29	ribosomal protein L29	NM_000992
LOXL1	lysyl oxidase-like 1	
EDG1	endothelial differentiation, sphingolipid G-protein-coupled receptor, 1	
TRAM2	translocation associated membrane protein 2	
FAT	FAT tumor suppressor homolog 1 (Drosophila)	
COL4A5	collagen, type IV, alpha 5 (Alport syndrome)	NM_033380
UHRF1	ubiquitin-like, containing PHD and RING finger domains, 1	NM_013282
MLLT11	myeloid/lymphoid or mixed-lineage leukemia (trithorax homolog, Drosophila); translocated to, 11	
RAB6IP1	RAB6 interacting protein 1	
MAML1	mastermind-like 1 (Drosophila)	
PEG10	paternally expressed 10	
LOC389036	actin-like protein (ACT) gene	XM_371558
ACTB	actin, beta	NM_001101
TMEM131	transmembrane protein 131	
VASP	vasodilator-stimulated phosphoprotein	
PHF21A	PHD finger protein 21A	NM_016621
RNPS1	RNA binding protein S1, serine-rich domain	NM_006711
TP53	tumor protein p53 (Li-Fraumeni syndrome)	
ISGF3G	interferon-stimulated transcription factor 3, gamma 48kDa	
AOF1	amine oxidase (flavin containing) domain 1	
ATP2B4	ATPase, Ca++ transporting, plasma membrane 4	NM_001684
XPR1	xenotropic and polytropic retrovirus receptor	NM_004736
CDH2	cadherin 2, type 1, N-cadherin (neuronal)	
TES	testis derived transcript (3 LIM domains)	NM_015641
TULP4	tubby like protein 4	NM_020245
TRNS2	tRNA	NC_001807
H1F0	H1 histone family, member 0	
SFPQ	splicing factor proline/glutamine-rich (polypyrimidine tract binding protein associated)	
SYT1	synaptotagmin I	
COL5A2	collagen, type V, alpha 2	
COL5A1	collagen, type V, alpha 1	
JMJD1B	jumonji domain containing 1B	
USP10	ubiquitin specific peptidase 10	
EHMT1	euchromatic histone-lysine N-methyltransferase 1	NM_024757
FLJ20397	hypothetical protein FLJ20397	NM_017802
R3HDM1	R3H domain containing 1	
MPHOSPH1	M-phase phosphoprotein 1	NM_016195
NT5E	5'-nucleotidase, ecto (CD73)	
RPL6	ribosomal protein L6	
SMC4L1	SMC4 structural maintenance of chromosomes 4-like 1 (yeast)	NM_005496
TMEM100	transmembrane protein 100	
CSPG2	chondroitin sulfate proteoglycan 2 (versican)	
FLJ20364	hypothetical protein FLJ20364	
MAD2L1	MAD2 mitotic arrest deficient-like 1 (yeast)	NM_002358
TOP2A	topoisomerase (DNA) II alpha 170kDa	
ASPM	asp (abnormal spindle)-like, microcephaly associated (Drosophila)	
SORD	sorbitol dehydrogenase	
SRRM1	serine/arginine repetitive matrix 1	NM_005839
ARL6IP	ADP-ribosylation factor-like 6 interacting protein	
KPNA4	karyopherin alpha 4 (importin alpha 3)	
GAPVD1	GTPase activating protein and VPS9 domains 1	
SOX11	SRY (sex determining region Y)-box 11	NM_003108
SLC23A2	solute carrier family 23 (nucleobase transporters), member 2	NM_203327
ARID1A	AT rich interactive domain 1A (SWI- like)	
SFRS6	splicing factor, arginine/serine-rich 6	NM_006275
DNAJC14	DnaJ (Hsp40) homolog, subfamily C, member 14	
STK38	serine/threonine kinase 38	
CCND2	cyclin D2	NM_001759
ZNF608	zinc finger protein 608	
LMAN2L	lectin, mannose-binding 2-like	
RBM3	RNA binding motif (RNP1, RRM) protein 3	
CHFR	checkpoint with forkhead and ring finger domains	
SGOL2	shugoshin-like 2 (S. pombe)	
RPH3A	rabphilin 3A homolog (mouse)	NM_014954
NUSAP1	nucleolar and spindle associated protein 1	
LOC112714	similar to alpha tubulin	
CEP110	centrosomal protein 110kDa	
AKR1C1	aldo-keto reductase family 1, member C1 (dihydrodiol dehydrogenase 1; 20-alpha (3-alpha)-hydroxysteroid dehydrogenase)	
SLD5	SLD5 homolog	
KIF11	kinesin family member 11	NM_004523
CASC5	cancer susceptibility candidate 5	
POSTN	periostin, osteoblast specific factor	
KIAA0179	KIAA0179	D80001
SMAP1L	stromal membrane-associated protein 1-like	

EIF2AK3	eukaryotic translation initiation factor 2-alpha kinase 3	
FEM1A	fem-1 homolog a (C.elegans)	
NUP205	nucleoporin 205kDa	
DKFZp434H1419	hypothetical protein DKFZp434H1419	AK125369
COX7B	cytochrome c oxidase subunit VIIb	
CDCA1	cell division cycle associated 1	
KPNA2	karyopherin alpha 2 (RAG cohort 1, importin alpha 1)	NM_002266
LUZP5	leucine zipper protein 5	
BAG3	BCL2-associated athanogene 3	
HCAP-G	chromosome condensation protein G	
KIF15	kinesin family member 15	
PBK	PDZ binding kinase	
FLJ20105	FLJ20105 protein	
RPS24	ribosomal protein S24	
ZWILCH	Zwilch, kinetochore associated, homolog (Drosophila)	
PFTK1	PFTAIRE protein kinase 1	
KIF20A	kinesin family member 20A	NM_005733
BUB1	BUB1 budding uninhibited by benzimidazoles 1 homolog (yeast)	
SALL1	sal-like 1 (Drosophila)	NM_002968
PRR3	proline rich 3	
ABCC1	ATP-binding cassette, sub-family C (CFTR/MRP), member 1	
KIAA0746	KIAA0746 protein	
ZNF561	zinc finger protein 561	
ATRN	attractin	
FAM21B	family with sequence similarity 21, member B	AK125147
C14orf65	chromosome 14 open reading frame 65	AK123840
DLG7	discs, large homolog 7 (Drosophila)	
BCAR3	breast cancer anti-estrogen resistance 3	
POLA	polymerase (DNA directed), alpha	
TUBB	tubulin, beta	NM_178014
TNC	tenascin C (hexabrachion)	
MGC3121	hypothetical protein MGC3121	
NOTCH1	Notch homolog 1, translocation-associated (Drosophila)	NM_017617
THOC4	THO complex 4	
PDIA4	protein disulfide isomerase family A, member 4	
TRIP13	thyroid hormone receptor interactor 13	
KIF4A	kinesin family member 4A	NM_012310
C20orf129	chromosome 20 open reading frame 129	
WHSC1	Wolf-Hirschhorn syndrome candidate 1	NM_014919
BUB1B	BUB1 budding uninhibited by benzimidazoles 1 homolog beta (yeast)	
BRRN1	barren homolog 1 (Drosophila)	
RACGAP1	Rac GTPase activating protein 1	NM_013277
ZNF278	zinc finger protein 278	NM_014323
TACC2	transforming, acidic coiled-coil containing protein 2	
TTK	TTK protein kinase	
FAM53B	family with sequence similarity 53, member B	NM_014661
DKFZP761M1511	hypothetical protein DKFZP761M1511	AK096661
RPN1	ribophorin I	
MYBBP1A	MYB binding protein (P160) 1a	
KIAA0152	KIAA0152	NM_014730
LOC90120	hypothetical gene supported by AK023162	BC013282
PIK3R3	phosphoinositide-3-kinase, regulatory subunit 3 (p55, gamma)	NM_003629
PPRC1	peroxisome proliferative activated receptor, gamma, coactivator-related 1	
CIT	citron (rho-interacting, serine/threonine kinase 21)	NM_007174
POLG	polymerase (DNA directed), gamma	
PPP1R10	protein phosphatase 1, regulatory subunit 10	
KIAA0090	KIAA0090	
HIRA	HIR histone cell cycle regulation defective homolog A (S. cerevisiae)	
DYNC1H1	dynein, cytoplasmic 1, heavy chain 1	NM_001376
DERPC	decreased expression in renal and prostate	NM_017804
PIK3C2B	phosphoinositide-3-kinase, class 2, beta polypeptide	
PTTG1	pituitary tumor-transforming 1	
CENTG2	centaurin, gamma 2	
STK35	serine/threonine kinase 35	NM_080836
LOC146909	hypothetical protein LOC146909	BC044933
AKR1B10	aldo-keto reductase family 1, member B10 (aldose reductase)	
WDR51A	WD repeat domain 51A	
CCNB1	cyclin B1	
HNRPL	heterogeneous nuclear ribonucleoprotein L	NM_001533
C22orf5	chromosome 22 open reading frame 5	
AYTL2	acyltransferase like 2	
CKS2	CDC28 protein kinase regulatory subunit 2	
TUBA8	tubulin, alpha 8	
RCC2	regulator of chromosome condensation 2	NM_018715
HNRPM	heterogeneous nuclear ribonucleoprotein M	
TUBA2	tubulin, alpha 2	
APLP2	amyloid beta (A4) precursor-like protein 2	
RPAP1	RNA polymerase II associated protein 1	

APH1A	anterior pharynx defective 1 homolog A (C. elegans)	
HSPA9B	heat shock 70kDa protein 9B (mortalin-2)	NM_004134
ZFP36	zinc finger protein 36, C3H type, homolog (mouse)	
SMG7	Smg-7 homolog, nonsense mediated mRNA decay factor (C. elegans)	
ODF2	outer dense fiber of sperm tails 2	
GABBR1	gamma-aminobutyric acid (GABA) B receptor, 1	NM_001470
CNN2	calponin 2	NM_004368
TNFAIP2	tumor necrosis factor, alpha-induced protein 2	
SPBC24	spindle pole body component 24 homolog (S. cerevisiae)	
KIAA0101	KIAA0101	
NEK2	NIMA (never in mitosis gene a)-related kinase 2	NM_002497
SH3PXD2B	SH3 and PX domains 2B	AB037716
KIAA1967	KIAA1967	
AKT1S1	AKT1 substrate 1 (proline-rich)	
LOC92755	hypothetical gene LOC92755	XM_047083
LMNB2	lamin B2	
TOLLIP	toll interacting protein	NM_019009
RPL39L	ribosomal protein L39-like	
FANCC	Fanconi anemia, complementation group C	
EHD4	EH-domain containing 4	
TUBB2B	tubulin, beta 2B	
SLC7A5	solute carrier family 7 (cationic amino acid transporter, y+ system), member 5	
IQGAP3	IQ motif containing GTPase activating protein 3	
CLASP1	cytoplasmic linker associated protein 1	
SF1	splicing factor 1	
N-PAC	cytokine-like nuclear factor n-pac	NM_032569
DKFZp564J157	DKFZp564J157 protein	
RBM14	RNA binding motif protein 14	
CCNF	cyclin F	
CORO2B	coronin, actin binding protein, 2B	
SLC1A5	solute carrier family 1 (neutral amino acid transporter), member 5	NM_005628
CDCA8	cell division cycle associated 8	
UBN1	ubinuclein 1	
SPAG5	sperm associated antigen 5	
TACC3	transforming, acidic coiled-coil containing protein 3	
CDC25C	cell division cycle 25C	
PHF15	PHD finger protein 15	NM_015288
TCOF1	Treacher Collins-Franceschetti syndrome 1	
DDIT4	DNA-damage-inducible transcript 4	
CDC20	CDC20 cell division cycle 20 homolog (S. cerevisiae)	
PRC1	protein regulator of cytokinesis 1	
NUMBL	numb homolog (Drosophila)-like	
CENPF	centromere protein F, 350/400ka (mitosin)	
CDCA2	cell division cycle associated 2	
TIMELESS	timeless homolog (Drosophila)	
SHCBP1	SHC SH2-domain binding protein 1	
CCNK	cyclin K	
APOBEC3B	apolipoprotein B mRNA editing enzyme, catalytic polypeptide-like 3B	
WHSC2	Wolf-Hirschhorn syndrome candidate 2	
MT1X	metallothionein 1X	
MICAL1	microtubule associated monoxygenase, calponin and LIM domain containing 1	
VAMP2	vesicle-associated membrane protein 2 (synaptobrevin 2)	NM_014232
LOC144097	hypothetical protein BC007540	
PCBP4	poly(rC) binding protein 4	
TSC2	tuberous sclerosis 2	
SPPL2B	signal peptide peptidase-like 2B	
raptor	raptor	
EFNB1	ephrin-B1	
TBC1D10B	TBC1 domain family, member 10B	
LMNA	lamin A/C	NM_170707
SMYD5	SMYD family member 5	
TFE3	transcription factor binding to IGHM enhancer 3	
ARHGEF1	Rho guanine nucleotide exchange factor (GEF) 1	
SLC4A2	solute carrier family 4, anion exchanger, member 2 (erythrocyte membrane protein band 3-like 1)	
ENTPD6	ectonucleoside triphosphate diphosphohydrolase 6 (putative function)	
BAI2	brain-specific angiogenesis inhibitor 2	
KIAA0460	KIAA0460	
LOC56930	hypothetical protein from EUROIMAGE 1669387	BC063667
TROAP	trophinin associated protein (tastin)	
DGKQ	diacylglycerol kinase, theta 110kDa	
LOC286167	hypothetical protein LOC286167	BX537506
COPS7B	COP9 constitutive photomorphogenic homolog subunit 7B (Arabidopsis)	
INPP5E	inositol polyphosphate-5-phosphatase, 72 kDa	
LRRC54	leucine rich repeat containing 54	
FLNC	filamin C, gamma (actin binding protein 280)	
ATXN7L3	ataxin 7-like 3	BC037418
NHN1	conserved nuclear protein NHN1	
PPP2R1A	protein phosphatase 2 (formerly 2A), regulatory subunit A (PR 65), alpha isoform	

AKT1	v-akt murine thymoma viral oncogene homolog 1	
NOC2L	nucleolar complex associated 2 homolog (S. cerevisiae)	NM_015658
BIRC5	baculoviral IAP repeat-containing 5 (survivin)	NM_001168
GIT1	G protein-coupled receptor kinase interactor 1	
DGCR2	DiGeorge syndrome critical region gene 2	NM_005137
CSPG4	chondroitin sulfate proteoglycan 4 (melanoma-associated)	
FAM38A	family with sequence similarity 38, member A	
UBQLN4P	ubiquilin 4 pseudogene	XM_209569
SENP3	SUMO1/sentrin/SMT3 specific peptidase 3	
AAAS	achalasia, adrenocortical insufficiency, alacrimia (Allgrove, triple-A)	
CIZ1	CDKN1A interacting zinc finger protein 1	
DOK4	docking protein 4	NM_018110
ADCY6	adenylate cyclase 6	
CDCA5	cell division cycle associated 5	
GPT2	glutamic pyruvate transaminase (alanine aminotransferase) 2	
FLJ20297	hypothetical protein FLJ20297	
CDR2L	cerebellar degeneration-related protein 2-like	
LOC392528	hypothetical LOC392528	XM_373366
SKIV2L	superkiller viralicidic activity 2-like (S. cerevisiae)	
BAT3	HLA-B associated transcript 3	
FAM64A	family with sequence similarity 64, member A	NM_019013
FXR2	fragile X mental retardation, autosomal homolog 2	
PPP2R5D	protein phosphatase 2, regulatory subunit B (B56), delta isoform	
LIG1	ligase I, DNA, ATP-dependent	
PYGB	phosphorylase, glycogen; brain	
UBE1	ubiquitin-activating enzyme E1 (A1S9T and BN75 temperature sensitivity complementing)	
PC	pyruvate carboxylase	
PML	promyelocytic leukemia	
C9orf48	chromosome 9 open reading frame 48	
SFXN5	sideroflexin 5	
PCGF2	polycomb group ring finger 2	NM_007144
PLEKHG4	pleckstrin homology domain containing, family G (with RhoGef domain) member 4	
ATG9A	ATG9 autophagy related 9 homolog A (S. cerevisiae)	
MLL4	myeloid/lymphoid or mixed-lineage leukemia 4	
FOXM1	forkhead box M1	
C1orf60	chromosome 1 open reading frame 60	
UBE2S	ubiquitin-conjugating enzyme E2S	NM_014501
DDEFL1	development and differentiation enhancing factor-like 1	
DAB2IP	DAB2 interacting protein	
PKN1	protein kinase N1	
B4GALT2	UDP-Gal:betaGlcNAc beta 1,4- galactosyltransferase, polypeptide 2	
FBS1	fibrosin 1	
FLJ11286	hypothetical protein FLJ11286	
RIS1	Ras-induced senescence 1	
RAB5C	RAB5C, member RAS oncogene family	NM_201434
SCMH1	sex comb on midleg homolog 1 (Drosophila)	
PPP1R14B	protein phosphatase 1, regulatory (inhibitor) subunit 14B	NM_138689
LOC388161	LOC388161	BC054509
GTPBP2	GTP binding protein 2	
PRCC	papillary renal cell carcinoma (translocation-associated)	
PTK7	PTK7 protein tyrosine kinase 7	
DKFZp762E1312	hypothetical protein DKFZp762E1312	
CBS	cystathionine-beta-synthase	
CTBP1	C-terminal binding protein 1	
TTYH3	tweety homolog 3 (Drosophila)	
UBE2E2	ubiquitin-conjugating enzyme E2E 2 (UBC4/5 homolog, yeast)	
LOC388152	hypothetical protein FLJ90297	NM_203426
MICAL3	microtubule associated monoxygenase, calponin and LIM domain containing 3	AB020626
MTA1	metastasis associated 1	
FAM18B2	family with sequence similarity 18, member B2	
FAM62A	family with sequence similarity 62 (C2 domain containing), member A	
MKI67	antigen identified by monoclonal antibody Ki-67	
CUTL1	cut-like 1, CCAAT displacement protein (Drosophila)	
OS9	amplified in osteosarcoma	
PLK1	polo-like kinase 1 (Drosophila)	NM_005030
GLI1	glioma-associated oncogene homolog 1 (zinc finger protein)	
CDCA3	cell division cycle associated 3	
HMGA1	high mobility group AT-hook 1	NM_145904
MCOLN1	mucolipin 1	
OGDH	oxoglutarate (alpha-ketoglutarate) dehydrogenase (lipoamide)	
GPI	glucose phosphate isomerase	NM_000175
ACIN1	apoptotic chromatin condensation inducer 1	
UBE2C	ubiquitin-conjugating enzyme E2C	
ARS2	ARS2 protein	
FREQ	frequenin homolog (Drosophila)	NM_014286
PRKACA	protein kinase, cAMP-dependent, catalytic, alpha	NM_002730
FLJ12529	pre-mRNA cleavage factor I, 59 kDa subunit	
TCF3	transcription factor 3 (E2A immunoglobulin enhancer binding factors E12/E47)	

|MLF2| myeloid leukemia factor 2 | |
|YWHAE| tyrosine 3-monooxygenase/tryptophan 5-monooxygenase activation protein, epsilon polypeptide | NM_006761 |
|MARCKSL1| MARCKS-like 1 | |
|MKNK2| MAP kinase interacting serine/threonine kinase 2 | |
|PIK4CB| phosphatidylinositol 4-kinase, catalytic, beta polypeptide | |
|KHSRP| KH-type splicing regulatory protein (FUSE binding protein 2) | NM_003685 |
|C1D| nuclear DNA-binding protein | |
|C13orf8| chromosome 13 open reading frame 8 | |
|FLJ34443| hypothetical protein FLJ34443 | |
|RCE1| RCE1 homolog, prenyl protein peptidase (S. cerevisiae) | |
|GANAB| glucosidase, alpha; neutral AB | |
|MID1IP1| MID1 interacting protein 1 (gastrulation specific G12-like (zebrafish)) | |
|UNC5B| unc-5 homolog B (C. elegans) | |
|SPTAN1| spectrin, alpha, non-erythrocytic 1 (alpha-fodrin) | NM_003127 |
|PKMYT1| protein kinase, membrane associated tyrosine/threonine 1 | |
|ASCC2| activating signal cointegrator 1 complex subunit 2 | |
|UBE2M| ubiquitin-conjugating enzyme E2M (UBC12 homolog, yeast) | |
|SMTN| smoothelin | NM_134269 |
|DCTN1| dynactin 1 (p150, glued homolog, Drosophila) | |
|PKM2| pyruvate kinase, muscle | |
|APLP1| amyloid beta (A4) precursor-like protein 1 | |
|FOSL1| FOS-like antigen 1 | |
|ANKRD52| ankyrin repeat domain 52 | |
|SAFB2| scaffold attachment factor B2 | |
|FGFR4| fibroblast growth factor receptor 4 | |
|LARP1| La ribonucleoprotein domain family, member 1 | NM_015315 |
|PYGO2| pygopus homolog 2 (Drosophila) | |
|FKBP1C| FK506 binding protein 1C | NM_001011510 |
|ATP2A2| ATPase, Ca++ transporting, cardiac muscle, slow twitch 2 | NM_170665 |
|PIK3R2| phosphoinositide-3-kinase, regulatory subunit 2 (p85 beta) | |
|KIAA1698| KIAA1698 | |
|LENG8| leukocyte receptor cluster (LRC) member 8 | |
|POLR2A| polymerase (RNA) II (DNA directed) polypeptide A, 220kDa | NM_000937 |
|FKBP1A| FK506 binding protein 1A, 12kDa | |
|RAB1B| RAB1B, member RAS oncogene family | NM_030981 |
|TLN1| talin 1 | |
|PTMS| parathymosin | |
|C9orf74| chromosome 9 open reading frame 74 | |
|FSCN1| fascin homolog 1, actin-bundling protein (Strongylocentrotus purpuratus) | NM_003088 |
|PLTP| phospholipid transfer protein | |
|G6PD| glucose-6-phosphate dehydrogenase | |
|GRINA| glutamate receptor, ionotropic, N-methyl D-asparate-associated protein 1 (glutamate binding) | |
|SOCS1| suppressor of cytokine signaling 1 | |
|CNOT3| CCR4-NOT transcription complex, subunit 3 | |
|RFX1| regulatory factor X, 1 (influences HLA class II expression) | |
|MEN1| multiple endocrine neoplasia I | |
|HECTD3| HECT domain containing 3 | |
|NXPH4| neurexophilin 4 | |
|RARG| retinoic acid receptor, gamma | |
|FAM86B1| family with sequence similarity 86, member B1 | |
|CRABP2| cellular retinoic acid binding protein 2 | |
|TMEM76| transmembrane protein 76 | CR749838 |
|SF3B4| splicing factor 3b, subunit 4, 49kDa | |
|DGCR8| DiGeorge syndrome critical region gene 8 | |
|CALM3| calmodulin 3 (phosphorylase kinase, delta) | |
|ANKRD11| ankyrin repeat domain 11 | |
|PPP4C| protein phosphatase 4 (formerly X), catalytic subunit | |
|RKHD1| ring finger and KH domain containing 1 | |
|SRC| v-src sarcoma (Schmidt-Ruppin A-2) viral oncogene homolog (avian) | |
