# Supplementary figures and images for: IGF-I induced genes in stromal fibroblasts predict the clinical outcome of breast and lung cancer patients
Source: BMC Med. 2010 Jan 5;8:1. doi: 10.1186/1741-7015-8-1 (PMC2823652; doi:10.1186/1741-7015-8-1)

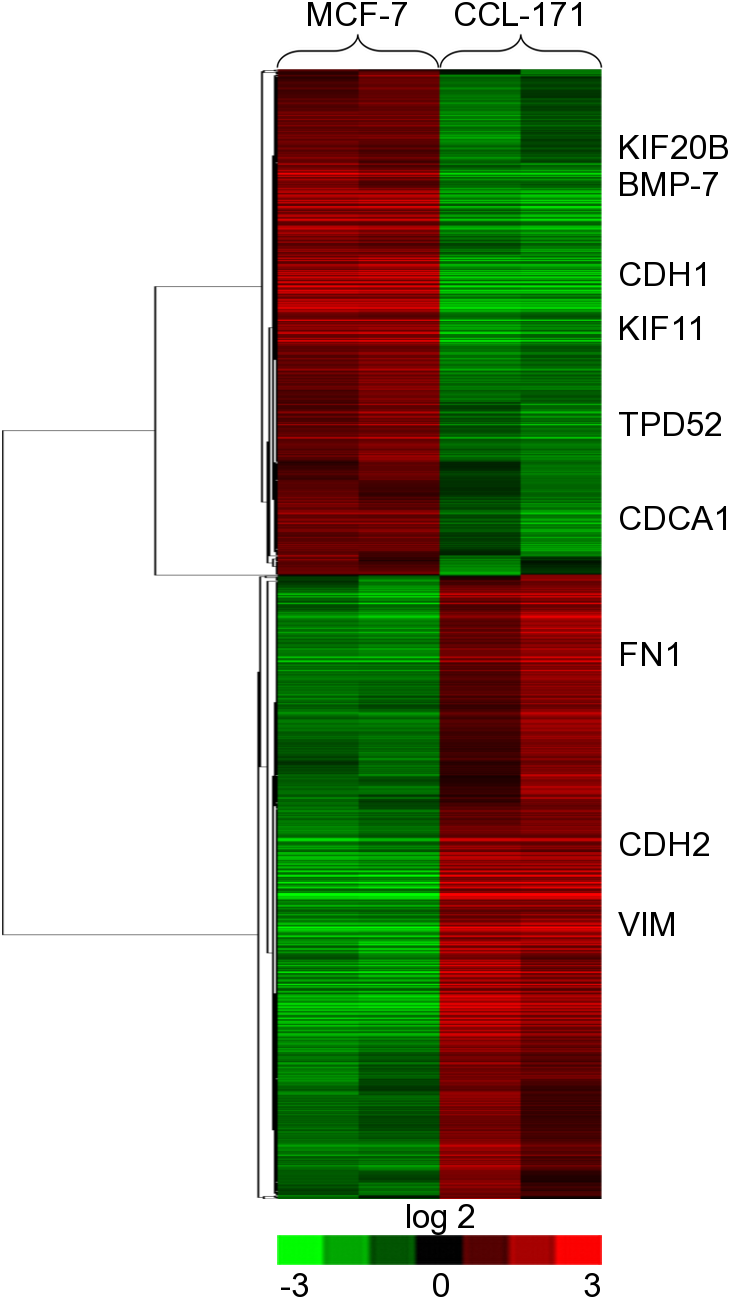

Supplement: Additional file 3 — Figure S1. Distinct default gene expression profiles of human lung fibroblasts and breast tumour cells. Genes are presented in rows and experiments in columns. Both cell types demonstrate a clearly distinct default gene expression profile, typical for epithelial and mesenchymal cells. Gene markers typical for mesenchymal (FN1, CDH2, VIM) and epithelial/tumour cells (CDH1, TPD52, BMP-7) are marked. Additionally, examples of proliferation associated genes up-regulated in MCF-7 cells by default are shown. [file 1741-7015-8-1-S3.TIFF]

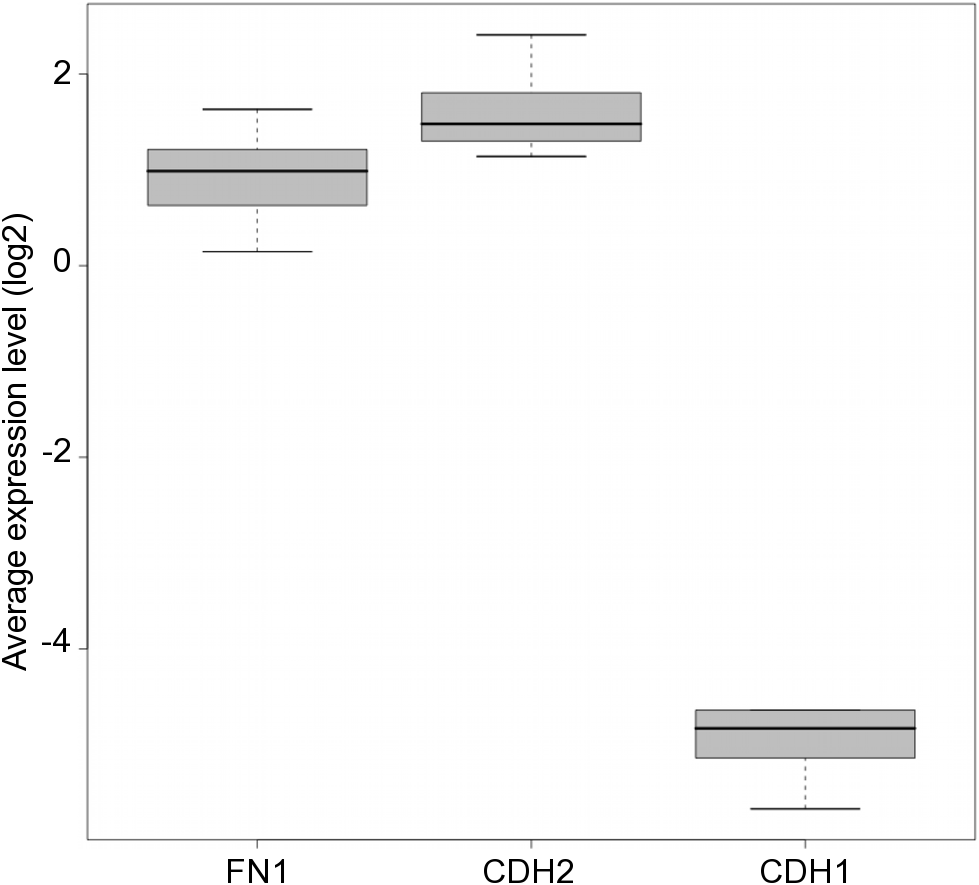

Supplement: Additional file 4 — Figure S3. Box-and-whisker plot illustrating the average expression level of fibronectin (FN1), N-cadherin (CDH2) and E-cadherin (CDH1) in primary fibroblasts. Insulin-like growth factor (IGF-I) does not affect the expression level of mesenchymal and epithelial markers in primary breast fibroblasts (data not shown). [file 1741-7015-8-1-S4.TIFF]

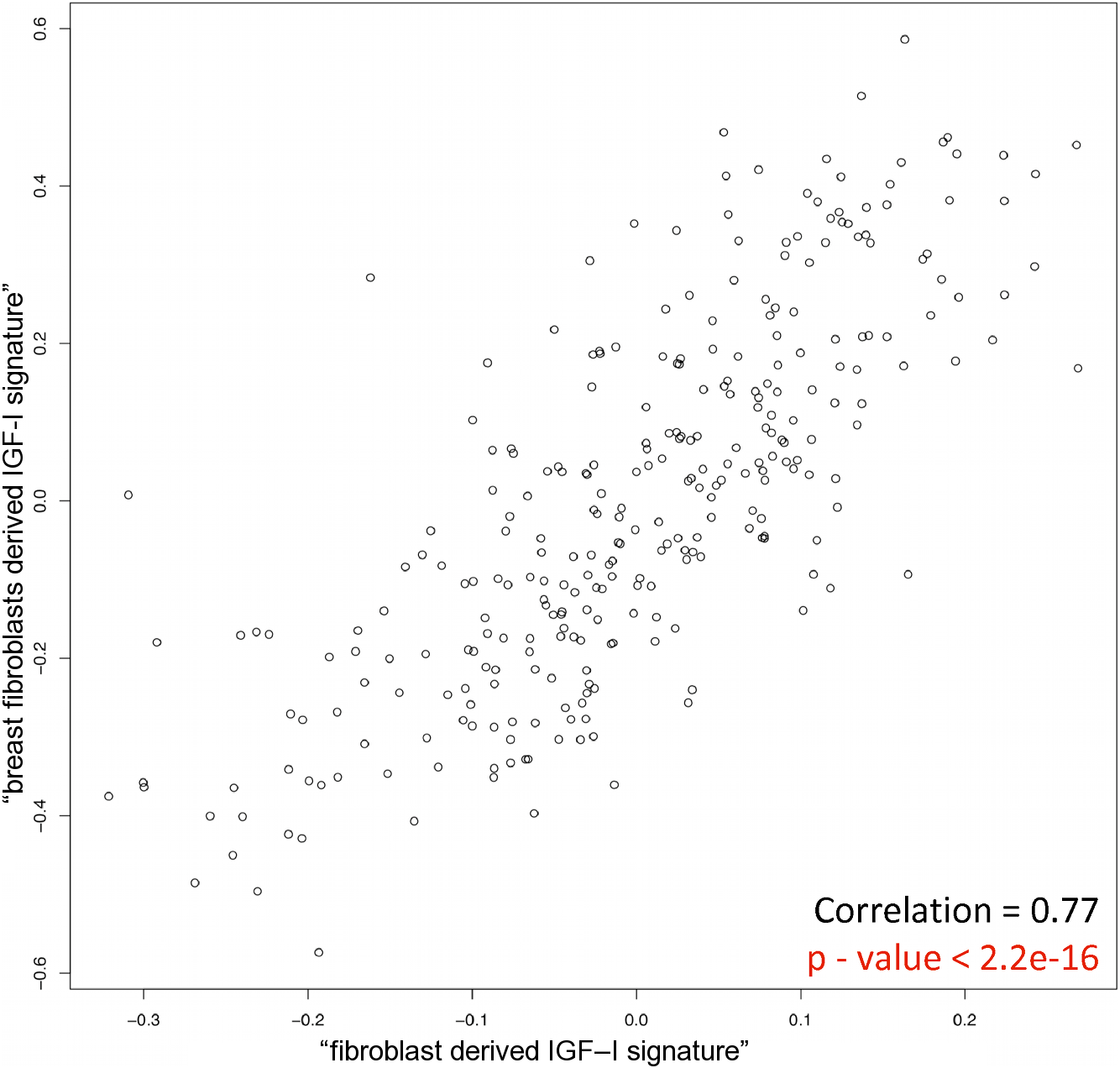

Supplement: Additional file 10 — Figure S5. Correlation of the fibroblast derived insulin-like growth factor-1 (IGF-I) signature and the breast fibroblast IGF-I induced signature centroids in the Netherlands Cancer Institute dataset. Pearson correlations for the signature and the P value are shown in the lower right part of the plot. [file 1741-7015-8-1-S10.TIFF]
