# Supplementary material for: IGF-I induced genes in stromal fibroblasts predict the clinical outcome of breast and lung cancer patients
Source: BMC Med. 2010 Jan 5;8:1. doi: 10.1186/1741-7015-8-1 (PMC2823652; doi:10.1186/1741-7015-8-1)
Supplement: Additional file 5 — Table S2. List of genes up-regulated (the breast fibroblast derived insulin-like growth factor-1 [IGF-I] signature) and down-regulated in primary breast fibroblasts upon IGF-I stimulation. [file 1741-7015-8-1-S5.PDF]

Genes up-regulated (208)

Gene Name	Gene ID	Fold Change	q-value(%)
|| ChaC, cation transport regulator-like 1 (E. coli) || Hs.155569 || 79094 || || hSQ006974 || || CHAC1 || hSQ006974 || || hHC021936 || 415322	hSQ006974	4.96	0
|| CDC28 protein kinase regulatory subunit 2::CDC28 protein kinase regulatory subunit 2 || Hs.83758 || 1164 || || hSQ008541 || || CKS2 || hSQ008541 || || hHC029222 || 340547	hSQ008541	3.70	0
|| Family with sequence similarity 64, member A::family with sequence similarity 64, member A || Hs.592116 || 54478 || || hSQ037412 || || FAM64A || hSQ037412 || || hHC029674 || 407650	hSQ037412	3.08	0
|| Ubiquitin-conjugating enzyme E2C::ubiquitin-conjugating enzyme E2C || Hs.93002 || 11065 || || hSQ003297 || || UBE2C || hSQ003297 || || hHC028811 || 411680	hSQ003297	3.69	0
|| Family with sequence similarity 64, member A::family with sequence similarity 64, member A || Hs.592116 || 54478 || NM_019013 || hSQ036488 || || FAM64A || hSQ036488 || || hHR029697 || 398068	hSQ036488	3.09	0
|| Ras association (RalGDS/AF-6) domain family member 2::Ras association (RalGDS/AF-6) domain family member 2 || Hs.631504 || 9770 || || hSQ025344 || || RASSF2 || hSQ025344 || || hHC003792 || 415760	hSQ025344	3.07	0
|| Cellular retinoic acid binding protein 2::cellular retinoic acid binding protein 2 || Hs.405662 || 1382 || || hSQ006997 || || CRABP2 || hSQ006997 || || hHC022599 || 349982	hSQ006997	3.58	0
|| Apolipoprotein B mRNA editing enzyme, catalytic polypeptide-like 3B::apolipoprotein B mRNA editing enzyme, catalytic polypeptide-like 3B || Hs.226307 || 9582 || || hSQ015626 || || APOBEC3B || hSQ015626 || || hHC003502 || 405368	hSQ015626	3.38	0
|| Centromere protein M::centromere protein M || Hs.208912 || 79019 || || hSQ021233 || || CENPM || hSQ021233 || || hHC015970 || 404691	hSQ021233	3.67	0
|| Holliday junction recognition protein::Holliday junction recognition protein || Hs.532968 || 55355 || || hSQ021705 || || HJURP || hSQ021705 || || hHC007597 || 333862	hSQ021705	3.93	0
|| Asparagine synthetase::asparagine synthetase || Hs.489207 || 440 || NM_133436 || hSQ005455 || || ASNS || hSQ005455 || || hHR027072 || 413696	hSQ005455	3.01	0
|| tubulin, alpha 1a::Tubulin, alpha 1a || Hs.654422 || 7846 || || hSQ023443 || || TUBA1A || hSQ023443 || || hHC025916 || 387235	hSQ023443	2.69	0
|| Eukaryotic translation initiation factor 4E binding protein 1::eukaryotic translation initiation factor 4E binding protein 1 || Hs.411641 || 1978 || NM_004095 || hSQ001721 || || EIF4EBP1 || hSQ001721 || || hHR027752 || 391632	hSQ001721	2.80	0
|| cell division cycle 20 homolog (S. cerevisiae)::Cell division cycle 20 homolog (S. cerevisiae) || Hs.524947 || 991 || NM_001255 || hSQ014543 || || CDC20 || hSQ014543 || || hHR029265 || 393560	hSQ014543	2.81	0
|| Cell division cycle associated 3::cell division cycle associated 3 || Hs.524216 || 83461 || || hSQ010072 || || CDCA3 || hSQ010072 || || hHC020474::hHA038355 || 340332	hSQ010072	2.89	0
|| Myosin, light chain 9, regulatory::myosin, light chain 9, regulatory || Hs.504687 || 10398 || || hSQ022900 || || MYL9 || hSQ022900 || || hHA032899 || 381118	hSQ022900	2.83	0
|| Chromosome 6 open reading frame 173::chromosome 6 open reading frame 173 || Hs.486401 || 387103 || NM_001012507 || hSQ034030 || || C6orf173 || hSQ034030 || || hHR011618 || 342182	hSQ034030	2.86	0
|| Opa interacting protein 5::Opa interacting protein 5 || Hs.661645 || 11339 || || hSQ009372 || || OIP5 || hSQ009372 || || hHC022591 || 382715	hSQ009372	2.61	0
|| Coatomer protein complex, subunit zeta 2::coatomer protein complex, subunit zeta 2 || Hs.408434 || 51226 || || hSQ006358 || || COPZ2 || hSQ006358 || || hHA035813 || 369014	hSQ006358	2.68	0
|| Thymidine kinase 1, soluble::thymidine kinase 1, soluble || Hs.515122 || 7083 || || hSQ043587 || || TK1 || hSQ043587 || || hHC021592 || 354686	hSQ043587	2.86	0
|| Aurora kinase A::aurora kinase A || Hs.250822 || 6790 || || hSQ022798 || || AURKA || hSQ022798 || || hHC023951 || 411257	hSQ022798	2.74	0
|| Proline/serine-rich coiled-coil 1::proline/serine-rich coiled-coil 1 || Hs.405925 || 84722 || || hSQ021168 || || PSRC1 || hSQ021168 || || hHC005405 || 366623	hSQ021168	2.53	0
|| Sperm associated antigen 5::sperm associated antigen 5 || Hs.514033 || 10615 || || hSQ016854 || || SPAG5 || hSQ016854 || || hHC006208 || 355390	hSQ016854	2.62	0
|| Centromere protein E, 312kDa::centromere protein E, 312kDa || Hs.75573 || 1062 || || hSQ034356 || || CENPE || hSQ034356 || || hHC014176 || 356551	hSQ034356	2.61	0
|| Phosphoglycerate dehydrogenase::phosphoglycerate dehydrogenase || Hs.487296 || 26227 || || hSQ018094 || || PHGDH || hSQ018094 || || hHC025080 || 416663	hSQ018094	2.80	0
|| Effector cell peptidase receptor 1::baculoviral IAP repeat-containing 5 (survivin) || Hs.514527 || 332 || NM_001168 || hSQ040118 || || BIRC5 || hSQ040118 || || hHR015831 || 346290	hSQ040118	2.90	0
|| Coatomer protein complex, subunit zeta 2::coatomer protein complex, subunit zeta 2 || Hs.408434 || 51226 || || hSQ040932 || || COPZ2 || hSQ040932 || || hHA040149 || 400573	hSQ040932	2.46	0
|| Cell division cycle associated 8::cell division cycle associated 8 || Hs.524571::Hs.696292 || 55143 || || hSQ038003 || || CDCA8 || hSQ038003 || || hHC021045 || 400041	hSQ038003	3.11	0
|| Transcribed locus::pituitary tumor-transforming 3 || Hs.545401::Hs.647156 || 26255 || AF095289 || hSQ017799 || || PTTG3 || hSQ017799 || || hHR028333 || 334815	hSQ017799	2.94	0
|| Phosphoenolpyruvate carboxykinase 2 (mitochondrial)::phosphoenolpyruvate carboxykinase 2 (mitochondrial) || Hs.75812 || 5106 || || hSQ014606 || || PCK2 || hSQ014606 || || hHC021213 || 394355	hSQ014606	2.76	0
|| Trophinin associated protein (tastin)::trophinin associated protein (tastin) || Hs.524399 || 10024 || || hSQ012497 || || TROAP || hSQ012497 || || hHC021451 || 383157	hSQ012497	2.91	0
|| TTK protein kinase::TTK protein kinase || Hs.169840 || 7272 || || hSQ020130 || || TTK || hSQ020130 || || hHC006917 || 368828	hSQ020130	3.23	0
|| Cyclin B1::cyclin B1 || Hs.23960 || 891 || || hSQ021103 || || CCNB1 || hSQ021103 || || hHC016577 || 364113	hSQ021103	3.30	0
|| SHC SH2-domain binding protein 1::SHC SH2-domain binding protein 1 || Hs.123253 || 79801 || || hSQ008055 || || SHCBP1 || hSQ008055 || || hHC003756 || 416836	hSQ008055	3.05	0
|| Tropomyosin 2 (beta)::tropomyosin 2 (beta) || Hs.300772 || 7169 || || hSQ034802 || || TPM2 || hSQ034802 || || hHA039194 || 340362	hSQ034802	2.49	0
|| Family with sequence similarity 83, member D::family with sequence similarity 83, member D || Hs.472716 || 81610 || || hSQ031892 || || FAM83D || hSQ031892 || || hHC009603 || 352018	hSQ031892	3.27	0
|| Phosphoserine aminotransferase 1::phosphoserine aminotransferase 1 || Hs.494261 || 29968 || || hSQ004879 || || PSAT1 || hSQ004879 || || hHC028420 || 360477	hSQ004879	3.50	0
|| Tribbles homolog 3 (Drosophila)::tribbles homolog 3 (Drosophila) || Hs.516826 || 57761 || || hSQ026522 || || TRIB3 || hSQ026522 || || hHC008792 || 387410	hSQ026522	2.80	0
|| Tubulin, beta 2C::tubulin, beta 2C || Hs.433615 || 10383 || || hSQ030977 || || TUBB2C || hSQ030977 || || hHC028398 || 370626	hSQ030977	2.52	0
|| NUF2, NDC80 kinetochore complex component, homolog (S. cerevisiae)::NUF2, NDC80 kinetochore complex component, homolog (S. cerevisiae) || Hs.651950 || 83540 || || hSQ028788 || || NUF2 || hSQ028788 || || hHC008847 || 350999	hSQ028788	4.04	0
|| Fatty acid binding protein 5 (psoriasis-associated)::fatty acid binding protein 5 (psoriasis-associated) || Hs.408061 || 2171 || NM_001444 || hSQ043307 || || FABP5 || hSQ043307 || || hHR031203 || 352047	hSQ043307	2.59	0
|| Nucleolar and spindle associated protein 1::nucleolar and spindle associated protein 1 || Hs.615092 || 51203 || || hSQ045304 || || NUSAP1 || hSQ045304 || || hHC011295 || 348138	hSQ045304	3.36	0

|| Deoxythymidylate kinase (thymidylate kinase)::deoxythymidylate kinase (thymidylate kinase) || Hs.471873::Hs.599355 || 1841 || NM_012145 || hSQ037073 || || DTYMK || hSQ037073 || || hHR030654 || 373910	hSQ037073	2.49	0
|| Chromosome 13 open reading frame 3::chromosome 13 open reading frame 3 || Hs.88523 || 221150 || || hSQ012681 || || C13orf3 || hSQ012681 || || hHC012244 || 355782	hSQ012681	3.11	0
|| Kinesin family member 22::kinesin family member 22 || Hs.613351 || 3835 || || hSQ039201 || || KIF22 || hSQ039201 || || hHC016722 || 376708	hSQ039201	2.22	0
|| TUBB4Q pseudogene || || 388579 || XM_371207 || hSQ001639 || || LOC388579 || hSQ001639 || || hHR030175 || 383231	hSQ001639	2.36	0
|| Capping protein (actin filament), gelsolin-like::capping protein (actin filament), gelsolin-like || Hs.516155 || 822 || || hSQ026880 || || CAPG || hSQ026880 || || hHC019395 || 351624	hSQ026880	2.16	0
|| similar to alpha tubulin || Hs.433336 || 112714 || || hSQ009149 || || LOC112714 || hSQ009149 || || hHC031671 || 346323	hSQ009149	2.69	0
|| CDC28 protein kinase regulatory subunit 1B::CDC28 protein kinase regulatory subunit 1B || Hs.374378 || 1163 || NM_001826 || hSQ021251 || || CKS1B || hSQ021251 || || hHR031105 || 334603	hSQ021251	2.33	0
|| Pituitary tumor-transforming 1::pituitary tumor-transforming 1 || Hs.350966 || 9232 || NM_004219 || hSQ038927 || || PTTG1 || hSQ038927 || || hHR027560 || 391380	hSQ038927	2.94	0
|| Activating transcription factor 5::activating transcription factor 5 || Hs.9754 || 22809 || || hSQ004955 || || ATF5 || hSQ004955 || || hHC020747 || 412152	hSQ004955	2.54	0
|| kinesin family member 18A::Kinesin family member 18A || Hs.301052 || 81930 || || hSQ015373 || || KIF18A || hSQ015373 || || hHC016862 || 339221	hSQ015373	2.46	0
|| chromosome 6 open reading frame 173 || || 387103 || || hSQ006036 || || C6orf173 || hSQ006036 || || hHE041995 || 381756	hSQ006036	2.69	0
|| Tubulin, alpha 8 || Hs.137400 || 51807 || || hSQ010026 || || TUBA8 || hSQ010026 || || hHC030029 || 362657	hSQ010026	2.61	0
|| Acyl-CoA thioesterase 7::acyl-CoA thioesterase 7 || Hs.126137 || 11332 || || hSQ008637 || || ACOT7 || hSQ008637 || || hHA033103 || 379282	hSQ008637	2.30	0
|| Discs, large (Drosophila) homolog-associated protein 5::discs, large (Drosophila) homolog-associated protein 5 || Hs.77695 || 9787 || NM_014750 || hSQ034642 || || DLGAP5 || hSQ034642 || || hHR003506 || 338483	hSQ034642	2.50	0
|| H2A histone family, member Z, pseudogene 2 || || 346990 || XM_294468 || hSQ018477 || || H2AFZP2 || hSQ018477 || || hHR028806 || 375393	hSQ018477	2.32	0
|| Centrosomal protein 55kDa::centrosomal protein 55kDa || Hs.14559 || 55165 || || hSQ024840 || || CEP55 || hSQ024840 || || hHC003148 || 352945	hSQ024840	2.82	0
|| Cyclin A2::cyclin A2 || Hs.58974 || 890 || || hSQ030148 || || CCNA2 || hSQ030148 || || hHC014459 || 409413	hSQ030148	2.54	0
|| Eukaryotic translation initiation factor 4E binding protein 1::eukaryotic translation initiation factor 4E binding protein 1 || Hs.411641 || 1978 || || hSQ040873 || || EIF4EBP1 || hSQ040873 || || hHC027722 || 335876	hSQ040873	2.49	0
|| Kinesin light chain 1::kinesin light chain 1 || Hs.20107 || 3831 || || hSQ005238 || || KLC1 || hSQ005238 || || hHA036872 || 392263	hSQ005238	2.14	0
|| Mitochondrial ribosomal protein L24::mitochondrial ribosomal protein L24 || Hs.418233 || 79590 || || hSQ010554 || || MRPL24 || hSQ010554 || || hHC021604 || 358976	hSQ010554	2.22	0
|| Centromere protein M::centromere protein M || Hs.208912 || 79019 || || hSQ006578 || || CENPM || hSQ006578 || || hHA040336 || 359068	hSQ006578	2.28	0
|| Ribonuclease H2, subunit A::ribonuclease H2, subunit A || Hs.532851 || 10535 || || hSQ003383 || || RNASEH2A || hSQ003383 || || hHC014803 || 383922	hSQ003383	2.29	0
|| BUB1 budding uninhibited by benzimidazoles 1 homolog beta (yeast)::BUB1 budding uninhibited by benzimidazoles 1 homolog beta (yeast) || Hs.631699 || 701 || || hSQ022216 || || BUB1B || hSQ022216 || || hHC002402 || 343456	hSQ022216	2.56	0
|| Yip1 interacting factor homolog B (S. cerevisiae)::Yip1 interacting factor homolog B (S. cerevisiae) || Hs.280741 || 90522 || || hSQ031144 || || YIF1B || hSQ031144 || || hHA040229 || 369148	hSQ031144	2.20	0
|| Ubiquitin-conjugating enzyme E2T (putative)::ubiquitin-conjugating enzyme E2T (putative) || Hs.5199 || 29089 || || hSQ030548 || || UBE2T || hSQ030548 || || hHC011962 || 403141	hSQ030548	2.61	0
|| Discs, large (Drosophila) homolog-associated protein 5::discs, large (Drosophila) homolog-associated protein 5 || Hs.77695 || 9787 || || hSQ044725 || || DLGAP5 || hSQ044725 || || hHC027729 || 393433	hSQ044725	2.45	0
|| Spindle pole body component 24 homolog (S. cerevisiae) || Hs.381225 || 147841 || || hSQ003544 || || SPBC24 || hSQ003544 || || hHC023882 || 336609	hSQ003544	2.40	0
|| Chloride intracellular channel 1::chloride intracellular channel 1 || Hs.414565 || 1192 || || hSQ009440 || || CLIC1 || hSQ009440 || || hHC027006 || 374851	hSQ009440	2.12	0
|| Casein kinase 1, alpha 1::casein kinase 1, alpha 1 || Hs.529862::Hs.654547::Hs.699188 || 1452 || NM_001892 || hSQ029276 || || CSNK1A1 || hSQ029276 || || hHR026924 || 390562	hSQ029276	2.53	0
|| Emopamil binding protein (sterol isomerase)::emopamil binding protein (sterol isomerase) || Hs.30619 || 10682 || || hSQ030583 || || EBP || hSQ030583 || || hHC014804 || 391676	hSQ030583	2.39	0
|| tubulin, alpha 3c || || 7278 || || hSQ009497 || || TUBA3C || hSQ009497 || || hHA033408 || 413030	hSQ009497	2.28	0
|| Antigen identified by monoclonal antibody Ki-67::antigen identified by monoclonal antibody Ki-67 || Hs.80976 || 4288 || || hSQ010942 || || MKI67 || hSQ010942 || || hHC010551 || 344889	hSQ010942	2.46	0
|| BolA homolog 3 (E. coli)::bolA homolog 3 (E. coli) || Hs.61472 || 388962 || NM_212552 || hSQ027603 || || BOLA3 || hSQ027603 || || hHR029286 || 376112	hSQ027603	2.34	0
|| Nucleolar and spindle associated protein 1::nucleolar and spindle associated protein 1 || Hs.615092 || 51203 || || hSQ019967 || || NUSAP1 || hSQ019967 || || hHA033857 || 364134	hSQ019967	2.19	0
|| Calponin 1, basic, smooth muscle::calponin 1, basic, smooth muscle || Hs.465929 || 1264 || || hSQ011762 || || CNN1 || hSQ011762 || || hHC001479 || 348535	hSQ011762	2.19	0
|| Cyclin B2::cyclin B2 || Hs.194698 || 9133 || NM_004701 || hSQ030658 || || CCNB2 || hSQ030658 || || hHR017719 || 379914	hSQ030658	2.20	0
|| Maternal embryonic leucine zipper kinase::maternal embryonic leucine zipper kinase || Hs.184339 || 9833 || || hSQ000814 || || MELK || hSQ000814 || || hHC003795 || 347619	hSQ000814	2.89	0
|| asparagine synthetase || || 440 || || hSQ044338 || || ASNS || hSQ044338 || || hHA033171 || 346394	hSQ044338	2.81	0
|| kinesin family member 20A::Kinesin family member 20A || Hs.73625 || 10112 || NM_005733 || hSQ022738 || || KIF20A || hSQ022738 || || hHR006196 || 355316	hSQ022738	2.59	0
|| Mevalonate kinase::mevalonate kinase || Hs.130607 || 4598 || || hSQ031664 || || MVK || hSQ031664 || || hHC010564 || 359617	hSQ031664	2.33	0
|| PDZ binding kinase::PDZ binding kinase || Hs.104741 || 55872 || || hSQ001473 || || PBK || hSQ001473 || || hHC002267 || 409986	hSQ001473	2.42	0
|| Phosphoserine aminotransferase 1::phosphoserine aminotransferase 1 || Hs.494261 || 29968 || || hSQ044506 || || PSAT1 || hSQ044506 || || hHA033352 || 357244	hSQ044506	2.61	0
|| Nurim (nuclear envelope membrane protein)::nurim (nuclear envelope membrane protein) || Hs.519993 || 11270 || || hSQ001186 || || NRM || hSQ001186 || || hHC020080 || 360971	hSQ001186	2.12	0
|| BUB1 budding uninhibited by benzimidazoles 1 homolog (yeast)::BUB1 budding uninhibited by benzimidazoles 1 homolog (yeast) || Hs.469649 || 699 || || hSQ010580 || || BUB1 || hSQ010580 || || hHC005725 || 330791	hSQ010580	2.26	0
|| Phosphoserine phosphatase::phosphoserine phosphatase || Hs.512656 || 5723 || || hSQ015035 || || PSPH || hSQ015035 || || hHC028278 || 372516	hSQ015035	2.19	0
|| Protein regulator of cytokinesis 1::protein regulator of cytokinesis 1 || Hs.567385 || 9055 || || hSQ024484 || || PRC1 || hSQ024484 || || hHC006562 || 405557	hSQ024484	2.70	0

|| SPC25, NDC80 kinetochore complex component, homolog (S. cerevisiae)::SPC25, NDC80 kinetochore complex component, homolog (S. cerevisiae) || Hs.421956 || 57405 || || hSQ028486 || || SPC25 || hSQ028486 || || hHC001782 || 371681	hSQ028486	2.66	0
|| Pituitary tumor-transforming 1::pituitary tumor-transforming 1 || Hs.350966 || 9232 || || hSQ012349 || || PTTG1 || hSQ012349 || || hHC029136 || 415326	hSQ012349	2.67	0
|| Rac GTPase activating protein 1 || Hs.505469::Hs.696319::Hs.653269 || 29127 || NM_013277 || hSQ036136 || || RACGAP1 || hSQ036136 || || hHR028857 || 394975	hSQ036136	2.27	0
|| Cell division cycle 37 homolog (S. cerevisiae)::cell division cycle 37 homolog (S. cerevisiae) || Hs.160958 || 11140 || || hSQ001559 || || CDC37 || hSQ001559 || || hHC029961 || 400371	hSQ001559	2.02	0
|| LSM4 homolog, U6 small nuclear RNA associated (S. cerevisiae)::LSM4 homolog, U6 small nuclear RNA associated (S. cerevisiae) || Hs.515255 || 25804 || || hSQ033151 || || LSM4 || hSQ033151 || || hHC016675 || 380543	hSQ033151	2.06	0
|| Cyclin-dependent kinase inhibitor 3 (CDK2-associated dual specificity phosphatase)::cyclin-dependent kinase inhibitor 3 (CDK2-associated dual specificity phosphatase) || Hs.84113 || 1033 || || hSQ015724 || || CDKN3 || hSQ015724 || || hHA034304 || 390779	hSQ015724	2.18	0
|| family with sequence similarity 72, member A || Hs.535577 || 389835 || || hSQ004466 || || FAM72A || hSQ004466 || || hHC013375 || 381913	hSQ004466	2.36	0
|| Filamin C, gamma (actin binding protein 280)::filamin C, gamma (actin binding protein 280) || Hs.58414 || 2318 || || hSQ008734 || || FLNC || hSQ008734 || || hHC020553 || 399298	hSQ008734	2.22	0
|| High-mobility group box 2::high-mobility group box 2 || Hs.434953 || 3148 || || hSQ039860 || || HMGB2 || hSQ039860 || || hHC015229 || 329901	hSQ039860	2.22	0
|| SWI/SNF related, matrix associated, actin dependent regulator of chromatin, subfamily d, member 3::SWI/SNF related, matrix associated, actin dependent regulator of chromatin, subfamily d, member 3 || Hs.647067 || 6604 || || hSQ006237 || || SMARCD3 || hSQ006237 || || hHC024690 || 375748	hSQ006237	2.02	0
|| Cyclin-dependent kinase inhibitor 3 (CDK2-associated dual specificity phosphatase)::cyclin-dependent kinase inhibitor 3 (CDK2-associated dual specificity phosphatase) || Hs.84113 || 1033 || || hSQ035649 || || CDKN3 || hSQ035649 || || hHA032350 || 401552	hSQ035649	2.06	0
|| MAX dimerization protein 3 || Hs.693703::Hs.653158::Hs.699373 || 83463 || || hSQ041390 || || MXD3 || hSQ041390 || || hHC025244 || 386367	hSQ041390	2.59	0
|| Solute carrier family 7 (cationic amino acid transporter, y+ system), member 5::solute carrier family 7 (cationic amino acid transporter, y+ system), member 5 || Hs.513797 || 8140 || || hSQ022783 || || SLC7A5 || hSQ022783 || || hHC016251 || 349058	hSQ022783	2.23	0
|| Topoisomerase (DNA) II alpha 170kDa::topoisomerase (DNA) II alpha 170kDa || Hs.156346 || 7153 || || hSQ024611 || || TOP2A || hSQ024611 || || hHC004023 || 345873	hSQ024611	2.50	0
|| Meiotic nuclear divisions 1 homolog (S. cerevisiae)::meiotic nuclear divisions 1 homolog (S. cerevisiae) || Hs.294088 || 84057 || || hSQ039671 || || MND1 || hSQ039671 || || hHC025653 || 400434	hSQ039671	2.30	0
|| Coatomer protein complex, subunit zeta 2::coatomer protein complex, subunit zeta 2 || Hs.408434 || 51226 || || hSQ019440 || || COPZ2 || hSQ019440 || || hHA032512 || 356028	hSQ019440	2.34	0
|| Fatty acid desaturase 2::fatty acid desaturase 2 || Hs.502745 || 9415 || || hSQ015301 || || FADS2 || hSQ015301 || || hHC018444 || 417071	hSQ015301	2.18	0
|| Ubiquitin-conjugating enzyme E2S::ubiquitin-conjugating enzyme E2S || Hs.396393 || 27338 || NM_014501 || hSQ024815 || || UBE2S || hSQ024815 || || hHR031506 || 377230	hSQ024815	2.09	0
|| Meteorin, glial cell differentiation regulator-like || Hs.591142 || 284207 || NM_001004431 || hSQ004733 || || METRNL || hSQ004733 || || hHR022182 || 376237	hSQ004733	2.20	0
|| Non-SMC condensin I complex, subunit H::non-SMC condensin I complex, subunit H || Hs.308045 || 23397 || || hSQ019273 || || NCAPH || hSQ019273 || || hHC004849 || 329374	hSQ019273	2.17	0
|| Coiled-coil domain containing 28B::coiled-coil domain containing 28B || Hs.534482 || 79140 || || hSQ040299 || || CCDC28B || hSQ040299 || || hHC019157 || 363637	hSQ040299	1.98	0
|| Chromosome 15 open reading frame 23::chromosome 15 open reading frame 23 || Hs.525796 || 90417 || || hSQ020250 || || C15orf23 || hSQ020250 || || hHC009634 || 405814	hSQ020250	2.08	0
|| ZW10 interactor antisense::ZW10 interactor || Hs.591363 || 11130 || || hSQ010592 || || ZWINT || hSQ010592 || || hHC009680 || 386663	hSQ010592	2.53	0
|| ATP synthase, H+ transporting, mitochondrial F0 complex, subunit C1 (subunit 9)::ATP synthase, H+ transporting, mitochondrial F0 complex, subunit C1 (subunit 9) || Hs.80986 || 516 || || hSQ014167 || || ATP5G1 || hSQ014167 || || hHC027858 || 376179	hSQ014167	2.10	0
|| Non-metastatic cells 1, protein (NM23A) expressed in::non-metastatic cells 1, protein (NM23A) expressed in || Hs.463456 || 4830 || || hSQ028945 || || NME1 || hSQ028945 || || hHC017370 || 369304	hSQ028945	1.96	0
|| Solute carrier family 1 (neutral amino acid transporter), member 5::solute carrier family 1 (neutral amino acid transporter), member 5 || Hs.631582 || 6510 || NM_005628 || hSQ001172 || || SLC1A5 || hSQ001172 || || hHR027080 || 392647	hSQ001172	2.05	0
|| Ribonuclease/angiogenin inhibitor 1::ribonuclease/angiogenin inhibitor 1 || Hs.530687 || 6050 || || hSQ027522 || || RNH1 || hSQ027522 || || hHC023728 || 359980	hSQ027522	2.12	0
|| Branched chain aminotransferase 2, mitochondrial::branched chain aminotransferase 2, mitochondrial || Hs.512670 || 587 || || hSQ014745 || || BCAT2 || hSQ014745 || || hHC023378 || 338082	hSQ014745	1.96	0
|| CCAAT/enhancer binding protein (C/EBP), gamma::CCAAT/enhancer binding protein (C/EBP), gamma || Hs.429666 || 1054 || || hSQ033594 || || CEBPG || hSQ033594 || || hHC011095 || 380731	hSQ033594	2.06	0
|| Chromosome 9 open reading frame 140::chromosome 9 open reading frame 140 || Hs.19322 || 89958 || NM_178448 || hSQ022558 || || C9orf140 || hSQ022558 || || hHR029571 || 351780	hSQ022558	2.47	0
|| kinesin family member 20A::Kinesin family member 20A || Hs.73625 || 10112 || || hSQ021466 || || KIF20A || hSQ021466 || || hHC027594 || 373693	hSQ021466	2.10	0
|| PDZ and LIM domain 2 (mystique) || || 64236 || || hSQ040304 || || PDLIM2 || hSQ040304 || || hHA040514 || 342383	hSQ040304	2.08	0
|| growth factor receptor-bound protein 2::Growth factor receptor-bound protein 2 || Hs.444356 || 2885 || || hSQ006601 || || GRB2 || hSQ006601 || || hHA039763 || 384598	hSQ006601	1.87	0
|| hypothetical gene LOC92755 || || 92755 || XM_047083 || hSQ037066 || || LOC92755 || hSQ037066 || || hHR031722 || 373416	hSQ037066	2.03	0
|| TRAF interacting protein::TRAF interacting protein || Hs.517972 || 10293 || || hSQ021306 || || TRAIP || hSQ021306 || || hHC023143 || 383493	hSQ021306	2.06	0
|| WD repeat domain 18::WD repeat domain 18 || Hs.325321 || 57418 || || hSQ001412 || || WDR18 || hSQ001412 || || hHC009178 || 403256	hSQ001412	1.88	0
|| Ectonucleotide pyrophosphatase/phosphodiesterase 2 (autotaxin)::ectonucleotide pyrophosphatase/phosphodiesterase 2 (autotaxin) || Hs.190977 || 5168 || || hSQ023428 || || ENPP2 || hSQ023428 || || hHC012763 || 379056	hSQ023428	2.35	0
|| Tryptophanyl-tRNA synthetase::tryptophanyl-tRNA synthetase || Hs.497599 || 7453 || || hSQ002246 || || WARS || hSQ002246 || || hHC021596 || 387232	hSQ002246	2.10	0
|| HCLS1 associated protein X-1::HCLS1 associated protein X-1 || Hs.199625 || 10456 || NM_006118 || hSQ033589 || || HAX1 || hSQ033589 || || hHR026034 || 375604	hSQ033589	2.00	0
|| Chromosome condensation protein G || Hs.567567 || 64151 || || hSQ034726 || || HCAP-G || hSQ034726 || || hHC019776 || 413615	hSQ034726	2.90	0

|| phosphopantothenoylcysteine decarboxylase::Phosphopantothenoylcysteine decarboxylase || Hs.458922 || 60490 || || hSQ013295 || || PPCDC || hSQ013295 || || hHC017668 || 359492	hSQ013295	1.95	0
|| Nudix (nucleoside diphosphate linked moiety X)-type motif 1::nudix (nucleoside diphosphate linked moiety X)-type motif 1 || Hs.534331 || 4521 || || hSQ024250 || || NUDT1 || hSQ024250 || || hHC024103 || 384787	hSQ024250	2.19	0
|| Mannose-P-dolichol utilization defect 1::mannose-P-dolichol utilization defect 1 || Hs.632249 || 9526 || || hSQ002083 || || MPDU1 || hSQ002083 || || hHC016008 || 414050	hSQ002083	1.96	0
|| 24-dehydrocholesterol reductase::24-dehydrocholesterol reductase || Hs.498727 || 1718 || || hSQ011135 || || DHCR24 || hSQ011135 || || hHC017782 || 407178	hSQ011135	2.63	0
|| Biliverdin reductase B (flavin reductase (NADPH))::biliverdin reductase B (flavin reductase (NADPH)) || Hs.515785 || 645 || || hSQ010366 || || BLVRB || hSQ010366 || || hHC018627 || 352566	hSQ010366	2.03	0
|| V-myb myeloblastosis viral oncogene homolog (avian)-like 1::v-myb myeloblastosis viral oncogene homolog (avian)-like 1 || Hs.445898::Hs.654538 || 4603 || X66087 || hSQ016267 || || MYBL1 || hSQ016267 || || hHR003949 || 334921	hSQ016267	2.01	0
|| Lamin B2::lamin B2 || Hs.538286 || 84823 || || hSQ018478 || || LMNB2 || hSQ018478 || || hHC022111 || 382075	hSQ018478	2.04	0
|| H2A histone family, member Z::H2A histone family, member Z || Hs.119192 || 3015 || NM_002106 || hSQ013595 || || H2AFZ || hSQ013595 || || hHR029248 || 355426	hSQ013595	1.92	0
|| H2A histone family, member X::H2A histone family, member X || Hs.477879 || 3014 || || hSQ001609 || || H2AFX || hSQ001609 || || hHC008264 || 392481	hSQ001609	2.61	0
|| Hematological and neurological expressed 1::hematological and neurological expressed 1 || Hs.532803 || 51155 || || hSQ021138 || || HN1 || hSQ021138 || || hHC027441 || 393053	hSQ021138	2.08	0
|| Karyopherin alpha 2 (RAG cohort 1, importin alpha 1)::karyopherin alpha 2 (RAG cohort 1, importin alpha 1) || Hs.594238 || 3838 || NM_002266 || hSQ043647 || || KPNA2 || hSQ043647 || || hHR029325 || 397598	hSQ043647	1.92	0
|| Sestrin 2::sestrin 2 || Hs.469543 || 83667 || NM_031459 || hSQ033855 || || SESN2 || hSQ033855 || || hHR011399 || 410416	hSQ033855	1.94	0
|| || || 220832 || || hSQ006957 || || XM_165511 || hSQ006957 || || hHR029733::hHR029731 || 380697	hSQ006957	2.12	0
|| Farnesyl diphosphate synthase (farnesyl pyrophosphate synthetase, dimethylallyltranstransferase, geranyltranstransferase)::farnesyl diphosphate synthase (farnesyl pyrophosphate synthetase, dimethylallyltranstransferase, geranyltranstransferase) || Hs.335918 || 2224 || || hSQ029552 || || FDPS || hSQ029552 || || hHC031702 || 406574	hSQ029552	2.10	0
|| Methylenetetrahydrofolate dehydrogenase (NADP+ dependent) 2, methenyltetrahydrofolate cyclohydrolase::methylenetetrahydrofolate dehydrogenase (NADP+ dependent) 2, methenyltetrahydrofolate cyclohydrolase || Hs.469030 || 10797 || || hSQ037566 || || MTHFD2 || hSQ037566 || || hHC019635 || 347646	hSQ037566	2.22	0
|| Thymidylate synthetase::thymidylate synthetase || Hs.592338 || 7298 || || hSQ040228 || || TYMS || hSQ040228 || || hHC006489 || 395874	hSQ040228	2.84	0
|| Coiled-coil domain containing 124::coiled-coil domain containing 124 || Hs.100043 || 115098 || || hSQ000656 || || CCDC124 || hSQ000656 || || hHC025840 || 360978	hSQ000656	2.38	0
|| Polymerase (DNA directed), delta 2, regulatory subunit 50kDa::polymerase (DNA directed), delta 2, regulatory subunit 50kDa || Hs.306791 || 5425 || || hSQ035401 || || POLD2 || hSQ035401 || || hHA033033 || 400618	hSQ035401	1.94	0
|| SCL/TAL1 interrupting locus::SCL/TAL1 interrupting locus || Hs.525198 || 6491 || || hSQ037788 || || STIL || hSQ037788 || || hHC027267 || 352576	hSQ037788	2.08	0
|| Filamin C, gamma (actin binding protein 280)::filamin C, gamma (actin binding protein 280) || Hs.58414 || 2318 || NM_001458 || hSQ007304 || || FLNC || hSQ007304 || || hHR020552 || 391626	hSQ007304	2.09	0
|| F-box and WD repeat domain containing 2::F-box and WD repeat domain containing 2 || Hs.494985 || 26190 || || hSQ014439 || || FBXW2 || hSQ014439 || || hHC025158 || 376386	hSQ014439	1.80	0
|| Fas-activated serine/threonine kinase::Fas-activated serine/threonine kinase || Hs.647094 || 10922 || || hSQ027895 || || FASTK || hSQ027895 || || hHA038594 || 343824	hSQ027895	2.09	0
|| tubulin, beta::Tubulin, beta || Hs.636480 || 203068 || || hSQ019544 || || TUBB || hSQ019544 || || hHC031653 || 396806	hSQ019544	1.99	0
|| Hydrolethalus syndrome 1::hydrolethalus syndrome 1 || Hs.585071 || 219844 || || hSQ027909 || || HYLS1 || hSQ027909 || || hHC003854 || 343528	hSQ027909	1.98	0
|| Chromosome 16 open reading frame 33::chromosome 16 open reading frame 33 || Hs.15277 || 79622 || || hSQ013430 || || C16orf33 || hSQ013430 || || hHC027460 || 359118	hSQ013430	2.05	0
|| Methionyl-tRNA synthetase::methionyl-tRNA synthetase || Hs.632707 || 4141 || || hSQ031932 || || MARS || hSQ031932 || || hHC004609 || 333627	hSQ031932	1.86	0
|| ribosomal protein L39-like::Ribosomal protein L39-like || Hs.647900 || 116832 || || hSQ004572 || || RPL39L || hSQ004572 || || hHC016926 || 374839	hSQ004572	2.11	0
|| Methylenetetrahydrofolate dehydrogenase (NADP+ dependent) 2, methenyltetrahydrofolate cyclohydrolase::methylenetetrahydrofolate dehydrogenase (NADP+ dependent) 2, methenyltetrahydrofolate cyclohydrolase || Hs.469030 || 10797 || || hSQ001867 || || MTHFD2 || hSQ001867 || || hHC028024 || 353639	hSQ001867	2.15	0
|| BCL2-like 12 (proline rich)::BCL2-like 12 (proline rich) || Hs.289052 || 83596 || || hSQ036779 || || BCL2L12 || hSQ036779 || || hHC014134 || 408063	hSQ036779	2.08	0
|| IMP (inosine monophosphate) dehydrogenase 2::IMP (inosine monophosphate) dehydrogenase 2 || Hs.654400 || 3615 || || hSQ015466 || || IMPDH2 || hSQ015466 || || hHC025402 || 403775	hSQ015466	1.83	0
|| Actin, alpha 1, skeletal muscle::actin, alpha 1, skeletal muscle || Hs.1288 || 58 || 58 || hSQ001090 || || ACTA1 || hSQ001090 || || hHC021235 || 384853	hSQ001090	2.22	0
|| Pyrroline-5-carboxylate reductase 1::pyrroline-5-carboxylate reductase 1 || Hs.458332 || 5831 || NM_006907 || hSQ009177 || || PYCR1 || hSQ009177 || || hHR032007 || 349007	hSQ009177	1.96	0
|| Glutamic-oxaloacetic transaminase 1, soluble (aspartate aminotransferase 1)::glutamic-oxaloacetic transaminase 1, soluble (aspartate aminotransferase 1) || Hs.500756 || 2805 || || hSQ029112 || || GOT1 || hSQ029112 || || hHC014582 || 335656	hSQ029112	2.01	0
|| SNRPN upstream reading frame || || 8926 || || hSQ020507 || || SNURF || hSQ020507 || || hHA032938 || 408135	hSQ020507	1.95	0
|| Transcribed locus::tubulin tyrosine ligase-like family, member 1::Tubulin tyrosine ligase-like family, member 1 || Hs.632783::Hs.660298 || 25809 || NM_001008572 || hSQ014950 || || TTLL1 || hSQ014950 || || hHR011922 || 376017	hSQ014950	1.94	0
|| Kinesin family member 11::kinesin family member 11 || Hs.8878 || 3832 || NM_004523 || hSQ038735 || || KIF11 || hSQ038735 || || hHR002672 || 386906	hSQ038735	2.18	0
|| similar to Farnesyl pyrophosphate synthetase (FPP synthetase) (FPS) (Farnesyl diphosphate synthetase) || || 441261 || || hSQ040950 || || LOC441261 || hSQ040950 || || hHR031494::hHR031493 || 369004	hSQ040950	2.14	0
|| Acyl-CoA thioesterase 7::acyl-CoA thioesterase 7 || Hs.126137 || 11332 || || hSQ001386 || || ACOT7 || hSQ001386 || || hHC005819 || 409403	hSQ001386	1.89	0
|| Cystathionine-beta-synthase::cystathionine-beta-synthase || Hs.533013 || 875 || || hSQ013949 || || CBS || hSQ013949 || || hHC025833 || 400611	hSQ013949	2.00	0
|| Sirtuin (silent mating type information regulation 2 homolog) 5 (S. cerevisiae)::sirtuin (silent mating type information regulation 2 homolog) 5 (S. cerevisiae) || Hs.567431 || 23408 || || hSQ031487 || || SIRT5 || hSQ031487 || || hHA036303 || 351165	hSQ031487	1.93	0
|| Serine dehydratase-like::serine dehydratase-like || Hs.337594 || 113675 || || hSQ039269 || || SDSL || hSQ039269 || || hHC025885 || 330834	hSQ039269	2.03	0
|| ADP-ribosylation-like factor 6 interacting protein 4::ADP-ribosylation-like factor 6 interacting protein 4 || Hs.103561 || 51329 || || hSQ037868 || || ARL6IP4 || hSQ037868 || || hHA038793 || 400625	hSQ037868	1.87	0

|| similar to Heterogeneous nuclear ribonucleoprotein A1 (Helix-destabilizing protein) (Single-strand binding protein) (hnRNP core protein A1) (HDP-1) (Topoisomerase-inhibitor suppressed) || || 388275 || || hSQ041257 || || LOC388275 || hSQ041257 || || hHA033262 || 404772	hSQ041257	2.01	0
|| Chromosome 11 open reading frame 48::chromosome 11 open reading frame 48 || Hs.9061 || 79081 || || hSQ039658 || || C11orf48 || hSQ039658 || || hHC030150 || 377294	hSQ039658	1.85	0
|| Thyroid hormone receptor interactor 13::thyroid hormone receptor interactor 13 || Hs.436187 || 9319 || || hSQ007169 || || TRIP13 || hSQ007169 || || hHC019623 || 349839	hSQ007169	2.12	0
|| Cystatin A (stefin A)::cystatin A (stefin A) || Hs.518198 || 1475 || || hSQ008910 || || CSTA || hSQ008910 || || hHC008162 || 343464	hSQ008910	2.32	0
|| Stromal cell-derived factor 2-like 1::stromal cell-derived factor 2-like 1 || Hs.303116 || 23753 || || hSQ042222 || || SDF2L1 || hSQ042222 || || hHC019681 || 334725	hSQ042222	2.21	0
|| Flotillin 1::flotillin 1 || Hs.179986 || 10211 || || hSQ006309 || || FLOT1 || hSQ006309 || || hHC022868 || 392843	hSQ006309	1.82	0
|| Polymerase (DNA directed), delta 2, regulatory subunit 50kDa::polymerase (DNA directed), delta 2, regulatory subunit 50kDa || Hs.306791 || 5425 || || hSQ012875 || || POLD2 || hSQ012875 || || hHC030529 || 333666	hSQ012875	1.85	0
|| Centromere protein N::centromere protein N || Hs.55028::Hs.660033 || 55839 || || hSQ035228 || || CENPN || hSQ035228 || || hHC014704 || 359428	hSQ035228	1.99	0
|| Stanniocalcin 2::stanniocalcin 2 || Hs.233160 || 8614 || || hSQ009520 || || STC2 || hSQ009520 || || hHC006163 || 383911	hSQ009520	2.29	0
|| TRNA 5-methylaminomethyl-2-thiouridylate methyltransferase::tRNA 5-methylaminomethyl-2-thiouridylate methyltransferase || Hs.439524 || 55687 || || hSQ008104 || || TRMU || hSQ008104 || || hHC024582 || 375538	hSQ008104	1.94	0
|| SIL1 homolog, endoplasmic reticulum chaperone (S. cerevisiae)::SIL1 homolog, endoplasmic reticulum chaperone (S. cerevisiae) || Hs.483521 || 64374 || || hSQ020708 || || SIL1 || hSQ020708 || || hHC022963 || 385272	hSQ020708	1.76	0
|| Cell division cycle associated 5::cell division cycle associated 5 || Hs.434886 || 113130 || || hSQ025140 || || CDCA5 || hSQ025140 || || hHC007762 || 401321	hSQ025140	2.01	0
|| Centromere protein H || Hs.631967 || 64946 || || hSQ026210 || || CENPH || hSQ026210 || || hHC006905 || 362562	hSQ026210	1.93	0
|| ubiquitin-conjugating enzyme E2S pseudogene 2 || || 440406 || XM_496186 || hSQ018956 || || UBE2SP2 || hSQ018956 || || hHR031565 || 362801	hSQ018956	2.14	0
|| AA644547_Exon1_286 || || || EST_AA644547 || hSQ012156 || || EST_AA644547 || hSQ012156 || || hHE042545 || 368392	hSQ012156	2.03	0
|| Lysyl oxidase-like 1::lysyl oxidase-like 1 || Hs.65436 || 4016 || || hSQ027519 || || LOXL1 || hSQ027519 || || hHC021711 || 350870	hSQ027519	2.00	0
|| Sirtuin (silent mating type information regulation 2 homolog) 5 (S. cerevisiae)::sirtuin (silent mating type information regulation 2 homolog) 5 (S. cerevisiae) || Hs.567431 || 23408 || || hSQ033247 || || SIRT5 || hSQ033247 || || hHC020557 || 333881	hSQ033247	1.87	0
|| similar to tubulin, beta 5 || || 387922 || XM_370723 || hSQ022779 || || LOC387922 || hSQ022779 || || hHR029757 || 394219	hSQ022779	1.91	0
|| CDC28 protein kinase regulatory subunit 1B::CDC28 protein kinase regulatory subunit 1B || Hs.374378 || 1163 || || hSQ038667 || || CKS1B || hSQ038667 || || hHC031888 || 359326	hSQ038667	1.88	0
|| Cell division cycle 2, G1 to S and G2 to M::cell division cycle 2, G1 to S and G2 to M || Hs.334562 || 983 || || hSQ028778 || || CDC2 || hSQ028778 || || hHC014791 || 383869	hSQ028778	2.08	0
|| Replication protein A3, 14kDa::replication protein A3, 14kDa || Hs.487540 || 6119 || || hSQ031845 || || RPA3 || hSQ031845 || || hHC028599 || 346040	hSQ031845	1.90	0
|| Seryl-tRNA synthetase::seryl-tRNA synthetase || Hs.531176 || 6301 || || hSQ009075 || || SARS || hSQ009075 || || hHC027492 || 415155	hSQ009075	1.89	0
|| Filamin B, beta (actin binding protein 278)::filamin B, beta (actin binding protein 278) || Hs.476448 || 2317 || || hSQ008686 || || FLNB || hSQ008686 || || hHC030937 || 331990	hSQ008686	1.83	0
|| Transgelin::transgelin || Hs.632099 || 6876 || || hSQ041307 || || TAGLN || hSQ041307 || || hHC023384 || 387685	hSQ041307	2.95	0
|| Kinesin family member C1::kinesin family member C1 || Hs.436912 || 3833 || BC073878 || hSQ042495 || || KIFC1 || hSQ042495 || || hHR015552 || 413685	hSQ042495	1.84	0
|| Kinesin light chain 1::kinesin light chain 1 || Hs.20107 || 3831 || || hSQ003372 || || KLC1 || hSQ003372 || || hHC014632 || 357210	hSQ003372	1.84	0
|| thymidine kinase 1, soluble || || 7083 || || hSQ012570 || || TK1 || hSQ012570 || || hHA040614 || 375839	hSQ012570	1.87	0
|| deleted in lymphocytic leukemia, 1 (non-protein coding) || || 10301 || NM_005887 || hSQ022148 || || DLEU1 || hSQ022148 || || hHR008912 || 385605	hSQ022148	1.81	0
|| Solute carrier family 3 (activators of dibasic and neutral amino acid transport), member 2::solute carrier family 3 (activators of dibasic and neutral amino acid transport), member 2 || Hs.502769 || 6520 || || hSQ040992 || || SLC3A2 || hSQ040992 || || hHA039756 || 416862	hSQ040992	2.33	0
|| Polymerase (DNA directed), alpha 2 (70kD subunit)::polymerase (DNA directed), alpha 2 (70kD subunit) || Hs.201897 || 23649 || || hSQ029416 || || POLA2 || hSQ029416 || || hHC014252 || 390950	hSQ029416	1.97	0
|| CDC45 cell division cycle 45-like (S. cerevisiae)::CDC45 cell division cycle 45-like (S. cerevisiae) || Hs.474217 || 8318 || || hSQ005255 || || CDC45L || hSQ005255 || || hHC004040 || 392607	hSQ005255	2.10	0
|| Solute carrier family 25 (mitochondrial carrier; citrate transporter), member 1::solute carrier family 25 (mitochondrial carrier; citrate transporter), member 1 || Hs.111024 || 6576 || || hSQ019146 || || SLC25A1 || hSQ019146 || || hHC024586 || 406626	hSQ019146	1.77	0.04
|| Insulin-like growth factor binding protein 3::insulin-like growth factor binding protein 3 || Hs.450230 || 3486 || || hSQ008857 || || IGFBP3 || hSQ008857 || || hHC001825 || 333767	hSQ008857	1.98	0.04
|| Exosome component 8::exosome component 8 || Hs.294041 || 11340 || || hSQ021927 || || EXOSC8 || hSQ021927 || || hHC023870 || 338919	hSQ021927	1.71	0.04
|| IlvB (bacterial acetolactate synthase)-like::ilvB (bacterial acetolactate synthase)-like || Hs.78880 || 10994 || || hSQ038821 || || ILVBL || hSQ038821 || || hHA036873 || 399984	hSQ038821	1.73	0.04
|| GINS complex subunit 2 (Psf2 homolog)::GINS complex subunit 2 (Psf2 homolog) || Hs.433180 || 51659 || || hSQ014016 || || GINS2 || hSQ014016 || || hHC020837 || 368148	hSQ014016	2.39	0.04
|| Sarcoma antigen NY-SAR-48::sarcoma antigen NY-SAR-48 || Hs.404088 || 93323 || || hSQ010404 || || NY-SAR-48 || hSQ010404 || || hHC019423 || 382330	hSQ010404	1.75	0.04
|| Proteasome (prosome, macropain) subunit, beta type, 10::proteasome (prosome, macropain) subunit, beta type, 10 || Hs.9661 || 5699 || || hSQ018649 || || PSMB10 || hSQ018649 || || hHC021232 || 360539	hSQ018649	2.05	0.04

Genes down-regulated (300)

Gene Name	Gene ID	Fold Change	q-value(%)
|| Stanniocalcin 1::stanniocalcin 1 || Hs.25590 || 6781 || || hSQ017570 || || STC1 || hSQ017570 || || hHC021252 || 415880	hSQ017570	0.10	0
|| hypothetical protein MGC5618 || || 79099 || BC001980 || hSQ019497 || || MGC5618 || hSQ019497 || || hHR019790 || 343709	hSQ019497	0.16	0
|| UDP-glucose ceramide glucosyltransferase::UDP-glucose ceramide glucosyltransferase || Hs.304249 || 7357 || || hSQ035457 || || UGCG || hSQ035457 || || hHC009206 || 336215	hSQ035457	0.17	0
|| ATP-binding cassette, sub-family A (ABC1), member 1::ATP-binding cassette, sub-family A (ABC1), member 1 || Hs.429294 || 19 || 19 || hSQ006427 || || ABCA1 || hSQ006427 || || hHC011506 || 402514	hSQ006427	0.18	0
|| RAS, dexamethasone-induced 1::RAS, dexamethasone-induced 1 || Hs.25829 || 51655 || || hSQ034975 || || RASD1 || hSQ034975 || || hHC007960 || 353733	hSQ034975	0.15	0
|| Phosphoglucomutase 2-like 1::phosphoglucomutase 2-like 1 || Hs.26612 || 283209 || || hSQ022359 || || PGM2L1 || hSQ022359 || || hHC009771 || 345816	hSQ022359	0.19	0
|| Heat shock protein 90kDa beta (Grp94), member 1::heat shock protein 90kDa beta (Grp94), member 1 || Hs.192374 || 7184 || NM_003299 || hSQ043533 || || HSP90B1 || hSQ043533 || || hHR027385 || 330910	hSQ043533	0.18	0
|| F-box protein 32::F-box protein 32 || Hs.403933 || 114907 || || hSQ034286 || || FBXO32 || hSQ034286 || || hHC015112 || 357914	hSQ034286	0.22	0
|| ATPase, H+ transporting, lysosomal 70kDa, V1 subunit A::ATPase, H+ transporting, lysosomal 70kDa, V1 subunit A || Hs.477155 || 523 || || hSQ029860 || || ATP6V1A || hSQ029860 || || hHC013738 || 407501	hSQ029860	0.26	0
|| v-maf musculoaponeurotic fibrosarcoma oncogene homolog B (avian) || Hs.651210 || 9935 || || hSQ006409 || || MAFB || hSQ006409 || || hHC008903 || 409575	hSQ006409	0.21	0
|| Aryl-hydrocarbon receptor nuclear translocator 2::aryl-hydrocarbon receptor nuclear translocator 2 || Hs.459070 || 9915 || || hSQ023704 || || ARNT2 || hSQ023704 || || hHC010715 || 409840	hSQ023704	0.27	0
|| Full-length cDNA clone CS0DB009YL20 of Neuroblastoma Cot 10-normalized of Homo sapiens (human)::syntaxin 17 || Hs.130643::Hs.655706 || 55014 || || hSQ019062 || || STX17 || hSQ019062 || || hHC007977 || 399811	hSQ019062	0.27	0
|| Prostaglandin E receptor 2 (subtype EP2), 53kDa::prostaglandin E receptor 2 (subtype EP2), 53kDa || Hs.2090 || 5732 || || hSQ034350 || || PTGER2 || hSQ034350 || || hHC002732 || 390929	hSQ034350	0.28	0
|| SRY (sex determining region Y)-box 4::Transcribed locus::SRY (sex determining region Y)-box 4 || Hs.357901::Hs.643910::Hs.699195 || 6659 || NM_003107 || hSQ000068 || || SOX4 || hSQ000068 || || hHR003180 || 416374	hSQ000068	0.27	0
|| Nuclear factor of kappa light polypeptide gene enhancer in B-cells inhibitor, alpha::nuclear factor of kappa light polypeptide gene enhancer in B-cells inhibitor, alpha || Hs.81328 || 4792 || || hSQ029264 || || NFKBIA || hSQ029264 || || hHC016471 || 380201	hSQ029264	0.30	0
|| Leukemia inhibitory factor (cholinergic differentiation factor)::leukemia inhibitory factor (cholinergic differentiation factor) || Hs.2250 || 3976 || || hSQ014160 || || LIF || hSQ014160 || || hHC014649 || 395231	hSQ014160	0.24	0
|| Procollagen-lysine, 2-oxoglutarate 5-dioxygenase 2::procollagen-lysine, 2-oxoglutarate 5-dioxygenase 2 || Hs.477866 || 5352 || || hSQ037406 || || PLOD2 || hSQ037406 || || hHA032582 || 341261	hSQ037406	0.27	0
|| Phosphatase and actin regulator 2::phosphatase and actin regulator 2::Phosphatase and actin regulator 2 || Hs.102471::Hs.654921 || 9749 || AB014580 || hSQ040181 || || PHACTR2 || hSQ040181 || || hHR001643 || 411809	hSQ040181	0.22	0
|| KH domain containing, RNA binding, signal transduction associated 1 || Hs.445893::Hs.699176 || 10657 || || hSQ023201 || || KHDRBS1 || hSQ023201 || || hHC012187 || 378236	hSQ023201	0.33	0
|| Superoxide dismutase 2, mitochondrial::superoxide dismutase 2, mitochondrial || Hs.487046 || 6648 || || hSQ034336 || || SOD2 || hSQ034336 || || hHC015362 || 343475	hSQ034336	0.28	0
|| hypothetical LOC284988 || Hs.577599 || 284988 || XM_209429 || hSQ003397 || || LOC284988 || hSQ003397 || || hHR030092 || 390965	hSQ003397	0.29	0
|| Mitogen-activated protein kinase kinase kinase 5::mitogen-activated protein kinase kinase kinase 5 || Hs.186486 || 4217 || || hSQ024305 || || MAP3K5 || hSQ024305 || || hHC017623 || 379788	hSQ024305	0.27	0
|| Ubiquitin specific peptidase 40::ubiquitin specific peptidase 40 || Hs.96513 || 55230 || NM_018218 || hSQ018754 || || USP40 || hSQ018754 || || hHR004976 || 386160	hSQ018754	0.28	0
|| Protein phosphatase 2C, magnesium-dependent, catalytic subunit::protein phosphatase 2C, magnesium-dependent, catalytic subunit || Hs.22265 || 54704 || || hSQ023800 || || PPM2C || hSQ023800 || || hHC003679 || 382435	hSQ023800	0.27	0
|| NEDD4 binding protein 2-like 1::NEDD4 binding protein 2-like 1 || Hs.161220 || 90634 || || hSQ029802 || || N4BP2L1 || hSQ029802 || || hHC010696 || 357760	hSQ029802	0.31	0
|| Hypothetical LOC387763::hypothetical LOC387763 || Hs.530443 || 387763 || BC052560 || hSQ024939 || || LOC387763 || hSQ024939 || || hHR013684 || 355784	hSQ024939	0.25	0
|| Chromosome 1 open reading frame 63::chromosome 1 open reading frame 63 || Hs.259412 || 57035 || || hSQ036347 || || C1orf63 || hSQ036347 || || hHA034254 || 370768	hSQ036347	0.33	0
|| 5'-3' exoribonuclease 2::5'-3' exoribonuclease 2 || Hs.255932 || 22803 || || hSQ043237 || || XRN2 || hSQ043237 || || hHA040270 || 406436	hSQ043237	0.30	0
|| interleukin 6 (interferon, beta 2) || || 3569 || || hSQ023389 || || IL6 || hSQ023389 || || hHA036684 || 416711	hSQ023389	0.31	0
|| Zinc finger protein 292::zinc finger protein 292 || Hs.590890 || 23036 || || hSQ021350 || || ZNF292 || hSQ021350 || || hHC008207 || 411319	hSQ021350	0.29	0
|| Adipose differentiation-related protein::adipose differentiation-related protein || Hs.3416 || 123 || || hSQ003562 || || ADFP || hSQ003562 || || hHC026877 || 394215	hSQ003562	0.35	0
|| Protein tyrosine phosphatase-like A domain containing 1::protein tyrosine phosphatase-like A domain containing 1 || Hs.512973 || 51495 || || hSQ026596 || || PTPLAD1 || hSQ026596 || || hHC010221 || 397027	hSQ026596	0.32	0
|| F-box protein 32::F-box protein 32 || Hs.403933 || 114907 || || hSQ031347 || || FBXO32 || hSQ031347 || || hHA036088 || 352794	hSQ031347	0.33	0
|| Zinc finger protein 521::zinc finger protein 521 || Hs.116935 || 25925 || || hSQ005881 || || ZNF521 || hSQ005881 || || hHC004863 || 347116	hSQ005881	0.32	0
|| RAB14, member RAS oncogene family::RAB14, member RAS oncogene family || Hs.371563 || 51552 || || hSQ033545 || || RAB14 || hSQ033545 || || hHC019744 || 356289	hSQ033545	0.31	0
|| Proprotein convertase subtilisin/kexin type 1::proprotein convertase subtilisin/kexin type 1 || Hs.78977 || 5122 || || hSQ032875 || || PCSK1 || hSQ032875 || || hHC006830 || 343687	hSQ032875	0.26	0
|| Rho-related BTB domain containing 3::Rho-related BTB domain containing 3 || Hs.445030 || 22836 || || hSQ039282 || || RHOBTB3 || hSQ039282 || || hHC014858 || 335996	hSQ039282	0.29	0
|| Ubiquitin-conjugating enzyme E2L 3::ubiquitin-conjugating enzyme E2L 3 || Hs.108104 || 7332 || || hSQ011212 || || UBE2L3 || hSQ011212 || || hHC015035 || 409844	hSQ011212	0.31	0
|| Spermidine/spermine N1-acetyltransferase 1::spermidine/spermine N1-acetyltransferase 1 || Hs.28491 || 6303 || || hSQ016842 || || SAT1 || hSQ016842 || || hHC019362 || 372154	hSQ016842	0.33	0
|| Prostate transmembrane protein, androgen induced 1::prostate transmembrane protein, androgen induced 1 || Hs.517155 || 56937 || || hSQ002811 || || PMEPA1 || hSQ002811 || || hHC008373 || 400412	hSQ002811	0.35	0
|| SEC14 and spectrin domains 1::SEC14 and spectrin domains 1 || Hs.591613 || 91404 || || hSQ010571 || || SESTD1 || hSQ010571 || || hHC010702 || 405381	hSQ010571	0.36	0
|| F-box protein 21::F-box protein 21 || Hs.159699 || 23014 || || hSQ040625 || || FBXO21 || hSQ040625 || || hHC017561 || 361391	hSQ040625	0.33	0

|| Transcribed locus, strongly similar to NP_079355.2 zinc finger CCCH-type containing 12A [Homo sapiens]::zinc finger CCCH-type containing 12A::Zinc finger CCCH-type containing 12A || Hs.471918::Hs.656294 || 80149 || NM_025079 || hSQ012699 || || ZC3H12A || hSQ012699 || || hHR027003 || 350061	hSQ012699	0.35	0
|| B-cell translocation gene 1, anti-proliferative::B-cell translocation gene 1, anti-proliferative || Hs.255935 || 694 || || hSQ009172 || || BTG1 || hSQ009172 || || hHC020460 || 416392	hSQ009172	0.29	0
|| SON DNA binding protein::SON DNA binding protein || Hs.517262 || 6651 || || hSQ022852 || || SON || hSQ022852 || || hHC014106 || 370811	hSQ022852	0.28	0
|| Nicotinamide phosphoribosyltransferase::nicotinamide phosphoribosyltransferase || Hs.489615 || 10135 || NM_005746 || hSQ002812 || || NAMPT || hSQ002812 || || hHA033318::hHR030571 || 375685	hSQ002812	0.35	0
|| Hypothetical protein MGC18216::insulin-like growth factor 1 receptor::Insulin-like growth factor 1 receptor || Hs.595494::Hs.649408::Hs.643120::Hs.699186 || 145815::3480 || BC010607 || hSQ034047 || || MGC18216::IGF1R || hSQ034047 || || hHR008160 || 413535	hSQ034047	0.39	0
|| Family with sequence similarity 108, member C1::family with sequence similarity 108, member C1 || Hs.459072 || 58489 || AK092758 || hSQ012138 || || FAM108C1 || hSQ012138 || || hHR002164 || 355756	hSQ012138	0.37	0
|| 5'-3' exoribonuclease 2::5'-3' exoribonuclease 2 || Hs.255932 || 22803 || || hSQ013681 || || XRN2 || hSQ013681 || || hHA034279 || 384958	hSQ013681	0.34	0
|| AA668647_Exon1_264 || || || EST_AA668647 || hSQ041699 || || EST_AA668647 || hSQ041699 || || hHE042244 || 359664	hSQ041699	0.38	0
|| KIAA1245 || || 149013 || AB033071 || hSQ044353 || || KIAA1245::NBPF14 || hSQ044353 || || hHR026970 || 370695	hSQ044353	0.31	0
|| Zinc finger protein 264::zinc finger protein 264 || Hs.515634 || 9422 || || hSQ014644 || || ZNF264 || hSQ014644 || || hHC006976 || 356539	hSQ014644	0.42	0
|| Inhibitor of DNA binding 2, dominant negative helix-loop-helix protein::inhibitor of DNA binding 2, dominant negative helix-loop-helix protein || Hs.180919 || 3398 || || hSQ001084 || || ID2 || hSQ001084 || || hHC030186 || 375332	hSQ001084	0.39	0
|| myeloid/lymphoid or mixed-lineage leukemia 5 (trithorax homolog, Drosophila) || || 55904 || || hSQ033835 || || MLL5 || hSQ033835 || || hHA035689 || 328039	hSQ033835	0.37	0
|| Protein phosphatase 2C, magnesium-dependent, catalytic subunit::protein phosphatase 2C, magnesium-dependent, catalytic subunit || Hs.22265 || 54704 || || hSQ019800 || || PPM2C || hSQ019800 || || hHC010591 || 382053	hSQ019800	0.32	0
|| Centrosomal protein 350kDa::centrosomal protein 350kDa || Hs.413045 || 9857 || || hSQ029039 || || CEP350 || hSQ029039 || || hHC013226 || 335716	hSQ029039	0.40	0
|| IQ motif and WD repeats 1::IQ motif and WD repeats 1 || Hs.435741 || 55827 || || hSQ019023 || || IQWD1 || hSQ019023 || || hHA032423 || 409975	hSQ019023	0.38	0
|| MAX interactor 1::MAX interactor 1 || Hs.501023 || 4601 || || hSQ034605 || || MXI1 || hSQ034605 || || hHC005992 || 357196	hSQ034605	0.39	0
|| Dual specificity phosphatase 4::dual specificity phosphatase 4 || Hs.417962 || 1846 || || hSQ039152 || || DUSP4 || hSQ039152 || || hHC005874 || 375575	hSQ039152	0.35	0
|| Kelch-like 24 (Drosophila)::kelch-like 24 (Drosophila) || Hs.407709 || 54800 || AK024270 || hSQ001053 || || KLHL24 || hSQ001053 || || hHR003909 || 396270	hSQ001053	0.38	0
|| Solute carrier family 25, member 36::solute carrier family 25, member 36 || Hs.144130 || 55186 || || hSQ030439 || || SLC25A36 || hSQ030439 || || hHC014074 || 404016	hSQ030439	0.35	0
|| Integrin, alpha 2 (CD49B, alpha 2 subunit of VLA-2 receptor)::integrin, alpha 2 (CD49B, alpha 2 subunit of VLA-2 receptor) || Hs.482077 || 3673 || || hSQ003539 || || ITGA2 || hSQ003539 || || hHC020170 || 406484	hSQ003539	0.34	0
|| RAB14, member RAS oncogene family::RAB14, member RAS oncogene family || Hs.371563 || 51552 || NM_016322 || hSQ039232 || || RAB14 || hSQ039232 || || hHR023715 || 351629	hSQ039232	0.35	0
|| GABA(A) receptor-associated protein like 1::GABA(A) receptor-associated protein like 1 || Hs.524250 || 23710 || || hSQ032594 || || GABARAPL1 || hSQ032594 || || hHC027409 || 328664	hSQ032594	0.36	0
|| AA191424_Exon1_336 || || || EST_AA191424 || hSQ001046 || || EST_AA191424 || hSQ001046 || || hHE042760 || 358388	hSQ001046	0.42	0
|| Nuclear factor, interleukin 3 regulated::nuclear factor, interleukin 3 regulated || Hs.79334 || 4783 || || hSQ012521 || || NFIL3 || hSQ012521 || || hHC007947 || 345248	hSQ012521	0.39	0
|| WW and C2 domain containing 2::WW and C2 domain containing 2 || Hs.333179 || 80014 || || hSQ029883 || || WWC2 || hSQ029883 || || hHC006516 || 413333	hSQ029883	0.33	0
|| TRAF family member-associated NFKB activator::TRAF family member-associated NFKB activator || Hs.556496 || 10010 || || hSQ001158 || || TANK || hSQ001158 || || hHA034043 || 410090	hSQ001158	0.40	0
|| Chromosome 5 open reading frame 24::chromosome 5 open reading frame 24 || Hs.406549 || 134553 || || hSQ039074 || || C5orf24 || hSQ039074 || || hHC008151 || 345606	hSQ039074	0.39	0
|| early growth response 1::Early growth response 1 || Hs.326035 || 1958 || || hSQ020199 || || EGR1 || hSQ020199 || || hHC005173 || 327584	hSQ020199	0.34	0
|| 3-phosphoinositide dependent protein kinase-1::3-phosphoinositide dependent protein kinase-1 || Hs.459691 || 5170 || || hSQ004758 || || PDPK1 || hSQ004758 || || hHC023087 || 410582	hSQ004758	0.35	0
|| Collagen, type IV, alpha 3 (Goodpasture antigen) binding protein::collagen, type IV, alpha 3 (Goodpasture antigen) binding protein || Hs.270437 || 10087 || || hSQ011483 || || COL4A3BP || hSQ011483 || || hHC014482 || 400715	hSQ011483	0.39	0
|| Nicotinamide phosphoribosyltransferase::nicotinamide phosphoribosyltransferase || Hs.489615 || 10135 || || hSQ034493 || || NAMPT || hSQ034493 || || hHA033386 || 335748	hSQ034493	0.38	0
|| Phosphoinositide-3-kinase interacting protein 1::phosphoinositide-3-kinase interacting protein 1 || Hs.26670 || 113791 || || hSQ038371 || || PIK3IP1 || hSQ038371 || || hHC014500 || 359103	hSQ038371	0.40	0
|| Pyruvate dehydrogenase kinase, isozyme 4::pyruvate dehydrogenase kinase, isozyme 4 || Hs.8364 || 5166 || || hSQ013196 || || PDK4 || hSQ013196 || || hHC015302 || 341229	hSQ013196	0.37	0
|| Zinc finger protein 36, C3H type-like 1::zinc finger protein 36, C3H type-like 1 || Hs.85155 || 677 || NM_004926 || hSQ010862 || || ZFP36L1 || hSQ010862 || || hHR011357 || 366883	hSQ010862	0.41	0
|| Chromosome 10 open reading frame 10::chromosome 10 open reading frame 10 || Hs.93675 || 11067 || || hSQ044551 || || C10orf10 || hSQ044551 || || hHC021635 || 410538	hSQ044551	0.27	0
|| F-box and leucine-rich repeat protein 11::F-box and leucine-rich repeat protein 11 || Hs.124147 || 22992 || || hSQ017828 || || FBXL11 || hSQ017828 || || hHC016975 || 378880	hSQ017828	0.44	0
|| Family with sequence similarity 108, member C1::family with sequence similarity 108, member C1 || Hs.459072 || 58489 || || hSQ045501 || || FAM108C1 || hSQ045501 || || hHC005006 || 343115	hSQ045501	0.41	0
|| Zinc finger protein 148::zinc finger protein 148 || Hs.592591::Hs.591312 || 7707 || || hSQ011758 || || ZNF148 || hSQ011758 || || hHC012455 || 410109	hSQ011758	0.38	0
|| Hypothetical protein LOC283378::hypothetical protein LOC283378 || Hs.594149 || 283378 || AK092518 || hSQ031429 || || LOC283378 || hSQ031429 || || hHR008242 || 401976	hSQ031429	0.46	0
|| SATB homeobox 1::SATB homeobox 1 || Hs.517717 || 6304 || || hSQ034272 || || SATB1 || hSQ034272 || || hHC003725 || 362110	hSQ034272	0.41	0
|| Ankyrin repeat domain 10::ankyrin repeat domain 10 || Hs.525163 || 55608 || || hSQ028433 || || ANKRD10 || hSQ028433 || || hHC003697 || 329807	hSQ028433	0.44	0
|| Chromosome 5 open reading frame 41::chromosome 5 open reading frame 41 || Hs.484195 || 153222 || NM_153607 || hSQ034566 || || C5orf41 || hSQ034566 || || hHR004147 || 385117	hSQ034566	0.44	0
|| Nicotinamide phosphoribosyltransferase::nicotinamide phosphoribosyltransferase || Hs.489615 || 10135 || || hSQ043000 || || NAMPT || hSQ043000 || || hHA033439 || 381811	hSQ043000	0.42	0

|| Neuroblastoma breakpoint family, member 10::neuroblastoma breakpoint family, member 10 || Hs.515947 || 440673 || || hSQ043971 || || NBPF10 || hSQ043971 || || hHC031736 || 387315	hSQ043971	0.35	0
|| Inositol 1,4,5-triphosphate receptor, type 2::inositol 1,4,5-triphosphate receptor, type 2 || Hs.512235 || 3709 || || hSQ037403 || || ITPR2 || hSQ037403 || || hHC001772 || 389599	hSQ037403	0.45	0
|| General transcription factor IIA, 1-like::Stonin 1 || Hs.44385 || 11037 || || hSQ034583 || || STON1 || hSQ034583 || || hHC002195 || 408777	hSQ034583	0.41	0
|| similar to CG9996-PA || || 389129 || XM_371647 || hSQ024786 || || LOC389129 || hSQ024786 || || hHR007542 || 371688	hSQ024786	0.42	0
|| IQ motif and WD repeats 1::IQ motif and WD repeats 1 || Hs.435741 || 55827 || || hSQ020149 || || IQWD1 || hSQ020149 || || hHC018163 || 384575	hSQ020149	0.44	0
|| Solute carrier family 39 (zinc transporter), member 8::solute carrier family 39 (zinc transporter), member 8 || Hs.288034 || 64116 || || hSQ010342 || || SLC39A8 || hSQ010342 || || hHC006900 || 413639	hSQ010342	0.44	0
|| Family with sequence similarity 63, member B::family with sequence similarity 63, member B || Hs.591122 || 54629 || NM_019092 || hSQ014589 || || FAM63B || hSQ014589 || || hHR006849 || 398285	hSQ014589	0.42	0
|| Proline-rich nuclear receptor coactivator 1::proline-rich nuclear receptor coactivator 1 || Hs.75969 || 10957 || || hSQ005123 || || PNRC1 || hSQ005123 || || hHC005110 || 407552	hSQ005123	0.44	0
|| hypothetical protein LOC284356 || Hs.588994 || 284356 || BC036615 || hSQ019492 || || LOC284356 || hSQ019492 || || hHR025897 || 328495	hSQ019492	0.46	0
|| WD repeat domain 48::Golgi reassembly stacking protein 1, 65kDa || Hs.109778::Hs.695985 || 57599 || || hSQ000234 || || WDR48 || hSQ000234 || || hHC012084 || 413191	hSQ000234	0.46	0
|| Monocyte to macrophage differentiation-associated::monocyte to macrophage differentiation-associated || Hs.463483 || 23531 || || hSQ029511 || || MMD || hSQ029511 || || hHC003590 || 340920	hSQ029511	0.45	0
|| CCAAT/enhancer binding protein (C/EBP), beta::CCAAT/enhancer binding protein (C/EBP), beta || Hs.517106 || 1051 || || hSQ008525 || || CEBPB || hSQ008525 || || hHC007374 || 369359	hSQ008525	0.44	0
|| Zinc finger protein 234 || Hs.334586 || 10780 || || hSQ042238 || || ZNF234 || hSQ042238 || || hHC025882 || 404428	hSQ042238	0.45	0
|| Serpin peptidase inhibitor, clade D (heparin cofactor), member 1::serpin peptidase inhibitor, clade D (heparin cofactor), member 1 || Hs.474270 || 3053 || || hSQ017054 || || SERPIND1 || hSQ017054 || || hHC023995 || 378681	hSQ017054	0.39	0
|| golgi autoantigen, golgin subfamily a-like pseudogene || Hs.498345 || 374650 || NM_198079 || hSQ029960 || || FLJ40113 || hSQ029960 || || hHR029254 || 403227	hSQ029960	0.45	0
|| Phosphoglucomutase 2-like 1::phosphoglucomutase 2-like 1 || Hs.26612 || 283209 || || hSQ039601 || || PGM2L1 || hSQ039601 || || hHC004570 || 402882	hSQ039601	0.44	0
|| Zinc finger protein 451::zinc finger protein 451::KIAA1702 protein || Hs.485628 || 26036::80822 || AB051489 || hSQ033701 || || ZNF451::KIAA1702 || hSQ033701 || || hHR002074 || 403379	hSQ033701	0.43	0
|| Chemokine (C-X-C motif) ligand 1 (melanoma growth stimulating activity, alpha)::chemokine (C-X-C motif) ligand 1 (melanoma growth stimulating activity, alpha) || Hs.789 || 2919 || NM_001511 || hSQ027241 || || CXCL1 || hSQ027241 || || hHR017300 || 391199	hSQ027241	0.36	0
|| Pallidin homolog (mouse)::pallidin homolog (mouse) || Hs.7037 || 26258 || || hSQ042950 || || PLDN || hSQ042950 || || hHC005561 || 362689	hSQ042950	0.45	0
|| Tetratricopeptide repeat domain 14::tetratricopeptide repeat domain 14 || Hs.43213 || 151613 || || hSQ030885 || || TTC14 || hSQ030885 || || hHC013569 || 397643	hSQ030885	0.47	0
|| Zinc finger, AN1-type domain 5::zinc finger, AN1-type domain 5::Full-length cDNA clone CS0DF030YH04 of Fetal brain of Homo sapiens (human) || Hs.406096::Hs.297929 || 7763 || || hSQ012104 || || ZFAND5 || hSQ012104 || || hHC006116 || 387725	hSQ012104	0.46	0
|| KIAA0182::KIAA0182 || Hs.461647 || 23199 || || hSQ035076 || || KIAA0182 || hSQ035076 || || hHC001610 || 335564	hSQ035076	0.44	0
|| ERBB receptor feedback inhibitor 1::ERBB receptor feedback inhibitor 1 || Hs.605445 || 54206 || || hSQ001603 || || ERRFI1 || hSQ001603 || || hHC007581 || 393465	hSQ001603	0.41	0
|| Hypothetical LOC378805::hypothetical LOC378805 || Hs.150556 || 378805 || AK125651 || hSQ001770 || || FLJ43663 || hSQ001770 || || hHR005254 || 403804	hSQ001770	0.46	0
|| Synaptojanin 2::synaptojanin 2 || Hs.434494 || 8871 || || hSQ035832 || || SYNJ2 || hSQ035832 || || hHC005769 || 398764	hSQ035832	0.46	0
|| Nuclear receptor subfamily 2, group F, member 1::nuclear receptor subfamily 2, group F, member 1 || Hs.519445 || 7025 || NM_005654 || hSQ036921 || || NR2F1 || hSQ036921 || || hHR010640 || 386036	hSQ036921	0.46	0
|| TRAF family member-associated NFKB activator::TRAF family member-associated NFKB activator || Hs.556496 || 10010 || || hSQ023302 || || TANK || hSQ023302 || || hHA038285 || 362161	hSQ023302	0.44	0
|| Teashirt zinc finger homeobox 1::teashirt zinc finger homeobox 1 || Hs.284217 || 10194 || || hSQ024797 || || TSHZ1 || hSQ024797 || || hHC006996 || 413716	hSQ024797	0.44	0
|| BTB (POZ) domain containing 11::BTB (POZ) domain containing 11 || Hs.271272 || 121551 || || hSQ015165 || || BTBD11 || hSQ015165 || || hHC012547 || 380802	hSQ015165	0.41	0
|| Kinesin family member 1B::kinesin family member 1B || Hs.97858 || 23095 || || hSQ025883 || || KIF1B || hSQ025883 || || hHC009371 || 409921	hSQ025883	0.48	0
|| Trinucleotide repeat containing 6C::trinucleotide repeat containing 6C || Hs.584945 || 57690 || || hSQ030070 || || TNRC6C || hSQ030070 || || hHC010619 || 409764	hSQ030070	0.45	0
|| Runt-related transcription factor 1 (acute myeloid leukemia 1; aml1 oncogene)::runt-related transcription factor 1 || Hs.149261 || 861 || || hSQ031462 || || RUNX1 || hSQ031462 || || hHC011414 || 337392	hSQ031462	0.48	0
|| PR domain containing 1, with ZNF domain::PR domain containing 1, with ZNF domain || Hs.436023 || 639 || || hSQ011355 || || PRDM1 || hSQ011355 || || hHC004007 || 410256	hSQ011355	0.44	0
|| AU RNA binding protein/enoyl-Coenzyme A hydratase::AU RNA binding protein/enoyl-Coenzyme A hydratase || Hs.175905 || 549 || || hSQ024179 || || AUH || hSQ024179 || || hHC009148 || 357546	hSQ024179	0.48	0
|| Six transmembrane epithelial antigen of the prostate 1::six transmembrane epithelial antigen of the prostate 1 || Hs.61635 || 26872 || || hSQ017383 || || STEAP1 || hSQ017383 || || hHC006334 || 401388	hSQ017383	0.45	0
|| adaptor-related protein complex 1, sigma 2 subunit::Adaptor-related protein complex 1, sigma 2 subunit::Transcribed locus || Hs.656471::Hs.653504 || 8905 || || hSQ008714 || || AP1S2 || hSQ008714 || || hHC025974 || 371312	hSQ008714	0.43	0
|| Tissue factor pathway inhibitor 2::tissue factor pathway inhibitor 2 || Hs.438231 || 7980 || || hSQ037081 || || TFPI2 || hSQ037081 || || hHC004396 || 352668	hSQ037081	0.40	0
|| Proteasome (prosome, macropain) 26S subunit, non-ATPase, 1::proteasome (prosome, macropain) 26S subunit, non-ATPase, 1 || Hs.3887 || 5707 || || hSQ038027 || || PSMD1 || hSQ038027 || || hHA038462 || 339256	hSQ038027	0.47	0
|| Transmembrane and coiled-coil domain family 1::transmembrane and coiled-coil domain family 1 || Hs.477547 || 23023 || || hSQ021889 || || TMCC1 || hSQ021889 || || hHC009368 || 388178	hSQ021889	0.48	0
|| MRNA; cDNA DKFZp686H2138 (from clone DKFZp686H2138)::intercellular adhesion molecule 1 || Hs.643447::Hs.700573 || 3383 || || hSQ008352 || || ICAM1 || hSQ008352 || || hHC018312 || 414736	hSQ008352	0.44	0
|| Methyltransferase like 9::methyltransferase like 9 || Hs.279583 || 51108 || NM_016025 || hSQ045373 || || METTL9 || hSQ045373 || || hHR005662 || 402440	hSQ045373	0.48	0
|| Kinectin 1 (kinesin receptor)::kinectin 1 (kinesin receptor) || Hs.509414 || 3895 || || hSQ020207 || || KTN1 || hSQ020207 || || hHA035102 || 338459	hSQ020207	0.50	0
|| Glypican 4::glypican 4 || Hs.58367 || 2239 || || hSQ030850 || || GPC4 || hSQ030850 || || hHC006690 || 372547	hSQ030850	0.45	0
|| Armadillo repeat containing, X-linked 3::armadillo repeat containing, X-linked 3 || Hs.592225 || 51566 || || hSQ033072 || || ARMCX3 || hSQ033072 || || hHC015301 || 332338	hSQ033072	0.48	0

|| PTPRF interacting protein, binding protein 1 (liprin beta 1)::PTPRF interacting protein, binding protein 1 (liprin beta 1) || Hs.172445 || 8496 || || hSQ001592 || || PPFIBP1 || hSQ001592 || || hHA033655 || 380089	hSQ001592	0.49	0
|| Retinoid X receptor, alpha::retinoid X receptor, alpha || Hs.590886 || 6256 || || hSQ028144 || || RXRA || hSQ028144 || || hHC011015 || 410108	hSQ028144	0.45	0
|| HMG-box transcription factor 1::HMG-box transcription factor 1 || Hs.162032 || 26959 || || hSQ034468 || || HBP1 || hSQ034468 || || hHC004868 || 387971	hSQ034468	0.46	0
|| Prostaglandin E synthase::prostaglandin E synthase || Hs.146688 || 9536 || || hSQ004146 || || PTGES || hSQ004146 || || hHC014163 || 348071	hSQ004146	0.44	0
|| AA999894_Exon1_164 || || || EST_AA999894 || hSQ022970 || || EST_AA999894 || hSQ022970 || || hHE042556 || 358023	hSQ022970	0.43	0
|| Transcribed locus::adaptor-related protein complex 1, sigma 2 subunit::Adaptor-related protein complex 1, sigma 2 subunit || Hs.121592::Hs.656471 || 8905 || NM_003916 || hSQ015481 || || AP1S2 || hSQ015481 || || hHR009633 || 398173	hSQ015481	0.48	0
|| transmembrane 4 L six family member 1 || Hs.696050::Hs.654892 || 4071 || || hSQ043105 || || TM4SF1 || hSQ043105 || || hHC009467 || 409991	hSQ043105	0.41	0
|| family with sequence similarity 101, member B || Hs.591203 || 359845 || NM_182705 || hSQ025310 || || FAM101B || hSQ025310 || || hHR020591 || 410878	hSQ025310	0.44	0
|| tetratricopeptide repeat domain 17::Tetratricopeptide repeat domain 17 || Hs.191186::Hs.696109 || 55761 || NM_018259 || hSQ002824 || || TTC17 || hSQ002824 || || hHR013756 || 357982	hSQ002824	0.49	0
|| ras homolog gene family, member Q || Hs.643481::Hs.695931 || 23433 || NM_012249 || hSQ030170 || || RHOQ || hSQ030170 || || hHR030959 || 411114	hSQ030170	0.50	0
|| breast carcinoma amplified sequence 3::Breast carcinoma amplified sequence 3 || Hs.655028 || 54828 || || hSQ014478 || || BCAS3 || hSQ014478 || || hHA032530 || 401758	hSQ014478	0.45	0
|| Family with sequence similarity 63, member B::family with sequence similarity 63, member B || Hs.591122 || 54629 || || hSQ025686 || || FAM63B || hSQ025686 || || hHC012049 || 329384	hSQ025686	0.44	0
|| Hypothetical protein MGC3032::hypothetical protein MGC3032 || Hs.568945 || 65998 || AK096306 || hSQ041761 || || MGC3032 || hSQ041761 || || hHR025054 || 401408	hSQ041761	0.49	0
|| spermatogenesis associated 13::Spermatogenesis associated 13 || Hs.657121 || 221178 || || hSQ030682 || || SPATA13 || hSQ030682 || || hHC007447 || 379636	hSQ030682	0.50	0
|| Discoidin domain receptor tyrosine kinase 1::discoidin domain receptor tyrosine kinase 1 || Hs.631988 || 780 || || hSQ030565 || || DDR1 || hSQ030565 || || hHC012458 || 336729	hSQ030565	0.48	0
|| Zinc finger and BTB domain containing 43::zinc finger and BTB domain containing 43 || Hs.591903 || 23099 || NM_014007 || hSQ041463 || || ZBTB43 || hSQ041463 || || hHR009372 || 414461	hSQ041463	0.48	0
|| Kruppel-like factor 9::Kruppel-like factor 9 || Hs.150557 || 687 || || hSQ007288 || || KLF9 || hSQ007288 || || hHC003184 || 359409	hSQ007288	0.50	0
|| Chromosome 20 open reading frame 194::chromosome 20 open reading frame 194 || Hs.516853 || 25943 || || hSQ017471 || || C20orf194 || hSQ017471 || || hHC022328 || 388198	hSQ017471	0.48	0
|| folliculin interacting protein 2::Folliculin interacting protein 2 || Hs.652441 || 57600 || || hSQ010715 || || FNIP2 || hSQ010715 || || hHC006887 || 410258	hSQ010715	0.48	0
|| RAB31, member RAS oncogene family::RAB31, member RAS oncogene family || Hs.99528 || 11031 || || hSQ008186 || || RAB31 || hSQ008186 || || hHC026875 || 378683	hSQ008186	0.46	0
|| TRAF family member-associated NFKB activator::TRAF family member-associated NFKB activator || Hs.556496 || 10010 || || hSQ034683 || || TANK || hSQ034683 || || hHA036645 || 395856	hSQ034683	0.44	0
|| Family with sequence similarity 124A::family with sequence similarity 124A || Hs.71913 || 220108 || || hSQ010653 || || FAM124A || hSQ010653 || || hHC011163 || 350032	hSQ010653	0.47	0
|| Chromosome 10 open reading frame 46::chromosome 10 open reading frame 46 || Hs.420024 || 143384 || || hSQ020471 || || C10orf46 || hSQ020471 || || hHC016940 || 390687	hSQ020471	0.47	0
|| B-cell translocation gene 1, anti-proliferative::B-cell translocation gene 1, anti-proliferative || Hs.255935 || 694 || NM_001731 || hSQ022050 || || BTG1 || hSQ022050 || || hHR015364 || 360319	hSQ022050	0.50	0
|| Insulin receptor substrate 2::insulin receptor substrate 2 || Hs.442344 || 8660 || || hSQ034036 || || IRS2 || hSQ034036 || || hHC004062 || 374950	hSQ034036	0.48	0
|| AA056588_Exon1_165 || || || EST_AA056588 || hSQ033185 || || EST_AA056588 || hSQ033185 || || hHE041712 || 416159	hSQ033185	0.49	0
|| AA029098_Exon1_81 || || || EST_AA029098 || hSQ014758 || || EST_AA029098 || hSQ014758 || || hHE041940 || 412926	hSQ014758	0.53	0
|| Family with sequence similarity 107, member B::family with sequence similarity 107, member B || Hs.446315 || 83641 || NM_031453 || hSQ000340 || || FAM107B || hSQ000340 || || hHR014440 || 356940	hSQ000340	0.46	0
|| N-acetyltransferase 12::N-acetyltransferase 12 || Hs.165465 || 122830 || || hSQ014363 || || NAT12 || hSQ014363 || || hHC012923 || 410454	hSQ014363	0.48	0
|| AA425756_Exon1_30 || || || EST_AA425756 || hSQ027260 || || EST_AA425756 || hSQ027260 || || hHE040882 || 401376	hSQ027260	0.50	0
|| Eukaryotic translation initiation factor 3, subunit A::eukaryotic translation initiation factor 3, subunit A || Hs.523299 || 8661 || || hSQ035119 || || EIF3A || hSQ035119 || || hHC026786 || 343363	hSQ035119	0.43	0
|| jumonji, AT rich interactive domain 1A || Hs.654806 || 5927 || || hSQ027978 || || JARID1A || hSQ027978 || || hHC013150 || 400900	hSQ027978	0.50	0
|| Regulator of G-protein signaling 16::regulator of G-protein signaling 16 || Hs.413297 || 6004 || || hSQ042448 || || RGS16 || hSQ042448 || || hHC015652 || 360751	hSQ042448	0.45	0
|| transmembrane protein 132A::Transmembrane protein 132A || Hs.118552 || 54972 || || hSQ007223 || || TMEM132A || hSQ007223 || || hHC011695 || 377520	hSQ007223	0.47	0
|| AA481425_Exon1_304 || || || EST_AA481425 || hSQ015934 || || EST_AA481425 || hSQ015934 || || hHE040990 || 401564	hSQ015934	0.51	0
|| mediator complex subunit 13-like::Mediator complex subunit 13-like || Hs.654691 || 23389 || || hSQ002758 || || MED13L || hSQ002758 || || hHC017268 || 413868	hSQ002758	0.50	0
|| Microphthalmia-associated transcription factor::microphthalmia-associated transcription factor || Hs.166017 || 4286 || || hSQ032521 || || MITF || hSQ032521 || || hHC005278 || 401908	hSQ032521	0.48	0
|| Ubiquitin specific peptidase 36::ubiquitin specific peptidase 36 || Hs.464243 || 57602 || || hSQ033859 || || USP36 || hSQ033859 || || hHC018392 || 388717	hSQ033859	0.51	0
|| Protein kinase C, iota::protein kinase C, iota || Hs.478199 || 5584 || NM_002740 || hSQ014813 || || PRKCI || hSQ014813 || || hHR029288 || 391760	hSQ014813	0.46	0
|| Family with sequence similarity 107, member B::family with sequence similarity 107, member B || Hs.446315 || 83641 || || hSQ008675 || || FAM107B || hSQ008675 || || hHC029691 || 349788	hSQ008675	0.41	0
|| RAS p21 protein activator 4::DNA directed RNA polymerase II polypeptide J-related::RAS p21 protein activator 4::Uroplakin-like protein || Hs.530089::Hs.654726::Hs.700609 || 246721::10156 || NM_006989 || hSQ030194 || || POLR2J2::RASA4 || hSQ030194 || || hHR031234 || 405837	hSQ030194	0.51	0
|| Proteasome (prosome, macropain) 26S subunit, non-ATPase, 1::proteasome (prosome, macropain) 26S subunit, non-ATPase, 1 || Hs.3887 || 5707 || || hSQ031882 || || PSMD1 || hSQ031882 || || hHC014086 || 404078	hSQ031882	0.48	0
|| Chemokine (C-X-C motif) ligand 2::chemokine (C-X-C motif) ligand 2 || Hs.590921 || 2920 || || hSQ027987 || || CXCL2 || hSQ027987 || || hHC018071 || 412350	hSQ027987	0.48	0

|| Cysteine-rich secretory protein LCCL domain containing 2::cysteine-rich secretory protein LCCL domain containing 2 || Hs.513779 || 83716 || || hSQ017088 || || CRISPLD2 || hSQ017088 || || hHC008071 || 384491	hSQ017088	0.48	0
|| Zinc finger protein 281::zinc finger protein 281 || Hs.59757 || 23528 || || hSQ037098 || || ZNF281 || hSQ037098 || || hHC004202 || 337303	hSQ037098	0.46	0
|| G protein-coupled receptor 137B::G protein-coupled receptor 137B || Hs.498160 || 7107 || || hSQ029353 || || GPR137B || hSQ029353 || || hHC010643 || 408630	hSQ029353	0.51	0
|| insulin receptor::Insulin receptor || Hs.465744 || 3643 || || hSQ038527 || || INSR || hSQ038527 || || hHC013670 || 340935	hSQ038527	0.47	0
|| CDC-like kinase 1::CDC-like kinase 1 || Hs.433732 || 1195 || || hSQ000159 || || CLK1 || hSQ000159 || || hHA033158 || 339987	hSQ000159	0.48	0
|| Zinc finger, AN1-type domain 5::zinc finger, AN1-type domain 5::Full-length cDNA clone CS0DF030YH04 of Fetal brain of Homo sapiens (human) || Hs.406096::Hs.297929 || 7763 || || hSQ006551 || || ZFAND5 || hSQ006551 || || hHC012456 || 414655	hSQ006551	0.51	0
|| Furin (paired basic amino acid cleaving enzyme)::furin (paired basic amino acid cleaving enzyme) || Hs.513153 || 5045 || || hSQ015656 || || FURIN || hSQ015656 || || hHC013732 || 360038	hSQ015656	0.51	0
|| SWI/SNF related, matrix associated, actin dependent regulator of chromatin, subfamily c, member 2::SWI/SNF related, matrix associated, actin dependent regulator of chromatin, subfamily c, member 2 || Hs.236030 || 6601 || || hSQ033893 || || SMARCC2 || hSQ033893 || || hHC016830 || 374702	hSQ033893	0.52	0
|| integrin, alpha 1 || || 3672 || || hSQ005741 || || ITGA1 || hSQ005741 || || hHC008666 || 339873	hSQ005741	0.50	0
|| ADP-ribosylation factor-like 5A::ADP-ribosylation factor-like 5A || Hs.470233 || 26225 || || hSQ036348 || || ARL5A || hSQ036348 || || hHC010844 || 386348	hSQ036348	0.51	0
|| RAB31, member RAS oncogene family::RAB31, member RAS oncogene family || Hs.99528 || 11031 || NM_006868 || hSQ001874 || || RAB31 || hSQ001874 || || hHR005814 || 371286	hSQ001874	0.48	0
|| Solute carrier family 43, member 2::solute carrier family 43, member 2 || Hs.160550 || 124935 || || hSQ034540 || || SLC43A2 || hSQ034540 || || hHC005476 || 360579	hSQ034540	0.52	0
|| LATS, large tumor suppressor, homolog 2 (Drosophila)::LATS, large tumor suppressor, homolog 2 (Drosophila) || Hs.78960 || 26524 || || hSQ042337 || || LATS2 || hSQ042337 || || hHC007482 || 369550	hSQ042337	0.48	0
|| Nuclear factor I/C (CCAAT-binding transcription factor)::nuclear factor I/C (CCAAT-binding transcription factor) || Hs.170131 || 4782 || || hSQ006761 || || NFIC || hSQ006761 || || hHC025857 || 398665	hSQ006761	0.47	0
|| Solute carrier family 1 (glial high affinity glutamate transporter), member 3::solute carrier family 1 (glial high affinity glutamate transporter), member 3 || Hs.481918 || 6507 || NM_004172 || hSQ011539 || || SLC1A3 || hSQ011539 || || hHR008032 || 355994	hSQ011539	0.51	0
|| Nuclear receptor subfamily 4, group A, member 1::nuclear receptor subfamily 4, group A, member 1 || Hs.524430 || 3164 || || hSQ026754 || || NR4A1 || hSQ026754 || || hHC010148 || 387343	hSQ026754	0.49	0
|| forkhead box N3 || || 1112 || NM_018589 || hSQ043039 || || FOXN3 || hSQ043039 || || hHR030022 || 404245	hSQ043039	0.53	0
|| Eukaryotic translation initiation factor 4H::general transcription factor II, i, pseudogene 1 || Hs.520943::Hs.654705 || 2970 || BC045632 || hSQ028367 || || GTF2IP1 || hSQ028367 || || hHR030117 || 396672	hSQ028367	0.54	0
|| WW domain containing E3 ubiquitin protein ligase 1::WW domain containing E3 ubiquitin protein ligase 1 || Hs.655189 || 11059 || || hSQ043889 || || WWP1 || hSQ043889 || || hHC029167 || 377806	hSQ043889	0.54	0
|| Secretogranin V (7B2 protein)::secretogranin V (7B2 protein) || Hs.156540 || 6447 || || hSQ025660 || || SCG5 || hSQ025660 || || hHC008407 || 381006	hSQ025660	0.50	0
|| Taurine upregulated gene 1::taurine upregulated gene 1 || Hs.554829 || 55000 || || hSQ014315 || || TUG1 || hSQ014315 || || hHC008761 || 331032	hSQ014315	0.50	0
|| Serine incorporator 3::serine incorporator 3 || Hs.272168 || 10955 || || hSQ017128 || || SERINC3 || hSQ017128 || || hHC013865 || 366438	hSQ017128	0.50	0
|| similar to cysteine protease || || 441556 || XM_497226 || hSQ028317 || || LOC441556 || hSQ028317 || || hHR028405 || 327947	hSQ028317	0.48	0
|| C-type lectin domain family 2, member B::C-type lectin domain family 2, member B || Hs.85201 || 9976 || || hSQ031539 || || CLEC2B || hSQ031539 || || hHC027332 || 389153	hSQ031539	0.44	0
|| Junction plakoglobin::junction plakoglobin || Hs.514174 || 3728 || || hSQ031729 || || JUP || hSQ031729 || || hHC014627 || 410677	hSQ031729	0.50	0
|| GATA zinc finger domain containing 2B::GATA zinc finger domain containing 2B || Hs.4779 || 57459 || || hSQ020596 || || GATAD2B || hSQ020596 || || hHC007615 || 381905	hSQ020596	0.52	0
|| || || 441555 || || hSQ043149 || || XM_497223 || hSQ043149 || || hHR028641::hHR028642 || 398558	hSQ043149	0.49	0
|| Family with sequence similarity 3, member C::family with sequence similarity 3, member C || Hs.434053 || 10447 || NM_014888 || hSQ000580 || || FAM3C || hSQ000580 || || hHR030619 || 377858	hSQ000580	0.56	0
|| Recombination signal binding protein for immunoglobulin kappa J region::recombination signal binding protein for immunoglobulin kappa J region || Hs.479396 || 3516 || || hSQ039547 || || RBPJ || hSQ039547 || || hHA039626 || 336491	hSQ039547	0.50	0
|| Jun B proto-oncogene::jun B proto-oncogene || Hs.25292 || 3726 || || hSQ042645 || || JUNB || hSQ042645 || || hHC004254 || 375221	hSQ042645	0.49	0
|| Cytochrome P450, family 1, subfamily B, polypeptide 1::cytochrome P450, family 1, subfamily B, polypeptide 1 || Hs.154654 || 1545 || || hSQ022198 || || CYP1B1 || hSQ022198 || || hHC002611 || 384981	hSQ022198	0.49	0
|| Signal-regulatory protein alpha::signal-regulatory protein alpha || Hs.581021 || 140885 || NM_080792 || hSQ032348 || || SIRPA || hSQ032348 || || hHR005145 || 395978	hSQ032348	0.53	0
|| Pleiomorphic adenoma gene-like 1::pleiomorphic adenoma gene-like 1 || Hs.444975 || 5325 || || hSQ006318 || || PLAGL1 || hSQ006318 || || hHC012765 || 394617	hSQ006318	0.48	0
|| Oxysterol binding protein-like 8::oxysterol binding protein-like 8 || Hs.430849 || 114882 || NM_020841 || hSQ021281 || || OSBPL8 || hSQ021281 || || hHR022734 || 370752	hSQ021281	0.50	0
|| Heme oxygenase (decycling) 1::heme oxygenase (decycling) 1 || Hs.517581 || 3162 || || hSQ014058 || || HMOX1 || hSQ014058 || || hHC024077 || 362389	hSQ014058	0.48	0
|| Phenazine biosynthesis-like protein domain containing::phenazine biosynthesis-like protein domain containing || Hs.198158 || 64081 || || hSQ000227 || || PBLD || hSQ000227 || || hHC010625 || 363832	hSQ000227	0.49	0
|| Dapper, antagonist of beta-catenin, homolog 1 (Xenopus laevis)::dapper, antagonist of beta-catenin, homolog 1 (Xenopus laevis) || Hs.48950 || 51339 || || hSQ045240 || || DACT1 || hSQ045240 || || hHC001961 || 365846	hSQ045240	0.51	0
|| folliculin interacting protein 2::Folliculin interacting protein 2 || Hs.445342::Hs.652441 || 57600 || AB040883 || hSQ037889 || || FNIP2 || hSQ037889 || || hHR010617 || 395618	hSQ037889	0.49	0
|| Gap junction protein, alpha 1, 43kDa::gap junction protein, alpha 1, 43kDa || Hs.74471 || 2697 || NM_000165 || hSQ014720 || || GJA1 || hSQ014720 || || hHR026571 || 345134	hSQ014720	0.53	0
|| F-box protein 21::F-box protein 21 || Hs.159699 || 23014 || NM_033624 || hSQ044632 || || FBXO21 || hSQ044632 || || hHR002862 || 371133	hSQ044632	0.52	0
|| TNFAIP3 interacting protein 1::TNFAIP3 interacting protein 1 || Hs.543850 || 10318 || || hSQ026675 || || TNIP1 || hSQ026675 || || hHC018692 || 389142	hSQ026675	0.50	0
|| CDC-like kinase 1::CDC-like kinase 1 || Hs.433732 || 1195 || || hSQ031794 || || CLK1 || hSQ031794 || || hHC004116 || 413714	hSQ031794	0.52	0
|| Zinc finger, CCHC domain containing 14::zinc finger, CCHC domain containing 14 || Hs.156231 || 23174 || || hSQ041489 || || ZCCHC14 || hSQ041489 || || hHC007459 || 379702	hSQ041489	0.50	0

|| Cathepsin L-like 3::cathepsin L1 || Hs.418123 || 1514 || || hSQ019251 || || CTSL1 || hSQ019251 || || hHC024794 || 340342	hSQ019251	0.50	0
|| 3'-phosphoadenosine 5'-phosphosulfate synthase 1::3'-phosphoadenosine 5'-phosphosulfate synthase 1 || Hs.368610 || 9061 || || hSQ032650 || || PAPSS1 || hSQ032650 || || hHC005423 || 411386	hSQ032650	0.51	0
|| Interleukin 1 receptor antagonist::interleukin 1 receptor antagonist || Hs.81134 || 3557 || || hSQ031057 || || IL1RN || hSQ031057 || || hHC014624 || 389439	hSQ031057	0.48	0
|| Serum/glucocorticoid regulated kinase 1::serum/glucocorticoid regulated kinase 1 || Hs.510078 || 6446 || || hSQ014179 || || SGK1 || hSQ014179 || || hHC024221 || 366775	hSQ014179	0.51	0
|| BTB and CNC homology 1, basic leucine zipper transcription factor 1::BTB and CNC homology 1, basic leucine zipper transcription factor 1 || Hs.154276 || 571 || NM_206866 || hSQ006549 || || BACH1 || hSQ006549 || || hHR003993 || 396335	hSQ006549	0.54	0
|| Serine incorporator 3::serine incorporator 3 || Hs.272168 || 10955 || || hSQ008030 || || SERINC3 || hSQ008030 || || hHC023281 || 328970	hSQ008030	0.51	0
|| Yippee-like 3 (Drosophila)::yippee-like 3 (Drosophila) || Hs.513491 || 83719 || || hSQ007546 || || YPEL3 || hSQ007546 || || hHC002415 || 352071	hSQ007546	0.52	0
|| Zinc finger protein 331::zinc finger protein 331 || Hs.185674 || 55422 || || hSQ035921 || || ZNF331 || hSQ035921 || || hHC015633 || 394548	hSQ035921	0.54	0
|| Chromosome 10 open reading frame 56::zinc finger, CCHC domain containing 24 || Hs.523080 || 219654 || || hSQ012529 || || C10orf56 || hSQ012529 || || hHC011515 || 379312	hSQ012529	0.54	0
|| Teashirt zinc finger homeobox 3::teashirt zinc finger homeobox 3 || Hs.278436 || 57616 || NM_020856 || hSQ016356 || || TSHZ3 || hSQ016356 || || hHR010267 || 377587	hSQ016356	0.51	0
|| Solute carrier family 30 (zinc transporter), member 7::solute carrier family 30 (zinc transporter), member 7 || Hs.533903 || 148867 || NM_133496 || hSQ024224 || || SLC30A7 || hSQ024224 || || hHR012576 || 413612	hSQ024224	0.53	0
|| Periphilin 1::periphilin 1 || Hs.444157 || 51535 || || hSQ004920 || || PPHLN1 || hSQ004920 || || hHA032642 || 343506	hSQ004920	0.53	0
|| Suppression of tumorigenicity 5::hypothetical protein MGC10850 || Hs.117715::Hs.654940 || 84736 || AK125453 || hSQ031575 || || MGC10850 || hSQ031575 || || hHR005759 || 409363	hSQ031575	0.51	0
|| similar to ARHQ protein || || 442043 || XM_497871 || hSQ032145 || || LOC442043 || hSQ032145 || || hHR030493 || 329062	hSQ032145	0.52	0
|| autism susceptibility candidate 2::Autism susceptibility candidate 2 || Hs.654801::Hs.696023::Hs.700600 || 26053 || || hSQ017175 || || AUTS2 || hSQ017175 || || hHC003332 || 387841	hSQ017175	0.56	0
|| F-box protein 33::F-box protein 33 || Hs.324342 || 254170 || || hSQ013220 || || FBXO33 || hSQ013220 || || hHC003594 || 374346	hSQ013220	0.54	0
|| KH domain containing, RNA binding, signal transduction associated 1 || Hs.445893::Hs.699176 || 10657 || || hSQ000030 || || KHDRBS1 || hSQ000030 || || hHC015434 || 336865	hSQ000030	0.51	0
|| Pleckstrin homology domain containing, family B (evectins) member 2::pleckstrin homology domain containing, family B (evectins) member 2 || Hs.469944::Hs.654873 || 55041 || || hSQ021912 || || PLEKHB2 || hSQ021912 || || hHC015628 || 354339	hSQ021912	0.52	0
|| Sushi, nidogen and EGF-like domains 1::sushi, nidogen and EGF-like domains 1 || Hs.471834 || 25992 || AK074075 || hSQ033288 || || SNED1 || hSQ033288 || || hHR003597 || 397845	hSQ033288	0.51	0
|| AHA1, activator of heat shock 90kDa protein ATPase homolog 2 (yeast)::AHA1, activator of heat shock 90kDa protein ATPase homolog 2 (yeast) || Hs.655602 || 130872 || || hSQ011116 || || AHSA2 || hSQ011116 || || hHC015129 || 395883	hSQ011116	0.53	0
|| Myocyte enhancer factor 2D::myocyte enhancer factor 2D || Hs.314327 || 4209 || || hSQ033215 || || MEF2D || hSQ033215 || || hHC009100 || 353243	hSQ033215	0.54	0
|| Ecotropic viral integration site 2A::ecotropic viral integration site 2A || Hs.591198 || 2123 || || hSQ029373 || || EVI2A || hSQ029373 || || hHC002468 || 388633	hSQ029373	0.51	0
|| Sine oculis binding protein homolog (Drosophila)::sine oculis binding protein homolog (Drosophila) || Hs.445244 || 55084 || || hSQ021119 || || SOBP || hSQ021119 || || hHC010973 || 366369	hSQ021119	0.52	0
|| Solute carrier family 4 (anion exchanger), member 1, adaptor protein::solute carrier family 4 (anion exchanger), member 1, adaptor protein || Hs.306000 || 22950 || NM_018158 || hSQ025907 || || SLC4A1AP || hSQ025907 || || hHR019478 || 336534	hSQ025907	0.56	0
|| NMDA receptor regulated 1::NMDA receptor regulated 1 || Hs.555985 || 80155 || || hSQ008801 || || NARG1 || hSQ008801 || || hHC015050 || 336306	hSQ008801	0.51	0
|| Retinoblastoma-like 2 (p130)::retinoblastoma-like 2 (p130) || Hs.513609 || 5934 || || hSQ032531 || || RBL2 || hSQ032531 || || hHA039849 || 400129	hSQ032531	0.51	0
|| Jumonji domain containing 2A::jumonji domain containing 2A || Hs.155983 || 9682 || || hSQ006784 || || JMJD2A || hSQ006784 || || hHC015725 || 362774	hSQ006784	0.50	0
|| Forkhead box N3::forkhead box N3 || Hs.434286 || 1112 || NM_005197 || hSQ029083 || || FOXN3 || hSQ029083 || || hHR004467 || 346485	hSQ029083	0.55	0
|| Adenosine monophosphate deaminase (isoform E)::adenosine monophosphate deaminase (isoform E) || Hs.501890 || 272 || || hSQ011349 || || AMPD3 || hSQ011349 || || hHC011935 || 381912	hSQ011349	0.52	0
|| C-terminal binding protein 2::C-terminal binding protein 2 || Hs.501345 || 1488 || NM_022802 || hSQ035269 || || CTBP2 || hSQ035269 || || hHR029704 || 358221	hSQ035269	0.55	0
|| WW domain containing transcription regulator 1::WW domain containing transcription regulator 1 || Hs.477921::Hs.655312::Hs.699296 || 25937 || || hSQ032286 || || WWTR1 || hSQ032286 || || hHC005553 || 394534	hSQ032286	0.53	0
|| BH3 interacting domain death agonist::BH3 interacting domain death agonist || Hs.591054 || 637 || || hSQ000282 || || BID || hSQ000282 || || hHC009923 || 411508	hSQ000282	0.55	0
|| Serpin peptidase inhibitor, clade I (neuroserpin), member 1::serpin peptidase inhibitor, clade I (neuroserpin), member 1 || Hs.478153 || 5274 || || hSQ025725 || || SERPINI1 || hSQ025725 || || hHC002254 || 405615	hSQ025725	0.55	0
|| interferon regulatory factor 2 binding protein 2 || Hs.350268 || 359948 || || hSQ042850 || || IRF2BP2 || hSQ042850 || || hHC011239 || 384916	hSQ042850	0.54	0
|| Nance-Horan syndrome (congenital cataracts and dental anomalies)::Nance-Horan syndrome (congenital cataracts and dental anomalies) || Hs.201623 || 4810 || || hSQ008315 || || NHS || hSQ008315 || || hHC007570 || 401080	hSQ008315	0.55	0
|| ras homolog gene family, member Q || Hs.643481::Hs.695931 || 23433 || || hSQ022966 || || RHOQ || hSQ022966 || || hHC031275 || 345705	hSQ022966	0.52	0
|| BCL2/adenovirus E1B 19kDa interacting protein 3-like::BCL2/adenovirus E1B 19kDa interacting protein 3-like || Hs.131226 || 665 || || hSQ035040 || || BNIP3L || hSQ035040 || || hHC027849 || 399450	hSQ035040	0.55	0
|| interferon (alpha, beta and omega) receptor 2 || Hs.642682::Hs.654564::Hs.695963 || 3455 || NM_207585 || hSQ017018 || || IFNAR2 || hSQ017018 || || hHR016413 || 393724	hSQ017018	0.49	0
|| Zinc finger protein 275::zinc finger protein 275 || Hs.348963 || 10838 || AK122976 || hSQ021928 || || ZNF275 || hSQ021928 || || hHR007376 || 385467	hSQ021928	0.53	0
|| Interleukin 7 receptor::interleukin 7 receptor || Hs.591742 || 3575 || || hSQ019362 || || IL7R || hSQ019362 || || hHC010514 || 338209	hSQ019362	0.54	0
|| Interleukin 4 receptor::interleukin 4 receptor || Hs.513457 || 3566 || || hSQ028660 || || IL4R || hSQ028660 || || hHC010165 || 326557	hSQ028660	0.52	0
|| Tribbles homolog 1 (Drosophila)::tribbles homolog 1 (Drosophila) || Hs.444947 || 10221 || || hSQ021134 || || TRIB1 || hSQ021134 || || hHC003510 || 372065	hSQ021134	0.50	0
|| AA029434_Exon1_190 || || || EST_AA029434 || hSQ003364 || || EST_AA029434 || hSQ003364 || || hHE041600 || 390917	hSQ003364	0.53	0

|| Oxidation resistance 1::oxidation resistance 1 || Hs.148778 || 55074 || || hSQ019279 || || OXR1 || hSQ019279 || || hHC030259 || 383442	hSQ019279	0.51	0
|| WD repeat domain 19::WD repeat domain 19 || Hs.438482 || 57728 || || hSQ033036 || || WDR19 || hSQ033036 || || hHC005700 || 416087	hSQ033036	0.57	0
|| Chromosome 5 open reading frame 4::chromosome 5 open reading frame 4 || Hs.519694 || 10826 || || hSQ032707 || || C5orf4 || hSQ032707 || || hHC008122 || 403755	hSQ032707	0.51	0
|| PX domain containing serine/threonine kinase::PX domain containing serine/threonine kinase || Hs.190544 || 54899 || || hSQ029982 || || PXK || hSQ029982 || || hHC022238 || 338596	hSQ029982	0.52	0
|| PHD finger protein 10::PHD finger protein 10 || Hs.435933 || 55274 || || hSQ012984 || || PHF10 || hSQ012984 || || hHC003152 || 385597	hSQ012984	0.57	0
|| Dehydrogenase/reductase (SDR family) X-linked::dehydrogenase/reductase (SDR family) X-linked::zinc finger, BED-type containing 1 || Hs.131452 || 207063::9189 || NM_004729 || hSQ026774 || || DHRSX::ZBED1 || hSQ026774 || || hHR031259 || 410166	hSQ026774	0.57	0
|| numb homolog (Drosophila)::Numb homolog (Drosophila) || Hs.654609 || 8650 || || hSQ022200 || || NUMB || hSQ022200 || || hHC002421 || 400141	hSQ022200	0.52	0
|| Receptor (G protein-coupled) activity modifying protein 1::receptor (G protein-coupled) activity modifying protein 1 || Hs.471783 || 10267 || || hSQ032724 || || RAMP1 || hSQ032724 || || hHC015430 || 403951	hSQ032724	0.44	0
|| RNA binding motif protein 39::RNA binding motif protein 39 || Hs.282901 || 9584 || NM_184244 || hSQ027825 || || RBM39 || hSQ027825 || || hHR012169 || 327203	hSQ027825	0.49	0
|| Synaptotagmin binding, cytoplasmic RNA interacting protein::synaptotagmin binding, cytoplasmic RNA interacting protein || Hs.571177 || 10492 || || hSQ038752 || || SYNCRIP || hSQ038752 || || hHA033344 || 389466	hSQ038752	0.49	0
|| Adipocyte-specific adhesion molecule::adipocyte-specific adhesion molecule || Hs.591949 || 79827 || || hSQ029061 || || ASAM || hSQ029061 || || hHC006121 || 326765	hSQ029061	0.49	0
|| Lipopolysaccharide-induced TNF factor::lipopolysaccharide-induced TNF factor || Hs.459940 || 9516 || || hSQ030786 || || LITAF || hSQ030786 || || hHC012167 || 409880	hSQ030786	0.56	0
|| Zinc finger protein 652::zinc finger protein 652 || Hs.463375 || 22834 || AB023141 || hSQ006583 || || ZNF652 || hSQ006583 || || hHR008989 || 329170	hSQ006583	0.47	0
|| Phosphatidylinositol transfer protein, cytoplasmic 1::phosphatidylinositol transfer protein, cytoplasmic 1 || Hs.591185 || 26207 || || hSQ020311 || || PITPNC1 || hSQ020311 || || hHC018054 || 376503	hSQ020311	0.52	0
|| Nicotinamide phosphoribosyltransferase::nicotinamide phosphoribosyltransferase || Hs.489615::Hs.592288 || 10135 || NM_005746 || hSQ029679 || || NAMPT || hSQ029679 || || hHR025500 || 414706	hSQ029679	0.51	0
|| RAB GTPase activating protein 1::RAB GTPase activating protein 1 || Hs.271341 || 23637 || NM_012197 || hSQ023796 || || RABGAP1 || hSQ023796 || || hHR009009 || 393404	hSQ023796	0.57	0
|| Sushi, nidogen and EGF-like domains 1::sushi, nidogen and EGF-like domains 1 || Hs.471834 || 25992 || || hSQ025816 || || SNED1 || hSQ025816 || || hHC003598 || 405497	hSQ025816	0.52	0
|| BCL2-related protein A1::BCL2-related protein A1 || Hs.227817 || 597 || || hSQ026753 || || BCL2A1 || hSQ026753 || || hHC010622 || 338428	hSQ026753	0.46	0
|| hypothetical protein LOC286161::Hypothetical protein LOC286161 || Hs.370450::Hs.696258 || 286161 || || hSQ030047 || || LOC286161 || hSQ030047 || || hHC008640 || 413896	hSQ030047	0.56	0
|| Topoisomerase I binding, arginine/serine-rich::topoisomerase I binding, arginine/serine-rich || Hs.589962 || 10210 || || hSQ038698 || || TOPORS || hSQ038698 || || hHC003509 || 363894	hSQ038698	0.54	0
|| microtubule-associated protein 1B::Microtubule-associated protein 1B || Hs.584777::Hs.637017 || 4131 || || hSQ020921 || || MAP1B || hSQ020921 || || hHC007931 || 409920	hSQ020921	0.50	0
|| Hexamethylene bis-acetamide inducible 1::hexamethylene bis-acetamide inducible 1 || Hs.15299 || 10614 || || hSQ032810 || || HEXIM1 || hSQ032810 || || hHC012899 || 411586	hSQ032810	0.57	0
|| ATPase, class II, type 9A::Transcribed locus || Hs.592144::Hs.649234::Hs.700629 || 10079 || AB014511 || hSQ023334 || || ATP9A || hSQ023334 || || hHR011812 || 359359	hSQ023334	0.56	0
|| Protein phosphatase 2 (formerly 2A), regulatory subunit A, beta isoform::protein phosphatase 2 (formerly 2A), regulatory subunit A, beta isoform || Hs.584790 || 5519 || || hSQ041845 || || PPP2R1B || hSQ041845 || || hHA038509 || 334989	hSQ041845	0.56	0
|| Cytoplasmic FMR1 interacting protein 2::cytoplasmic FMR1 interacting protein 2 || Hs.519702 || 26999 || || hSQ036764 || || CYFIP2 || hSQ036764 || || hHC024641 || 363308	hSQ036764	0.57	0
|| Synaptojanin 2 binding protein::synaptojanin 2 binding protein || Hs.443661 || 55333 || || hSQ000736 || || SYNJ2BP || hSQ000736 || || hHC003692 || 390251	hSQ000736	0.53	0
|| V-maf musculoaponeurotic fibrosarcoma oncogene homolog F (avian)::v-maf musculoaponeurotic fibrosarcoma oncogene homolog F (avian) || Hs.517617 || 23764 || || hSQ016093 || || MAFF || hSQ016093 || || hHC004541 || 365352	hSQ016093	0.49	0
|| Damage-regulated autophagy modulator::damage-regulated autophagy modulator || Hs.525634 || 55332 || || hSQ017535 || || DRAM || hSQ017535 || || hHC013750 || 407564	hSQ017535	0.56	0
|| GATA zinc finger domain containing 1::GATA zinc finger domain containing 1 || Hs.21145 || 57798 || || hSQ020656 || || GATAD1 || hSQ020656 || || hHC006077 || 365493	hSQ020656	0.52	0
|| Zinc finger protein 573::zinc finger protein 573 || Hs.531262 || 126231 || || hSQ009877 || || ZNF573 || hSQ009877 || || hHC006632 || 392220	hSQ009877	0.50	0
|| Tripartite motif-containing 73 || Hs.632307 || 375593 || NM_198924 || hSQ008894 || || TRIM73 || hSQ008894 || || hHR031365 || 396433	hSQ008894	0.53	0
|| Periphilin 1::periphilin 1 || Hs.444157 || 51535 || || hSQ041604 || || PPHLN1 || hSQ041604 || || hHA035264 || 390113	hSQ041604	0.53	0
|| CAP, adenylate cyclase-associated protein, 2 (yeast)::CAP, adenylate cyclase-associated protein, 2 (yeast) || Hs.132902 || 10486 || || hSQ041713 || || CAP2 || hSQ041713 || || hHC017734 || 403212	hSQ041713	0.49	0.04
|| Bone morphogenetic protein 6::bone morphogenetic protein 6 || Hs.285671 || 654 || || hSQ004128 || || BMP6 || hSQ004128 || || hHC014103 || 345884	hSQ004128	0.50	0.04
|| RNA, 7SK, nuclear || || 125050 || || hSQ036657 || || RN7SK || hSQ036657 || || hHR030227 || 409348	hSQ036657	0.47	0.04
|| AA455133_Exon1_19 || || || EST_AA455133 || hSQ003718 || || EST_AA455133 || hSQ003718 || || hHE042199 || 379952	hSQ003718	0.53	0.04
|| patatin-like phospholipase domain containing 8::Patatin-like phospholipase domain containing 8 || Hs.617340 || 50640 || || hSQ028959 || || PNPLA8 || hSQ028959 || || hHA035019 || 344848	hSQ028959	0.51	0.04
|| Anthrax toxin receptor 1::anthrax toxin receptor 1 || Hs.165859 || 84168 || || hSQ028735 || || ANTXR1 || hSQ028735 || || hHA034224 || 415928	hSQ028735	0.56	0.04
|| topoisomerase (DNA) I || Hs.592136 || 7150 || NM_003286 || hSQ022029 || || TOP1 || hSQ022029 || || hHR030026 || 337204	hSQ022029	0.55	0.04
|| Hect domain and RLD 2 pseudogene::hect domain and RLD 2 pseudogene::Hect domain and RLD 2 pseudogene 2::hect domain and RLD 2 pseudogene 2 || Hs.531509::Hs.458334 || 440248::400322 || AF041080 || hSQ003286 || || LOC440248::HERC2P2 || hSQ003286 || || hHR029938 || 402152	hSQ003286	0.57	0.04
|| Hypothetical protein LOC161527::Hypothetical protein LOC161527 || Hs.534573 || 161527 || BX647899 || hSQ023797 || || LOC161527::PML || hSQ023797 || || hHR030193 || 330762	hSQ023797	0.57	0.04
|| AT rich interactive domain 5A (MRF1-like)::AT rich interactive domain 5A (MRF1-like) || Hs.920 || 10865 || || hSQ015635 || || ARID5A || hSQ015635 || || hHC020512 || 355272	hSQ015635	0.50	0.04
