# Supplementary material for: IGF-I induced genes in stromal fibroblasts predict the clinical outcome of breast and lung cancer patients
Source: BMC Med. 2010 Jan 5;8:1. doi: 10.1186/1741-7015-8-1 (PMC2823652; doi:10.1186/1741-7015-8-1)

Supplementary data figure 6. Relationship of expression level of "breast fibroblast derived IGF-I signature" with distant metastasis free and overall survival applying continuous scoring.

A. Continuous score based on average expression level of the signature in NKI patients. Colors correspond to score below (yellow) or above (blue) the median (red line). Overall (B) and metastasis free survival (C) analysis using a continuous score resulting from "breast fibroblast derived IGF-I signature" in early stage breast cancer patients from the NKI.

A

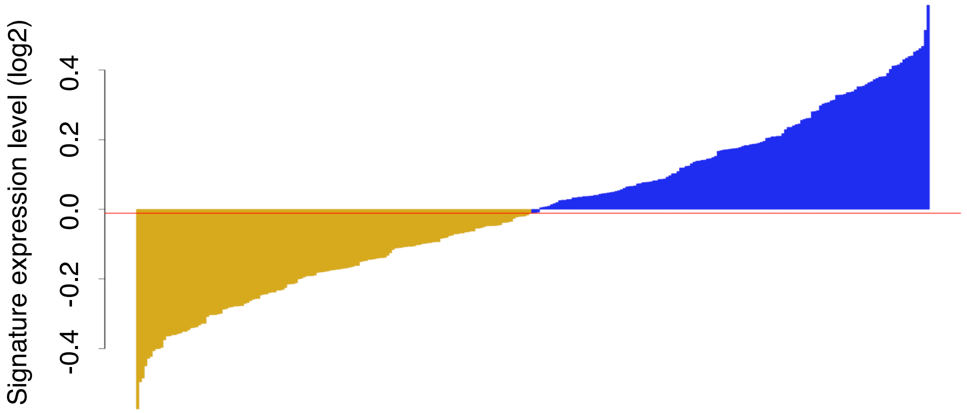

B C

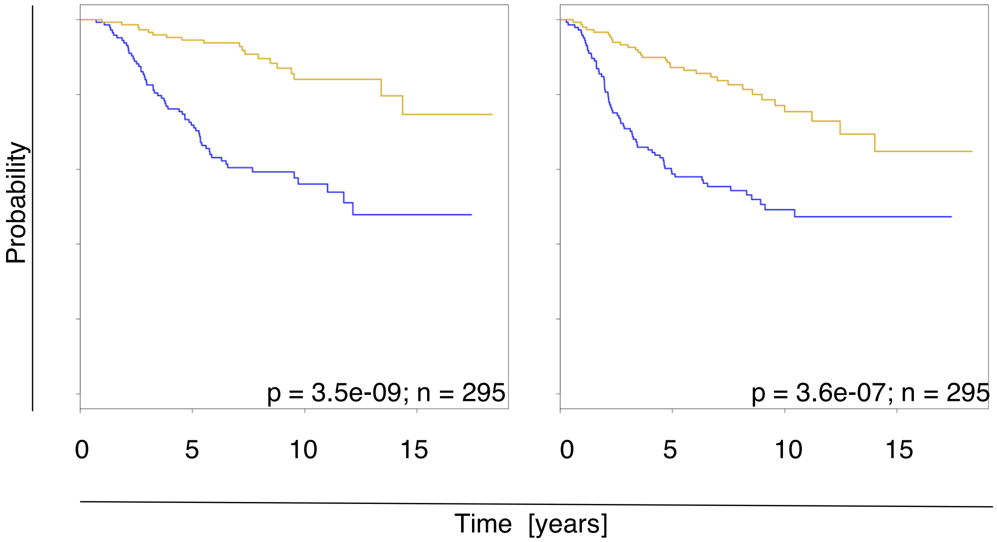

Supplement: Additional file 7 — Figure S6. Relationship of expression level of breast fibroblast derived insulin-like growth factor-1 (IGF-I) signature with distant metastasis free and overall survival applying continuous scoring. A. Continuous score based on average expression level of the signature in Netherlands Cancer Institute (NKI) patients. Colours correspond to score below (yellow) or above (blue) the median (red line). Overall (B) and metastasis free survival (C) analysis using a continuous score resulting from breast fibroblast derived IGF-I signature in early stage breast cancer patients from the NKI. [file 1741-7015-8-1-S7.PDF]
