# Supplementary material for: IGF-I induced genes in stromal fibroblasts predict the clinical outcome of breast and lung cancer patients
Source: BMC Med. 2010 Jan 5;8:1. doi: 10.1186/1741-7015-8-1 (PMC2823652; doi:10.1186/1741-7015-8-1)
Supplement: Additional file 8 — Table S3. The detailed list of correlation values of breast fibroblast derived insulin-like growth factor-1 (IGF-I) signature to the previously published signatures and fibroblast derived IGF-I signature. [file 1741-7015-8-1-S8.PDF]

Published signature:	Breast fibroblasts IGF - I derived signature.
Hypoxia	0.17
Interferon	0.43
Cor.Basal	0.60
Cor.ERBB2	0.38
Wound	**0.76**
Cor.LumB	0.69
Death_2005	0.33
MCR_2005	0.19
X70.genes	**-0.74**
Cor.LumA	-0.49
Cor.Normal	-0.40
Fibroblasts derived signature	**0.77**
