# Supplementary material for: IGF-I induced genes in stromal fibroblasts predict the clinical outcome of breast and lung cancer patients
Source: BMC Med. 2010 Jan 5;8:1. doi: 10.1186/1741-7015-8-1 (PMC2823652; doi:10.1186/1741-7015-8-1)

Supplementary data figure 7. Relationship of expression level of "breast fibroblast derived IGF-I signature" with overall survival and disease specific survival applying continuous scoring.

A. Continuous score based on average expression level of the signature in Bhattacharjee dataset patients. Colors correspond to score below (yellow) or above (blue) the median (red line). Overall (B) and disease specific survival (C) analysis using a continuous score resulting from "breast fibroblast derived IGF-I signature" in Bhattacharjee dataset patients.

A

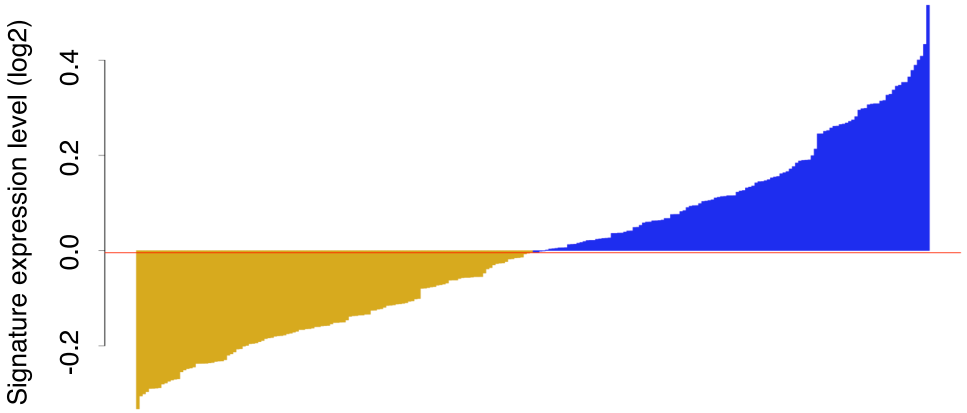

B

C

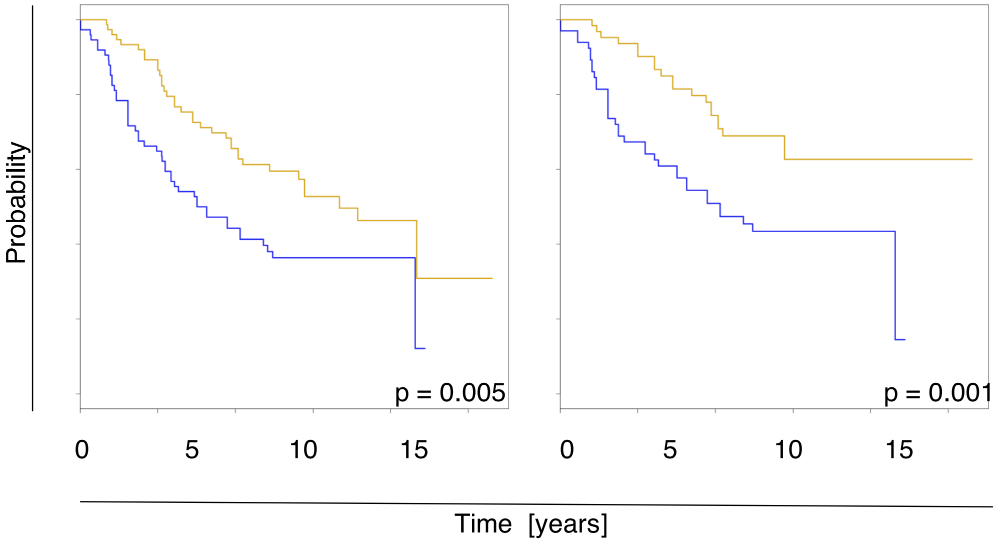

Supplement: Additional file 9 — Figure S7. Relationship of expression level of breast fibroblast derived insulin-like growth factor-1 (IGF-I) signature with overall survival and disease specific survival applying continuous scoring. A. Continuous score based on average expression level of the signature in Bhattacharjee dataset patients. Colours correspond to score below (yellow) or above (blue) the median (red line). Overall (B) and disease specific survival (C) analysis using a continuous score resulting from breast fibroblast derived IGF-I signature in Bhattacharjee dataset patients. [file 1741-7015-8-1-S9.PDF]
